# Spiderweb-inspired electronically conductive hygroscopic adhesive for chronic bioelectronic interface

**DOI:** 10.1126/sciadv.aea3748

**Published:** 2026-08-21

**Authors:** Lingxuan Kong, Fulin Wang, Hanqi Wen, Chan Wang, Zheye Zhang, Lewen Zheng, Zhuoming Liang, Yuxin Liu, Peng Chen

**Affiliations:** ^1^ School of Chemistry, Chemical Engineering and Biotechnology, Nanyang Technological University, Singapore 637457, Singapore.; ^2^ Institute of Flexible Electronics Technology of THU, Jiaxing, Zhejiang 314000, China.; ^3^ Department of Biomedical Engineering (BME), National University of Singapore, Singapore 117583, Singapore.; ^4^ Institute for Health Innovation and Technology (iHealthtech), National University of Singapore, Singapore 117599, Singapore.; ^5^ The N.1 Institute for Health, National University of Singapore, Singapore 117456, Singapore.; ^6^ Lee Kong Chian School of Medicine, Institute for Digital Molecular Analytics and Science (IDMxS), Nanyang Technological University, Singapore 636921, Singapore.; ^7^ Skin Research Institute of Singapore, Singapore 308232, Singapore.

## Abstract

Long-term physiological signal monitoring is critical for accurate diagnosis and effective management of chronic diseases. However, current electrode technologies face significant drawbacks: dry electrodes exhibit high electrochemical impedance and poor adhesion to skin, while wet electrodes suffer from short service lifetimes due to water loss. Inspired by the hygroscopic properties of spider webs, we developed an electronically conductive hygroscopic adhesive (termed as “hygrotrode”) that continuously absorbs ambient moisture through a cross-linked, three-dimensional hygroscopic polymer network. This design maintains a low interface impedance and high adhesion energy for over a year, facilitated by absorbed intermediate water that electrochemically bridges the conducting polymer and skin tissue. The specific impedance of hygrotrode is 82.4 times lower compared with wet electrodes after 1 year. We further demonstrate hygrotrode’s suitability for chronic biopotential monitoring and electromyography-based gesture recognition, highlighting its wide-ranging applicability in long-term wearable electronics and human-machine interfaces.

## INTRODUCTION

Access to high-fidelity and longitudinal medical data is crucial for accurately diagnosing, monitoring, and treating various diseases (*1*–*3*). In particular, home-based wearable electronics are transforming remote patient monitoring and preventive medicine, necessitating long-term stable interfaces between skin and electronic sensors (*4*–*6*). The skin-device interfaces must provide medical-grade biophysical, bioelectrical, and biochemical data over extended periods, spanning weeks or even months (*7*–*9*). For instance, monitoring arrhythmias requires prolonged and repetitive use of electrodes to capture sporadic cardiac events in electrocardiogram (ECG) recording (*10*).

Achieving chronically stable electrode performance while maintaining high signal quality remains a significant challenge yet to be solved. Dry electrodes, typically composed of metal or carbon materials, can maintain stable performance over long-term use (*11*, *12*). However, the electrochemical mismatch between electrode materials and skin tissue results in high impedance and elevated noise level (*13*). Hydrogel-based wet electrodes provide a low-impedance bioelectronic interface with skin tissue, offering a high signal-to-noise ratio (SNR) and broad clinical applicability. However, a critical limitation is their poor stability due to continuous water evaporation into ambient environment. The continuous reduction in water content and changes in the state of water molecules, characterized by their affinity to polymer chains and their mobility within the matrix, lead to progressively declining electrode adhesion and increasing electrochemical impedance (*14*, *15*).

We reason that hygroscopic materials can be rationally designed to maintain water sorption-desorption equilibrium and thus form a permanent, nonevaporating electronic interface with biological tissue. Here, we report a bio-inspired electronically conductive hygroscopic adhesive (hygrotrode) with stable electrode performance and low electrochemical impedance. Strong electrostatic interactions between water molecules and quaternary ammonium groups within the hygrotrode endow the system with intrinsic hygroscopicity, while the regulated intermediate-to-bound water (BW) ratio enables improved water permeability, interfacial adhesion, and electrochemical performance. After 1-year exposure to ambient condition, hygrotrode showed ∼82.4 times and ∼38.9 times lower area-specific impedance (ASI) at 10 Hz compared to wet electrode and dry electrode respectively, yielding substantially better gesture recognition accuracy based on electromyogram (EMG) signals.

## RESULTS

### Design and mechanism of bio-inspired hygrotrode

**Fig. 1.**
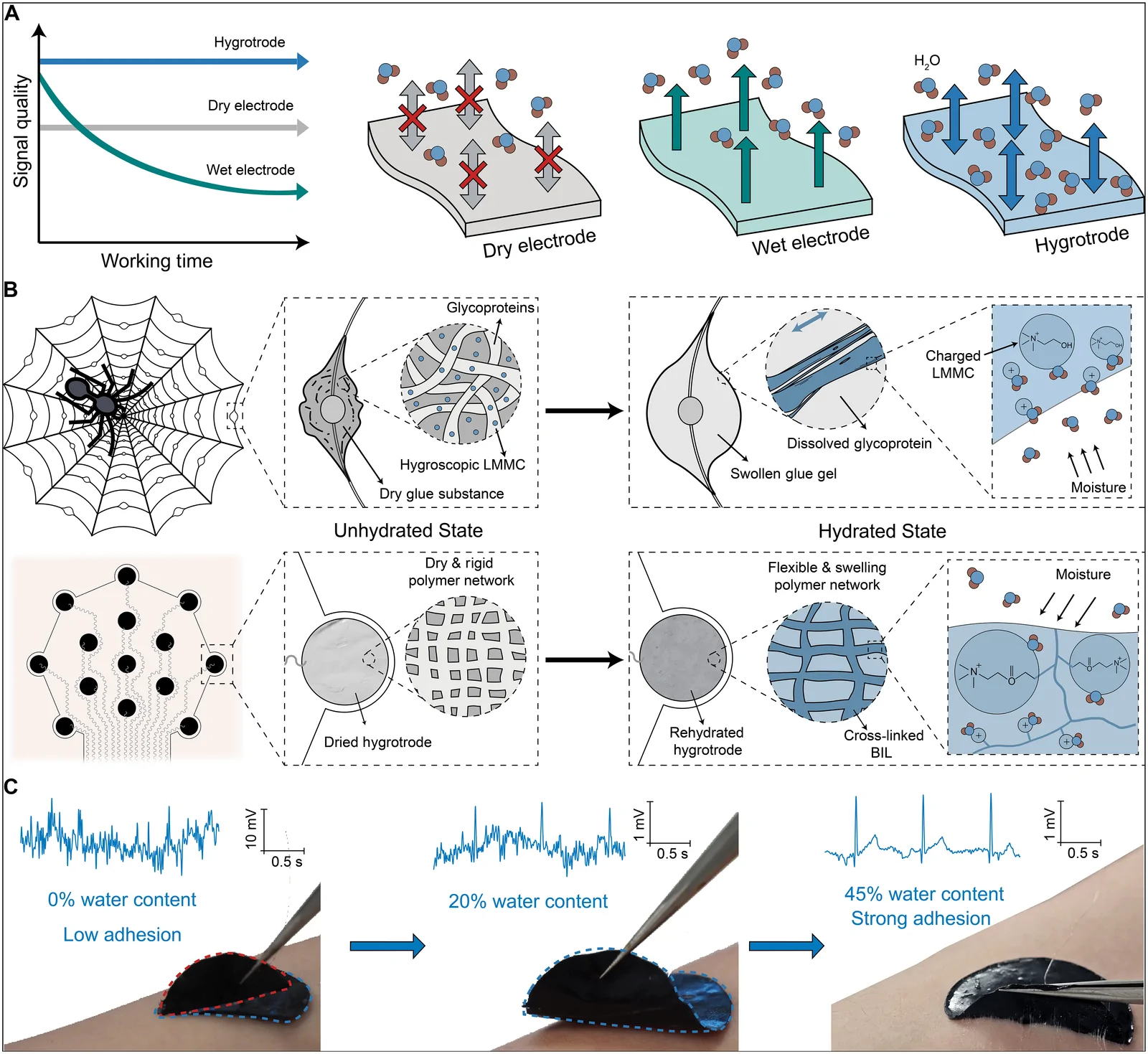
Schematics of bio-inspired hygrotrode. (**A**) Working time and signal quality comparison of metal/carbon-based dry electrode, hydrogel-based wet electrode and hygrotrode, highlighting its advantages of bidirectional water exchange, chronic usability, and high-quality signal acquisition. (**B**) Hygrotrode inspired by aggregate glue droplet in spider web, in which water content is modulated by hygroscopic charged molecules such as low–molecular weight compounds (LMMCs) and cross-linkable biocompatible ionic liquid (BIL). (**C**) Rehydration process (0, 0.5, and 6 hours, refers to dehydrated, partially rehydrated, and fully rehydrated state of hygrotrode) increases water content, adhesion, and signal quality.

An ideal chronic bioelectronic interface should combine low initial impedance of hydrogel-based wet electrode and chronically stable impedance of metal- or carbon-based dry electrode and thereby enable high-quality signal acquisition over prolonged period (Fig. 1A). Hygrotrode shows exceptional and stable electrochemical performance, as well as high water permeability, which is inspired by spider silk with ability to adapt its water content across varying humidity conditions. Low–molecular weight compounds (LMMCs) within spider web dragline, which are mostly positively charged choline-based molecules, tend to form electrostatic interaction with water molecules, facilitating strong water sorption from ambient moisture (*16*). The adsorbed water accelerates the solvation of glycoproteins, thereby increasing the viscosity and adhesion of spider silk (*17*, *18*). By introducing choline-based biocompatible ionic liquid {BIL; [2-(acryloyloxy)ethyl]trimethylammonium chloride}, hygrotrode similarly shows strong water sorption from ambient air and maintains high water content over prolonged duration (Fig. 1B). Meanwhile, elevated water content, together with the dual electronic-ionic conductivity of conducting polymer (CP) network in hygrotrode, offers an ultralow impedance electrochemical interface. In addition, the 3D charged network modulates the water states within polymeric matrix, providing high water permeability during extended wear. Immediately after hygrotrode was taken out of dry box, it shows poor adhesion strength and high noise level. When absorbed ∼45% water from ambient air, hygrotrode shows strong adhesive strength and good SNR, highlighting its unique ambient-moisture-dependent hygroscopic property (Fig. 1C).

### Hygroscopicity influence on electrochemical and mechanical properties

**Fig. 2.**
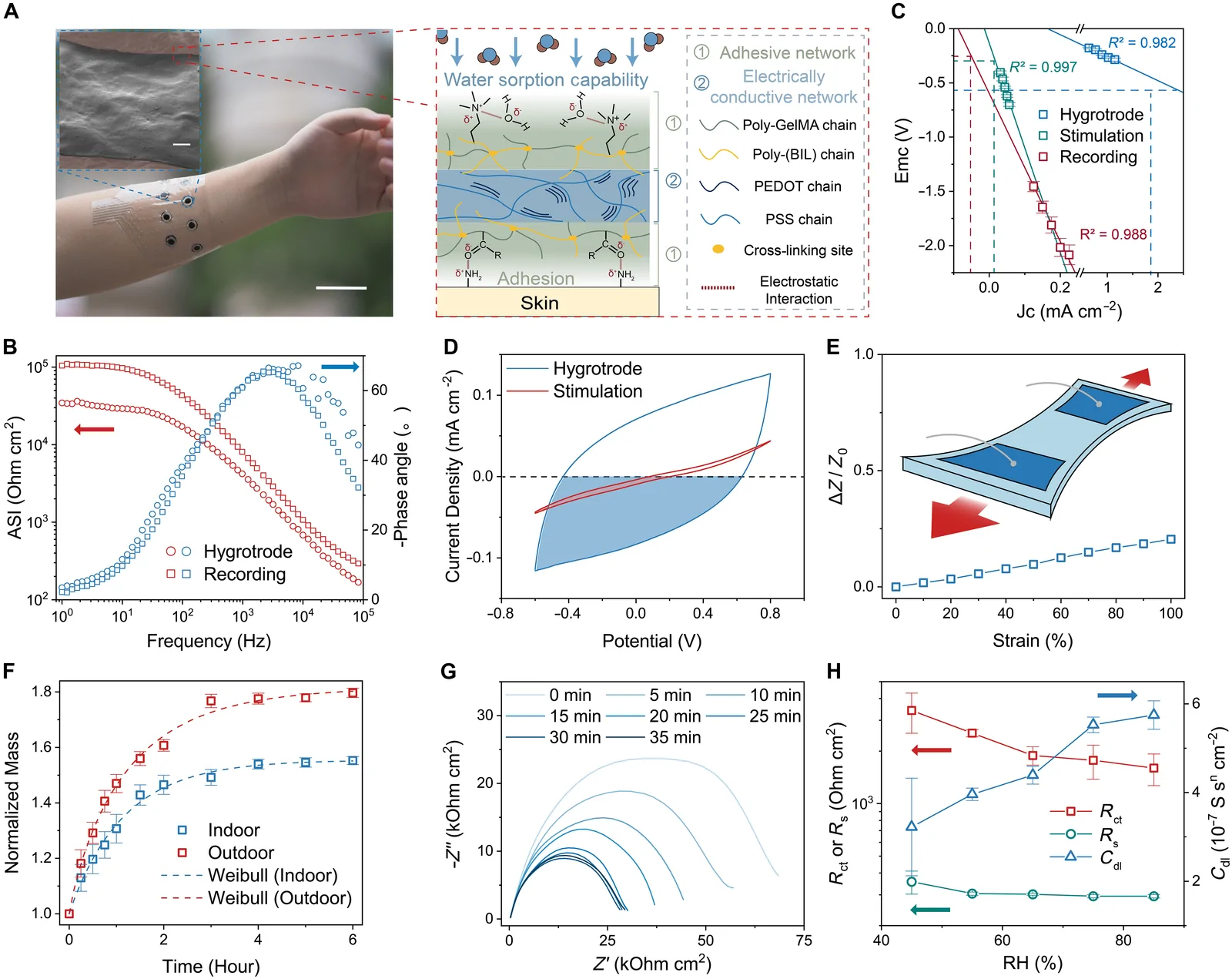
Electrochemical and mechanical performance of hygrotrode. (**A**) Photograph of hygrotrode array placed on human arm and illustration of its bilayer network structure. Inset: SEM image of hygrotrode conformed on a skin replica (scale bar, 100 μm). (**B**) ASI spectra and phase angle versus frequency curves of hygrotrode and Ag/AgCl-based wet recording electrode. (**C**) Calibration lines between maximum cathodally electrochemical potential excursion ($E_{\text{mc}}$) and cathodal phase current magnitude of different electrodes under voltage transients, with cathodic current density limit ($J_{c,\text{limit}}$) defined as current density at cathodic water electrolysis voltage for cathodic charge injection capacity (CIC_C_) calculation. Notably, negative $J_{c,\text{limit}}$ of wet recording electrode proves its infeasibility for stimulation application. (**D**) Cyclic voltammetry (CV) spectra of hygrotrode and carbon-based wet stimulation electrode. (**E**) Change of hygrotrode-skin interface impedance under strain (inset: experimental illustration showing two hygrotrodes placed on a stretchable ionic conductor). (**F**) Rehydration curve of hygrotrodes in indoor and outdoor environment, fitted by Weibull model. (**G**) Variation of Nyquist plot at hygrotrode-skin interface during in situ rehydration process. (**H**) Change of electrolyte resistance (*R*_s_), double-layer capacitance (*C*_dl_), and charge transfer resistance (*R*_ct_) at different relative humidity (RH).

We designed an interpenetrating bilayer network composed of an adhesive gelatin methacryloyl (GelMA) matrix, an electronically conductive PEDOT:PSS network, and a multifunctional BIL (fig. S1). At the molecular level, BIL plays three synergistic roles within this architecture. First, BIL serves as a secondary dopant and plasticizer for poly(3,4-ethylenedioxythiophene) polystyrene sulfonate (PEDOT:PSS), where the quaternary ammonium cations electrostatically interact with PSS chains, driving phase reorganization toward PEDOT-rich domains and thereby improving electronic conductivity and mechanical compliance under strain. Second, BIL is covalently cross-linked with the GelMA network, which not only reinforces interfacial adhesion to skin but also establishes a stabilized ionically conductive pathway within the hydrogel matrix. Third, the positively charged quaternary ammonium groups and associated chloride ions of BIL exhibit strong electrostatic interactions with water molecules, endowing the network with strong hygroscopicity and enabling rapid and reversible water sorption (Fig. 2A). The electrochemical performance and adhesion are mainly determined by BIL/CP layer and BIL/GelMA polymer network respectively, and were optimized sequentially to achieve highest adhesion energy (∼367.5 J m^−2^ when BIL/GelMA is 1:3) and lowest ASI (∼29.2 kohm cm^2^ at 10 Hz when BIL/CP is 4:1) (Fig. 2A, note S1, and figs. S2 and S3). Compared to Ag/AgCl-based wet recording electrode (∼97.0 kohm cm^2^ at 10 Hz), hygrotrode has ∼2.3 times lower ASI (∼29.2 kohm cm^2^ at 10 Hz), which can be ascribed to its dual electronic-ionic conductivity and relatively low charge transfer resistance (10.9 versus 73.3 kohms cm^2^) (Fig. 2B, fig. S4, and table S1).

To demonstrate its potential as multimodal electrode, stimulation performance of hygrotrode was first evaluated. Compared with carbon-based wet stimulation electrode, hygrotrode showed high cathodic current density (*J*_C_, 2.5 versus 0.02 mA cm^−2^; Fig. 2C) and cathodic charge injection capacity (CIC_C_, 1.2 × 10^−3^ mC cm^−2^ versus 1.2 × 10^−5^ mC cm^−2^) (fig. S5). Cyclic voltammetry (CV) test also revealed higher cathodic charge storage capacity (CSC_C_) for hygrotrode compared to commercial carbon-based wet stimulation electrode (5.1 versus 0.2 mC cm^−2^) (Fig. 2D). High CIC_C_ and CSC_C_ of hygrotrode suggest its capability to efficiently deliver transcutaneous electrical stimulation therapy within a safe voltage range. This can be attributed to hygrotrode’s high ratio of electrochemical surface area (ESA) to geometric surface area (GSA), which gives a higher double-layer capacitance (*C*_dl_) compared with that of wet stimulation electrode (2.1 versus 1.0 × 10^−7^ S s^n^ cm^−2^) (fig. S6 and table S1).

Desirable electrode-skin interface requires high stretchability and conformability. Owing to the plasticization effect of BIL, hygrotrode exhibited low electrode-skin interfacial impedance change of 20.4% at 100% strain and stable performance under cyclic strain (Fig. 2E and figs. S7 and S8). The strain-tolerant electrochemical properties of hygrotrode allow continuous and reliable signal acquisition under stress. The excellent conformability observed on the curvilinear skin surface (Fig. 2A) is attributed to sol-hygrotrode gradually filling the microscale skin wrinkles during a normothermic sol-gel transition at 34.1°C (fig. S9).

Hygrotrode can absorb ∼70% of its dry mass within 3 hours in outdoor environment (relative humidity, RH ∼70%) with fast rehydration rate (time constant ∼0.25 hours while placed on human skin), and its hygroscopicity remained stable after repetitive drying-rehydration cycles (Fig. 2F and figs. S10 and S11). The high hygroscopicity is attributed to the quaternary ammonium groups in the superabsorbent BIL, which interact strongly with water molecules through electrostatic binding (*19*, *20*). Component contribution analysis revealed that BIL significantly enhances water sorption content when added to CP and GelMA, and largely improves rehydration rate when compared to other hygroscopic materials, such as inorganic salt lithium chloride (LiCl) and uncross-linkable ionic liquid choline chloride (ChCl) (fig. S12). This underscores the potential of hygrotrode to rapidly rehydrate from a dry state.

Next, we investigated the effect of hygroscopicity on electrochemical properties. During rehydration, we observed that impedance of hygrotrode-skin interface gradually decreased (Fig. 2G and fig. S13). This implies improved signal quality. To study the hygroscopicity mechanism, the water content of hygrotrode was precisely controlled by changing RH (fig. S14), and its electrochemical impedance spectroscopy (EIS) was analyzed. As RH increased from 45 to 85%, the electrolyte resistance (*R*_s_) and charge-transfer resistance (*R*_ct_) decreased, while the double-layer capacitance (*C*_dl_) increased (Fig. 2H). This is possibly due to hydration of stratum corneum barrier, an increase of charge carrier mobility (*21*) and rehydration-induced expansion of the ESA (fig. S15). Despite reduced hygroscopicity at lower ambient RH, the skin-electrode microclimate maintains the interfacial humidity to levels higher than the environment (*22*–*25*). Consequently, the hygrotrode preserves stable ECG signal quality at an ambient RH of 50%, while only moderate degradation is observed as RH decreases to 30% (fig. S16).

### Hygroscopicity and water permeability for chronic bio-interface

**Fig. 3.**
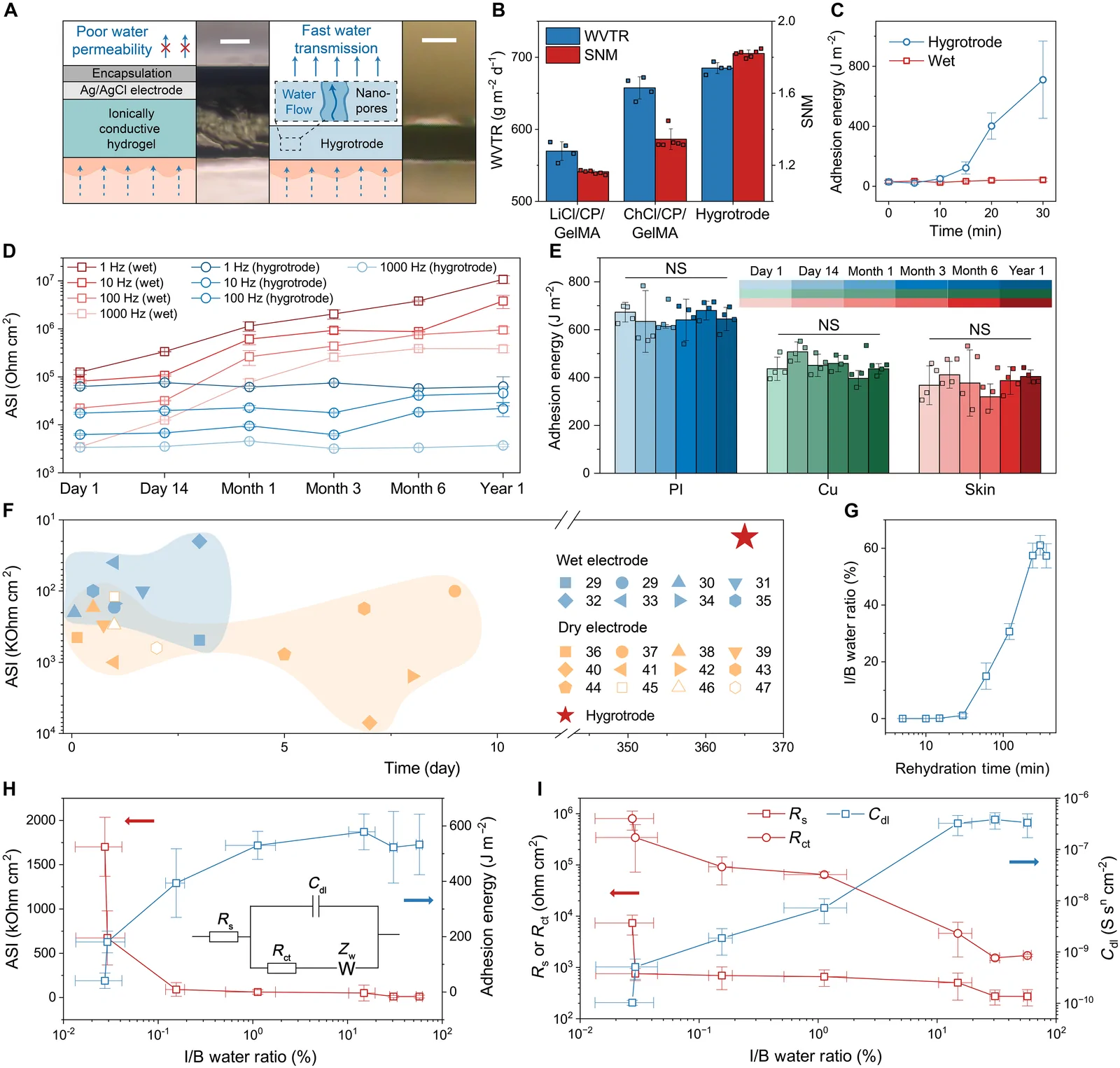
Hygroscopicity and water vapor permeability ensure chronic usability of hygrotrode. (**A**) Schematic illustration and image of wet electrode (left, image scale bar: 10 μm) and hygrotrode (right, image scale bar: 5 μm). (**B**) Water vapor transmission rate (WVTR, *T*: 37°C; RH: 40%) and saturated normalized mass (SNM) of different hygroscopic material-based trinary-component system. (**C**) Adhesion energy change of wet electrode and hygrotrode on heavily sweaty skin. (**D**) Area-specific impedance (ASI) change in long-term period (*n* = 4). (**E**) Adhesion energy change in long-term period (*n* = 4). (**F**) ASI versus monitoring time showing that hygrotrode keeps low ASI (at 10 Hz) over a prolonged period, greatly outperforming literature-reported dry and wet electrode (table S2). (**G**) I/B water ratio versus rehydration time. (**H**) ASI at 10 Hz and adhesion energy change with I/B water ratio (inset: equivalent circuit for Randle’s model fitting). (**I**) *C*_dl_, *R*_s_, and *R*_ct_ change with I/B water ratio.

Permeability is a key factor for long-term wearable devices (*26*–*28*). However, wet recording electrodes (hereafter referred to as “wet electrodes”) are typically encapsulated to decelerate electrolyte loss and covered by adhesive foam for adhesion improvement (Fig. 3A). These encapsulations with poor water permeability cause sweat accumulation, leading to degradation of signal quality and interfacial adhesion (*29*, *30*). Conventional hygroscopic adhesives also often show poor water permeability since strong interaction with water molecules largely increases evaporation energy. Compared to LiCl-based and ChCl-based hygroscopic systems, hygrotrode not only achieved highest saturated normalized mass (SNM) of ∼1.8, but also showed highest water vapor transmission rate (WVTR) of ∼680.4 g m^−2^ day^−1^ (Fig. 3B). This outperformed the wet electrode and typical substrate materials including polyimide, polydimethylsiloxane and polyurethane, and the WVTR of hygrotrode was comparable to the moisture evaporation rate from an unsealed bottle (fig. S17). In addition, highly hygroscopic nature of hygrotrode eliminates the need for encapsulation for water preservation, thereby further improves WVTR (Fig. 3A). On heavily sweaty skin, hygrotrode rapidly regained its adhesion within 30 min, and no degradation of electrochemical properties was observed (Fig. 3C and fig. S18). This is because high water permeability facilitates fast water transport from skin to the environment and restores the hydrogen bonding between hygrotrode and skin (*31*, *32*).

Combining high hygroscopicity and water vapor permeability, hygrotrode offers long-term stable bio-interface. Following 1-year aging process, ASI at the wet electrode-skin interface increased by over one order of magnitude (8.1 × 10^4^ versus 3.8 × 10^6^ ohm cm^2^ at 10 Hz), while the change at hygrotrode-skin interface is much smaller (1.7 × 10^4^ versus 4.6 × 10^4^ ohm cm^2^ at 10 Hz) (Fig. 3D). The interfacial adhesion energy of hygrotrode on polyimide, Cu and skin had <10% change during the 1-year period (Fig. 3E), and low variation after multiple peeling-offs. We observed only minor changes in adhesion energy, toughness, and fatigue resistance after prolonged wear (fig. S19). In contrast, the interfacial adhesion energy of wet electrode decreased ∼96.7% after 2 weeks (fig. S20). Skin irritation, cytotoxicity, hydration level, and TEWL results consistently confirm no adverse effects on skin health following long-term wear (fig. S21). The unique hygroscopic property of hygrotrode allows both low ASI and ultralong monitoring time simultaneously, surpassing LiCl- and glycerol-based hygroscopic material and outmatching previously reported wet (*33*–*39*) and dry electrodes (*40*–*51*) (Fig. 3F, fig. S22, and table S2).

Underlying mechanism behind high water vapor permeability relates to adaptive transition of water state. In conventional hygroscopic materials, uncross-linked hygroscopic salt (such as LiCl) gradually solvates and forms homogeneous solution where water molecules are strongly bounded by coordinate bonds with metallic cations (named BW). In addition to BW, the cross-linked three-dimensional charged network in hygrotrode allows a large amount of intermediate water (IW) to be trapped but not strongly bound in matrix pores (fig. S20). Comparing to BW, IW requires less energy to be evaporated (*52*). Raman spectroscopy reveals the coexistence of different water states, free water (FW) and IW, in LiCl-based, ChCl-based and BIL-based hygroscopic systems (fig. S24) (*52*). Differential scanning calorimetry (DSC) also revealed that stronger interaction with water decreased intermediate/bound water ratio (I/B water ratio; fig. S25) (*53*). In contrast to existing strategies that rely on encapsulation or unanchored hygroscopic additives, often involving trade-offs in breathability and long-term stability (*54*, *55*), the hygrotrode achieves sustained low impedance and high signal fidelity through a molecularly integrated mixed ionic-electronic conduction framework (table S3) (*13*, *56*–*82*).

We further investigate how the water state, characterized by I/B water ratio, affects impedance and adhesion of hygrotrode. I/B ratio increased from 0.027 to 1.1% after 30 min and stabilized at ∼57.4% after 240 min (Fig. 3G). Adhesion increases as I/B ratio increases (Fig. 3H). This is because BW hydrates and unfolds polymer chains, enhancing conformability. On the other hand, ASI decreases with increasing I/B ratio, attributable to decrease in uncompensated resistance *R*_s_ and *R*_ct_ and increase in double-layer capacitance *C*_dl_ (Fig. 3, H and I). The decrease in *R*_s_ may result from moistening of stratum corneum, while decrease of *R*_ct_ and increase in *C*_dl_ can be ascribed to IW electrochemically bridging additional CP to the skin tissue (Fig. 3I). Notably, further experiments with fixed water content and varying I/B ratio suggest that *R*_s_ is dominated by the overall hydration level, whereas *R*_ct_ and *C*_dl_ are primarily affected by I/B ratio (fig. S26). Increasing I/B ratio provides a general strategy to optimize adhesion and electrochemical properties for hygroscopic conductive adhesives.

### High-quality biopotential acquisition and precise gesture recognition

**Fig. 4.**
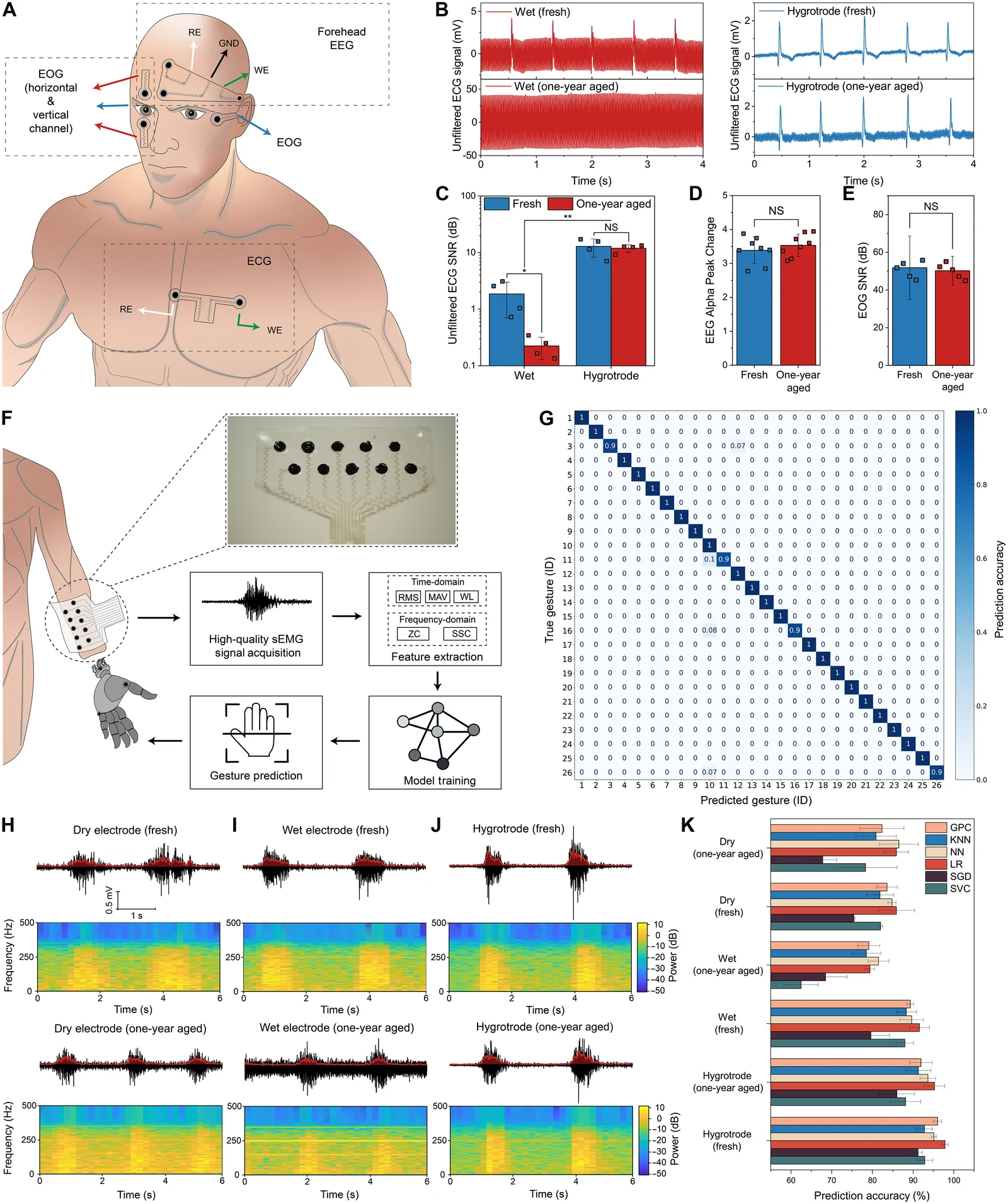
Biopotential monitoring applications of hygrotrode and demonstration in gesture recognition. (**A**) Illustration for different biopotential monitoring. (**B**) Unfiltered ECG data acquired by fresh and 1-year aged wet electrodes and hygrotrodes. (**C**) SNRs of ECG signals before and after 1 year aging for wet electrode and hygrotrode (*n* = 4). One-way ANOVA with Tukey post-hoc test: not significant (NS) *P* > 0.05, *P < 0.05, **P < 0.01, and ***P < 0.001 versus control. All error bars denote the SD. (**D**) SNR comparison of EOG data acquired by fresh and 1-year aged hygrotrode patches (*n* = 5). (**E**) Alpha peak-to-noise ratio comparison of EEG data acquired by fresh and 1-year aged hygrotrode patches (*n* = 8). (**F**) Schematic of gesture recognition process. (**G**) Confusion matrix of optimized model for full dataset recognition (prosthesis control and ASL gestures). (**H** to **J**) sEMG signals and its spectrogram acquired by fresh (upper) and 1-year aged (bottom) dry electrodes (H), fresh (upper) and 1-year aged (bottom) wet electrodes (I), and fresh (upper) and 1-year aged (bottom) hygrotrodes (J). (**K**) Prediction accuracy comparison of six models among fresh and 1-year aged electrodes (*n* = 4).

Hygrotrode array gave high-fidelity signal and chronically stable performance for various biopotential recording applications (Fig. 4A and fig. S27). Filtered and unfiltered ECG signals acquired by hygrotrode exhibited ∼7.8 and ∼11 dB higher SNR compared to those collected by wet electrode, as evidenced by spectrograms (Fig. 4B and fig. S28). SNR change of hygrotrode-acquired ECG signals was negligible after 1-year aging compared to wet electrode-acquired signals (∼0.055 versus ∼4.0 dB for filtered signals) (Fig. 4C and fig. S29). The two-channel (horizontal and vertical, Fig. 4A) hygrotrode patch successfully captured electrooculogram (EOG) signals generated by unidirectional eyeball movement, blinking, and eyeball rotation (fig. S30), with no obvious SNR decline observed after 1-year aging in ambient environment (50.1 versus 50.8 dB, Fig. 4D). In forehead electroencephalogram (EEG) application, hygrotrode successfully captured stable alpha waves with high SNR and power density during eye-open and eye-close activities (fig. S31), and no obvious decline in the alpha wave peak was observed even after 1-year aging (Fig. 4E).

Next, we demonstrate hygrotrode as a chronically stable human-machine interface for gesture recognition. A 10-channel hygrotrode patch (Fig. 4F) can decode 26 gestures from prosthesis control gestures (group I) and American Sign Language (ASL) gestures (group II) through recorded surface electromyography (sEMG) (fig. S32 and table S4). Well-resolved recording of muscle activity dynamics, as revealed by the peri-stimulus time histogram (PSTH), facilitated distinct clustering across various data visualization tools and achieved 100% accuracy in recognizing 26 gestures (Fig. 4G and figs. S33 to S37).

To demonstrate its feasibility for long-term use, we evaluated the performance of the hygrotrode patch during prolonged wear and after 1 year of aging. During 90-min exercise and 14-day continuous monitoring, the hygrotrode exhibited stable overall performance which indicates reliable long-term performance (fig. S38). By applying dimensionality reduction techniques, we identified robust clusters with high prediction accuracy of ∼100% for each gesture group individually after hyperparameter optimization (figs. S39 and S40). In contrast to metallic dry electrode, which exhibited relatively low SNR (5.9 versus 6.0 dB) and prediction accuracy (82.4% versus 81.8%), and wet electrode, which showed a significant decrease in SNR (9.2 versus 2.1 dB) and prediction accuracy (87.8% versus 75.5%), hygrotrode preserved its high SNR (9.8 versus 9.4 dB) and prediction accuracy (94.3% versus 91.0%) after 1 year of aging (Fig. 4, H to K, and figs. S41 and S42).

## DISCUSSION

In summary, our bio-inspired electronically conductive hygroscopic adhesive (hygrotrode) serves as a high-fidelity, low-impedance bioelectronic interface designed for long-term biopotential monitoring and electrical stimulation. By creating an interpenetrating 3D polymeric network of BIL, CP, and GelMA, low impedance, high charge storage and injection capacity, and strong interfacial adhesion are simultaneously integrated into the hygrotrode platform, meanwhile ensuring its stretchability and conformability. Hygroscopic BIL plays a crucial role in maintaining a balance between water sorption and volatilization through strong electrostatic interactions with water, thereby preserving the electrochemical and mechanical properties of hygrotrode for up to 1 year. We found that establishment of cross-linked charged network regulated the water state within the matrix by modulating I/B ratio. This approach endows hygrotrode with high water vapor permeability and self-adaptive on-skin humidity management, enabling rapid recovery from dry or sweaty states to a normal condition. Our investigation into how water states influence adhesive and electrochemical properties not only provides valuable insights for optimizing skin adhesives but also presents a broader strategy for designing next-generation bioelectronic interfaces. Unlike conventional wet electrodes, the hygrotrode patch maintained a stable sEMG SNR for over 1 year, highlighting its potential for long-term electrophysiological applications, including prosthesis control and sign-language translation. Although further improvements are needed in properties such as toughness, fatigue resistance, and tear resistance, we envision hygrotrode as a chronically stable, low-impedance interface for skin-adherent devices, with the potential to substantially extend the lifespan of wearable medical devices and human-machine interfaces.

## MATERIALS AND METHODS

### Materials

PEDOT:PSS aqueous solution (Clevios PH 1000) was supplied by Heraeus. Biocompatible ionic liquid (BIL) [2-(acryloyloxy)ethyl]trimethylammonium chloride solution, ammonium persulfate (APS), *N*,*N*′-Methylenebisacrylamide (MBAA), gelatin (from cold water fish skin), methacrylic anhydride (MA), phosphate-buffered saline (PBS) solution, sodium chloride (NaCl), lithium chloride (LiCl), glycerol, choline chloride (ChCl), N,N-Dimethylacetamide (DMAc) and dextran from *Leuconostoc* spp. (molecular weight of 100 k Da) were purchased from Sigma-Aldrich. Stretchable silicone rubber Ecoflex 00-20 was purchased from Smooth-On. Highly moisture-permeable and stretchable thermoplastic polyurethane (TPU) SP-80A was supplied by Lubrizol. Stretchable silver ink (DM-SIP-3060S) and flexible silver epoxy (DM-SAS-10030ST) were purchased from Dycotec Materials. Wet electrode for electrophysiology monitoring (2560) and double-side medical transfer adhesives (4075) were obtained from 3M.

### Synthesis of GelMA

GelMA was synthesized according to a previously reported protocol (*83*). In brief, 5 g of gelatin was dissolved in 50 ml of PBS solution that was preheated to 50°C. With stirring at 50°C for 2 hours, MA was then added dropwise to the gelatin solution until reaching 0.25, 1.25, or 20 v/v% of final solution volume, for low, medium, high methacrylation degree, respectively. After being diluted with 200 ml of PBS solution to terminate the reaction, the solution was dialyzed in deionized (DI) water using 10-kDa dialysis tube for a week. Last, GelMA with different methacrylation degrees were obtained after 7-day lyophilization and stored at 4°C until further use.

### Preparation of hygrotrode and device fabrication

BIL and CP (PEDOT:PSS with a defined molar ratio) were mixed and stirred for 20 min. The mixture was then poured into a Teflon mold and dried for 48 hours. Last, the obtained BIL/CP thin film was baked at 130°C for 30 min.

BIL and GelMA with a defined molar ratio, together with the cross-linker and initiator (MBAA and APS with a defined ratio) were added into 3.5% NaCl solution, followed by stirring for 1 hour. Then, CP/BIL film was soaked in the prepared solution overnight for complete interpenetration of GelMA and BIL monomers into the CP network. Last, BIL-GelMA network was polymerized by heating at 70°C for 24 hours to obtain hygrotrode. Before use, the hygrotrodes were soaked in DI water overnight and then allowed to equilibrate in air for 24 hours (fig. S43). Other hygroscopic component–based electrodes, including LiCl, glycerol, and ChCl, were prepared by replacing BIL with the same mass of the corresponding component.

Dextran aqueous solution (10 weight %) was spin-coated (1500 rpm, 20s) on an oxygen-plasma treated silicon wafer (150 W, 2 min) and heated at 130°C for 30 min to form the sacrificial layer, on which viscous TPU solution (12.5 w/v% in DMAc) was uniformly covered and annealed at 65°C overnight. The positions on the TPU substrate for adhering hygrotrodes were engraved by a laser engraver (Nova 7, iLaser), and silver ink (Dycotec) was subsequently screen-printed and heated at 100°C for 30 min to form stretchable conductive interconnects. Hygrotrodes were then fixed onto TPU substrate by silver epoxy, followed by curing at 120°C for 30 min. Next, 3M double-side adhesive was used to cover the interconnects and the circular edges of hygrotrodes to enhance adhesion with skin. Last, the electrode patch was released from the wafer by immersing in DI water.

^1^H nuclear magnetic resonance (NMR) spectroscopy was used for qualitative analysis of the soaking extracts. A BIL film specimen (0.5 cm by 1 cm) was immersed in 5 ml of D_2_O and incubated on a shaker with constant agitation for 24 hours. The film was then transferred to fresh D_2_O (5 ml) and incubated under identical conditions for an additional 5 days. ^1^H NMR spectra of the D_2_O extracts were acquired using a JEOL JNM-ECZL-500 NMR spectrometer. Thermogravimetric analysis (TGA) data was collected by Mettler Toledo TGA/DSC1. Samples were heated from room temperature to 70°C at a heating rate of 5°C/min and kept at 70°C for 1 hour. N_2_ flow rate is 40 mL/min at all stages.

### Electrical characterization

Conductivity, EIS and CV spectra were obtained by an electrochemical workstation (CHI 760D, Chenhua). Conductivity (σ) of hygrotrode was calculated from the following equation

$$\sigma =\frac{L}{R*A}=\frac{L}{R*W*d}$$

where *R* is the resistance, *L* is the length, *A* is the cross-sectional area, *W* is the width, and *d* is the thickness.

Three-electrode system was used for EIS test on skin, in the frequency range from 1 to 10,000 Hz under the driving voltage of 10 mV. After skin was prepared by 3M Trace Prep and alcohol swab, two hygrotrodes (as working and counter electrodes) were placed at the inner forearm (5 cm and 15 cm away from the wrist, respectively), and a commercial wet electrode (3M 2560) was placed at inner wrist as the reference. CV was obtained in the potential window −0.6 to 0.8 V at the scanning rate of 20 mV/s where two hygrotrodes were placed 5 cm apart on fresh and cleaned porcine skin. The cathodic charge storage capacity (CSC_C_) was calculated by the following equation

$${\text{CSC}}_{C}=\frac{\int J_{c}\mathrm{dV}}{V_{s}}$$

where the numerator is the integral of cathodic current density (shown as shadow in Fig. 3A), and “$V_{s}$” is the scan rate (V s^−1^).

Water electrolysis window of electrode was determined by CV test at constant scan rate of 20 mV/s. Potential window was expanded from −0.05 V to 0.05 V till resistive behavior or obvious redox peaks were observed (because faradic current leads to undesired ion injection, such as Ag^+^). Measurement of charge injection capacitance was performed on prepared porcine skin and Keithley 2636B sourcemeter was used to generate biphasic current pulse (cathodic-first) and monitor voltage transients, with a 500 μs duration for both cathodic ($t_{c}$) and anodic phases ($t_{a}$), and a 250 μs dwell time in between to determine the access voltage ($V_{a}$). Maximum cathodally electrochemical potential excursion ($E_{\text{mc}}$) is defined as the difference between access voltage and maximum negative voltage transients. It can be recorded as the instantaneous voltage 10 μs after the end of the cathodic phase, which correlates linearly with the cathodic current density ($J_{c}$). Cathodic current density limit ($J_{c,\text{limit}}$) was determined when $E_{\text{mc}}$ reached its cathodic limit of water electrolysis, and cathodic charge injection capacity (${\text{CIC}}_{C}$) was calculated by the following equation

$${\text{CIC}}_{C}=\frac{i_{c,\text{limit}}*t_{c}}{\text{GSA}}=J_{c,\text{limit}}*t_{c}$$

where GSA is the geometric surface area.

### Mechanical characterization

Mechanical property including adhesion, stretchability, toughness, and fatigue resistance were evaluated by Intron 5543 Tensile Meter. And adhesion energy was assessed by 90° peel-off test following previous report (*84*). Specifically, hygrotrode was adhered to the testing surface, and a vertical force was applied at the speed of 30 mm/min to peel the film off. The force versus displacement was recorded until complete detachment. To evaluate stretchability, the film was vertically stretched at the speed of 0.5% strain/s while the tensile force was continuously recorded and variation of resistance variation was monitored by Keithley 2636B sourcemeter. To evaluate toughness, hygrotrode specimens were tested at room temperature with a constant crosshead speed of 3 mm min^−1^ and an initial gauge length of 10 mm. Toughness was calculated as the area under the stress-strain curve up to fracture by numerical integration. To evaluate fatigue resistance, cyclic 180° peeling tests were performed under force-controlled conditions. An Instron testing machine applied cyclic peeling loads with a force amplitude $F_{a}$, which was set below the steady-state peeling force under monotonic loading. The tests were conducted for N cycles while continuously recording the interfacial crack length cas a function of cycle number. The applied energy release rate was calculated as $G=F_{a}/W$, where W is the sample width, and the interfacial crack propagation rate was determined as dc/dN. The tests were repeated at different force amplitudes, and the relationship between dc/dNand G was obtained. Fatigue resistance was quantified from the slope of the dc/dN − G curve, where a more negative slope indicates higher fatigue resistance.

Ecoflex 00-20 was used to replicate the microtexture structure of porcine skin to characterize conformability of hygrotrode, and hygrotrode was adhered to the replica and freeze-dried before being observed by SEM.

### Characterizations of the hygroscopic property

To investigate the water sorption capability of hygrotrode, it was fully dried in oven (70°C, 6 hours; fig. S44), and mass change was monitored with or without being adhered onto skin, in indoor (25°C, RH 40%) or outdoor (30°C, RH 70%) environment. Peleg and Weibull models were used to fit the experimental data using the following equations (*85*, *86*)

$$\text{Peleg Model}:M\left(t\right)=M_{0}+\frac{t}{a+\mathrm{bt}}$$

where M(t) is the instant normalized mass, $M_{0}$ is dry-basis mass, and a and b are model parameters;

$$\text{Weibull Model}:M\left(t\right)=M_{e}+\left(M_{0}-M_{e}\right)*\text{exp}\left[-{\left(\frac{t}{\beta }\right)}^{\alpha }\right]$$

where $M_{e}$ is the equilibrium mass normalized to the initial mass and α and β are model parameters.

The SNM is defined as the relative mass increase at equilibrium with respect to the dry mass, calculated as

$$\text{SNM}=\frac{m_{\text{eq}}-m_{\text{dry}}}{m_{\text{dry}}}$$

where “$m_{\text{dry}}$” is the mass of the fully dried hygrotrode and “$m_{\text{eq}}$” is the equilibrium mass under a given RH. Specifically, the equilibrium mass was obtained by continuously monitoring the sample mass under a fixed RH until the mass variation became negligible (<1% over 60 min).

In one cycle of repetitive drying/rehydrating test, hygrotrode film was dried at 70°C for 6 hours and then rehydrated in outdoor environment for 6 hours, and its normalized mass was measured and averaged from three samples. To measure the equilibrium mass of hygrotrode at different RH% values, freshly prepared hygrotrode films were fully dried, placed in humidity-controlled cabinet, weighted after the mass reached the steady value.

### Long-term impedance and adhesion test of hygrotrode

Both hygrotrode films and control samples were stored in an air-conditioned room at a temperature of 25°C and at a RH of 40%. The samples were stored in petri dishes and covered with perforated parafilm to minimize dust deposition and potential environmental contamination. The ASI and adhesion energy were tested at 7, 14, 30, 90, 180, and 365 days.

For intermittent long-term test, hygrotrodes used for long-term ASI and adhesion testing were worn intermittently on the forearm during daily work hours (from 9:30 a.m. to 5:30 p.m. on weekdays). EIS measurements were performed at 3:00 p.m. and adhesion tests at 4:00 p.m. each day. During wear, all routine activities were allowed, including indoor and outdoor activities and exercise, but water-related activities (e.g., swimming) were avoided. Outside of the wearing periods, the hygrotrodes were stored in petri dishes covered with perforated parafilm to minimize dust deposition and potential environmental contamination.

For continuous long-term test, two hygrotrodes were placed at a specific position on the chest (upper left thorax, approximately at the level of the third rib, 8 cm apart, and aligned across the sinus). All routine activities and exercise were permitted, and a protective cover (3M Tegaderm) was applied when showering; bathing or swimming was not allowed.

### Water vapor transmission test

Glass vials containing 4 g of DI water were used for the experiment. Tested films (25 or 50 μm thickness) were attached to the opening of a glass vial, while another vial was kept open for comparison. Following the protocol ASTM E96, a standard test method for WVTR, the samples were kept in a humidistat chamber at 25°C and 50% RH. To mimic the on-skin condition, the samples were stored in an incubator at 37°C and 40% RH. Vial weights were measured every 24 hours for 14 days. WVTR in g m^−2^ day^−1^ was calculated according to the following equation

$$\text{WVTR}=\frac{m_{1}-m_{2}}{S}$$

where $m_{1}$ and $m_{2}$ are the vial weight before and after measurement, and S is the opening area of the vial.

Skin hydration level and transepidermal water loss (TEWL) at the electrode–skin interface were measured using a skin hydration probe (Real Bubee) and a VapoMeter (DelfinTech), respectively. Before measurement, the skin was gently cleaned with 70% (v/v) ethanol and allowed to equilibrate to ambient conditions for 10 min. The test samples (2 cm by 2 cm) were applied to the forearm. Skin hydration level and TEWL were recorded before application, during wear and after removal. All measurements were conducted under identical environmental conditions to minimize variability.

### Biocompatibility evaluation

Skin irritation was evaluated in healthy volunteers following a patch-wearing protocol. Before testing, the skin was gently cleaned with 70% (v/v) ethanol (or mild soap and water) and allowed to air-dry. The test samples (2 cm by 2 cm) were applied to the forearm. Each subject wore the samples for 8 hours under normal daily activities (no intentional sweating), and the samples were removed thereafter. Digital photographs of the application sites were taken before application, during wear, and immediately after removal under identical conditions.

Cytotoxicity was evaluated using an extract-based assay. In brief, a hygrotrode specimen (1 cm by 1 cm) was immersed in 20 ml of Dulbecco’s modified Eagle’s medium (DMEM) supplemented with 10% fetal bovine serum and 1% penicillin-streptomycin, followed by incubation on a shaker for 24 hours. The resulting extract medium was filtered and collected for subsequent use. NIH-3T3 cells were cultured in 24-well plates with either the extract medium or fresh DMEM for 72 hours at 37°C under a 5% CO_2_ atmosphere. In parallel, cells were cultured in 96-well plates under the same conditions for fluorescence intensity quantification. After 72 hours, the culture medium was removed and the cells were washed once with PBS. A staining solution containing 2 μM calcein-AM and 8 μM propidium iodide (PI) in PBS was added, followed by incubation at 37°C for 15 min in the dark. After staining, the cells were washed with culture medium. Live/dead fluorescence images were immediately acquired using a confocal laser scanning microscope (LSM800, Carl Zeiss) (calcein-AM: λ_ex_/λ_em_ = 499/515 nm; PI: λ_ex_/λ_em_ = 535/617 nm). For cells cultured in 96-well plates, fluorescence intensity was measured using a BioTek Synergy H1 microplate reader using the same excitation/emission settings.

### Water state analysis

Raman measurements were performed spectrometer (Renishaw InVia Reflex Raman Spectrometer) equipped with a 514-nm excitation laser. All spectra were collected at room temperature under ambient conditions.

DSC measurements were carried out using a PerkinElmer DSC 4000 under a nitrogen atmosphere. The samples were heated from −60°C to 10°C at a constant heating rate of 10°C min^−1^.

The I/B water ratio is defined as the ratio of IW to BW within the tested material, as determined from DSC. Specifically, the total water content of each sample was precisely determined prior to DSC analysis. During the DSC heating scan, water melting between −1° and 1°C (accounting for instrumental error and thermal lag) was assigned to FW, water melting below −1°C was assigned to IW, and the remaining nonmelting water was considered BW (*87*). The I/B water ratio was then calculated as the mass ratio of IW to BW.

### Biopotential recording and gesture recognition

Biopotential signals (ECG, EOG, EEG, and sEMG) were acquired with hygrotrode patch by a wirelessly communicated amplifier (LiveAmp, Brain Products GmbH).

For ECG monitoring, a two-hygrotrode patch (1 cm diameter, 8 cm apart) was placed at the upper left-thorax (approximately at the level of 3rd rib) of a volunteer at rest state, crossing the sinus node where ECG signal was originated. ECG signal was acquired at the sampling rate of 1000 Hz and bandpass filtered (0.1 to 50 Hz). 3M RedDot electrode (1 cm diameter) was used for comparison.

A three-hygrotrode patch (placed at F_7_, TP_7_, and F_PZ_ position, for working, reference, and ground electrode, respectively) was used for EEG monitoring. Alpha wave during eye-close and eye-open conditions was recorded at the sampling rate of 1000 Hz and bandpass filtered (8 to 13 Hz).

For EOG monitoring, two single-hygrotrode patches were placed at the middle of forehead (at the *F*_pz_ node for EEG electrode placement) and 2 cm below the lower eyelids. EOG signal was sampled at the rate of 1000 Hz and bandpass filtered (0.5 to 30 Hz). SNR of all electrophysiological signal was calculated on the basis of following formula

$$\text{SNR}=10*{\text{log}}_{10}\left(\frac{P_{\text{signal}}}{P_{\text{noise}}}\right)$$

where $P_{\text{signal}}$ is average power of the signals and $P_{\text{noise}}$ is average power of the noise. They can be calculated based on following formula

$$P=\frac{1}{N}\sum_{i=1}^{N}n_{i}^{2}$$

where $n_{i}$ is value of datapoint, and N is the number of datapoints.

Motion artifact was induced by applying vertical external 50 kPa pressure onto volunteer’s arm and EMG deviations were recorded. The motion artifact value induced by pressure application and removal were calculated based on following equation

$$\begin{array}{c} {\text{SNR}}_{\text{MA}}=\frac{1}{2}\left({\text{SNR}}_{\text{PA}}+{\text{SNR}}_{\text{PR}}\right)=\frac{1}{2}\left(10*{\text{log}}_{10}\left(\frac{P_{\text{PA}}}{P_{\text{background}}}\right)\right.\\ \left.+10*{\text{log}}_{10}\left(\frac{P_{\text{PR}}}{P_{\text{background}}}\right)\right) \end{array}$$

where ${\text{SNR}}_{\text{MA}}$ is SNR of motion artifact, ${\text{SNR}}_{\text{PA}}$ is SNR of pressure application, ${\text{SNR}}_{\text{PR}}$ is SNR of pressure removal, $P_{\text{PA}}$ is average power of signals when pressure is applied, $P_{\text{PR}}$ is average power of signals when pressure is removed, $P_{\text{background}}$ is average power of background signals.

sEMG was recorded at the sampling rate of 1000 Hz and bandpass-filtered (10 to 300 Hz). A 10-hygrotrode patch was placed on the inner forearm of the volunteer (5 cm away from the wrist), and reference electrode (3M RedDot) was placed at the outer elbow. The gesture-recognition task was conducted on four subjects (two males and two females) for each electrode type, using a user-specific optimization strategy. To ensure consistency, a 10-fold repeated random sub-sampling validation (80/20 train/test split) was uniformly applied across all subjects and electrode variants to evaluate their signal acquisition performance. Both time-domain and frequency-domain features of sEMG signals were extracted for machine learning. The time-domain features included (*88*)

$$\text{RMS}\;\left(\text{root mean square}\right)=\sqrt{\frac{1}{N}\sum_{i=1}^{N}x_{i}^{2}}$$

$$\text{MAV}\;\left(\text{mean absolute value}\right)=\frac{1}{N}\sum_{i=1}^{N}|x_{i}|$$

$$\text{WL}\;\left(\text{waveform length}\right)=\sum_{i=1}^{N-1}|x_{i+1}-x_{i}|$$

The frequency-domain features included (*88*)

$$\text{ZC}\;\left(\text{zero}-\text{crossing}\right)=\sum_{i=1}^{N-1}\left[f\left(x_{i+1}\times x_{i}\right)\cap (|x_{i+1}-x_{i}|\geq \text{threshold}\right]$$

$$\text{SSC}\;\left(\text{slope}\;\text{sign}\;\text{change}\right)=\sum_{i=2}^{N-1}\left[f\left[\left(x_{i}-x_{i-1}\right)\times \left(x_{i}-x_{i+1}\right)\right]\right]$$

where

$$f\left(x\right)=\left\{\begin{array}{cc} 1, & \text{if}\;x\geq \text{threshold}\;\\ 0, & \text{otherwise}\; \end{array}\right.$$

Various algorithms (Andrew’s plot, *t*-distributed stochastic neighbor embedding, and uniform manifold approximation and projection) were used for dimension reduction and data visualization. Hyperparameters selected to optimize the linear regression model include: penalty, tolerance (“tol”), inverse of regularization strength (“C”), optimization algorithm (“solver”) and maximum number of iterations for convergence (“max_iter”) (Table 1). The searching for best parameters grid was powered by GridSearchCV (scikit-learn API 0.24.2) among a predefined grid matrix (table S5).

**Table 1.** Grid matrix for hyperparameter tuning.

Parameter	Values
penalty	“l1,” “l2,” “elasticnet,” “none”
Tol	0.00001, 0.0001, 0.001, 0.01, 0.1
C	0.01,0.1,1,10,100
solver	“lbfgs,” “sag,” “saga”
max_iter	1000, 5000, 10,000

SHAP analysis was supported by SHAP API 0.39.0. PSTH analysis was visualized by seaborn API 0.11.2. Specifically, spike was identified beyond a threshold of 0.2 mV, and the duration of the signal segment was set to be 100 μs.

## Supplementary Materials

### This PDF file includes:

Supplementary Text Figs. S1 to S44 Tables S1 to S5 References
