# Supplementary material for: Spiderweb-inspired electronically conductive hygroscopic adhesive for chronic bioelectronic interface
Source: Sci Adv. 2026 Aug 21;12(34):eaea3748. doi: 10.1126/sciadv.aea3748 (PMC13496177; doi:10.1126/sciadv.aea3748)
Supplement: Supplementary file 1 [file sciadv.aea3748_sm.pdf]

Science Advances
AAAS

Supplementary Materials for

Spiderweb-inspired electronically conductive hygroscopic adhesive for chronic bioelectronic interface

Lingxuan Kong *et al.*

Corresponding author: Yuxin Liu, lyx@nus.edu.sg; Peng Chen, ChenPeng@ntu.edu.sg

Sci. Adv. **12**, eaea3748 (2026)
DOI: 10.1126/sciadv.aea3748

This PDF file includes:

Supplementary Text
Figs. S1 to S44
Tables S1 to S5
References

SUPPLEMENTARY MATERIALS

Supplementary Note 1: optimization process

The electrochemical properties and adhesion are determined by BIL/CP and BIL/GelMA layer respectively. ASI of BIL/CP films with different ratio of BIL to CP were tested to determine the optimum BIL/CP recipe. Then Few parameters of BIL/GelMA layer must be determined: BIL/GelMA ratio, GelMA methacrylated degree and APS&MBAA content. ASI and adhesion energy (on three different substrates) of ECHA with different ratio of BIL to GeMA were utilized to determine the BIL/GelMA ratio. Adhesion energy of hygrotrode with different GelMA methacrylation degree and different APS&MBAA content was utilized to determine the optimum parameters. Determination of methacrylation degree was based on the free amino acid quantification method(*89*).

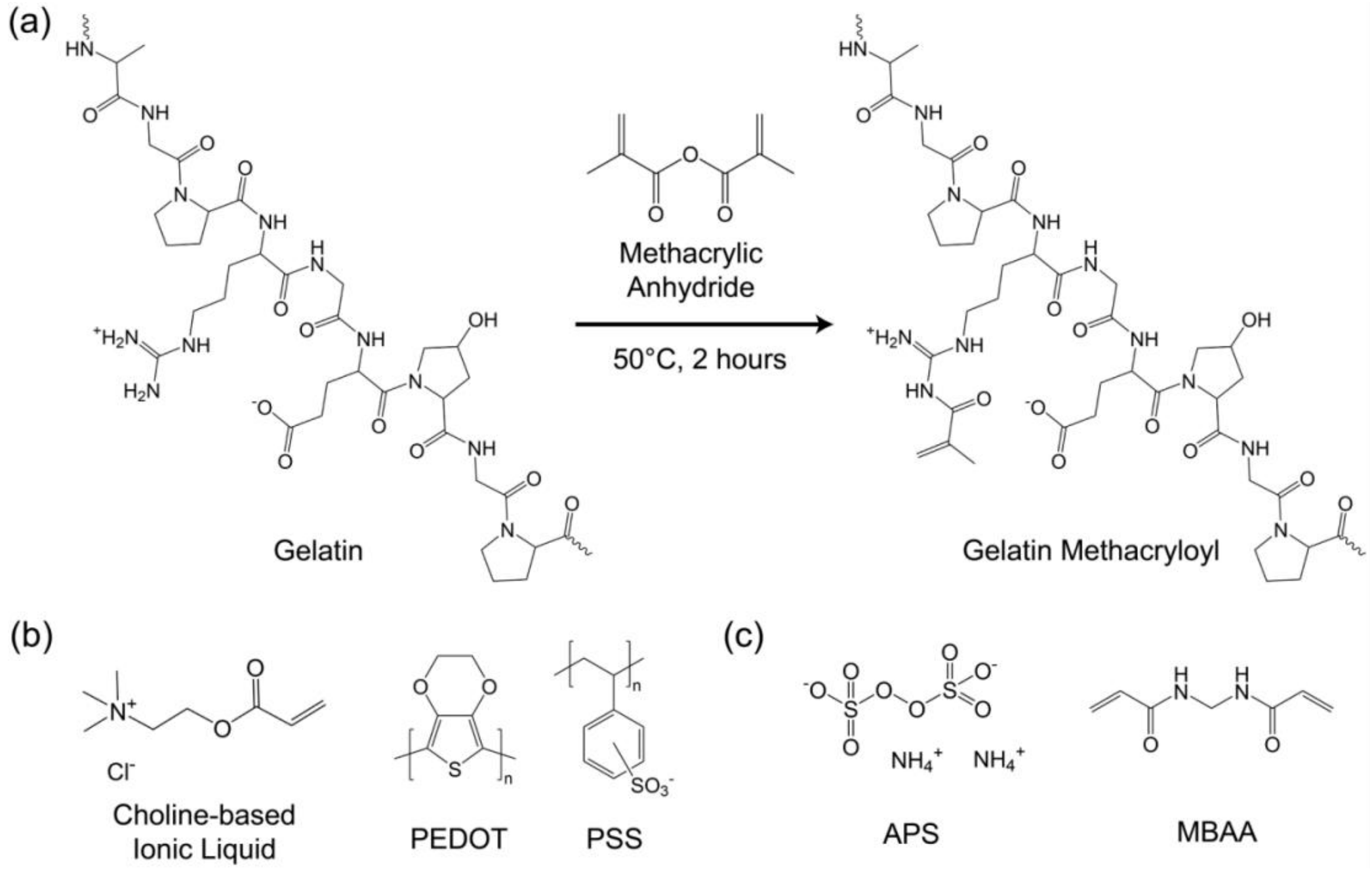

Supplementary Fig. 1. Chemical structures. (a) Methacrylation reaction formula of gelatin. (b) Chemical structures of BIL and PEDOT:PSS. (c) Chemical structures of APS & MBAA.

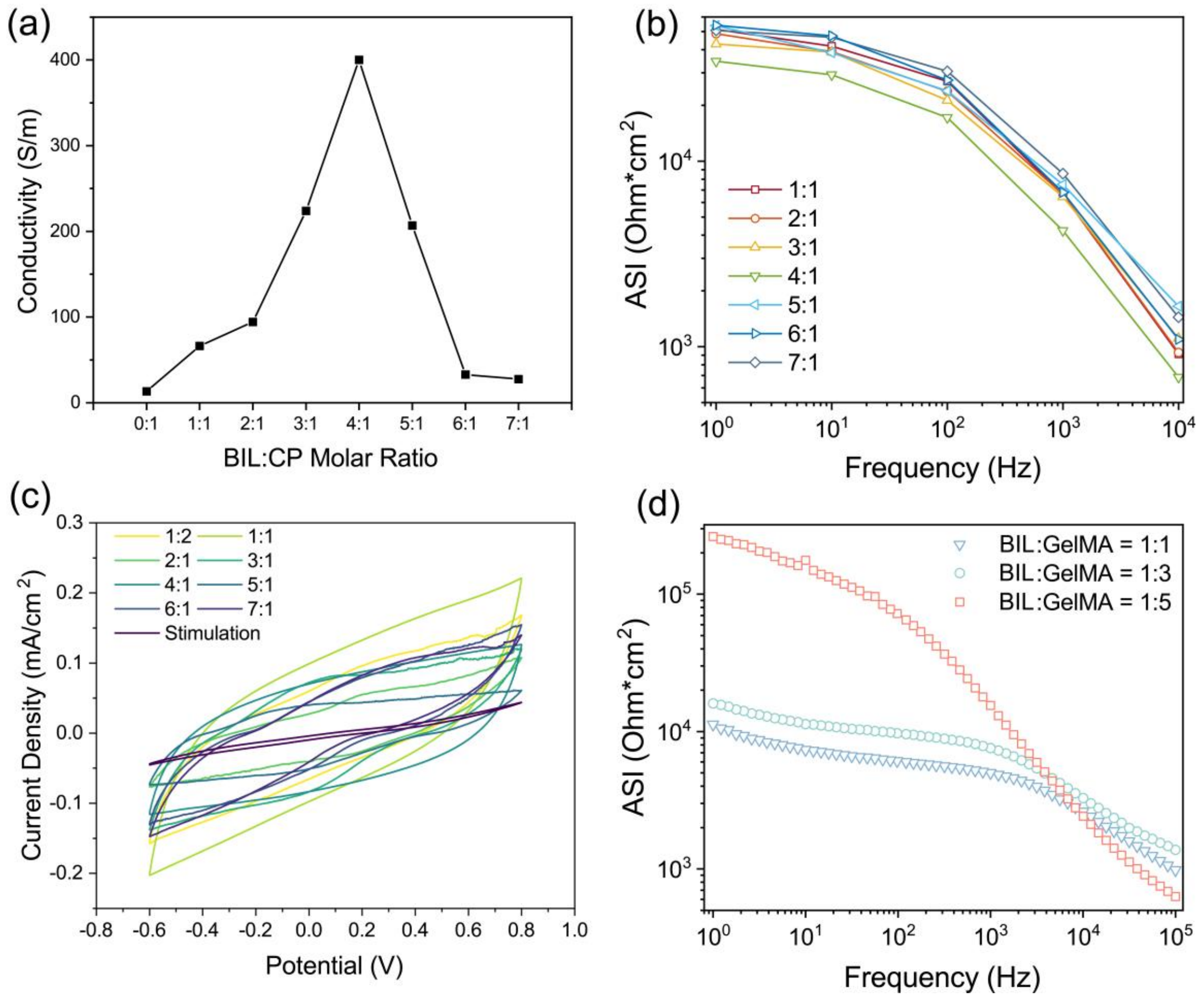

Supplementary Fig. 2. Optimization of electrochemical property. (a) Dependence of the conductivity of hygrotrode on the ratio of BIL to PEDOT:PSS. (b) Area specific impedance (ASI) spectra of films with different molar fraction of biocompatible ionic liquid (BIL). This correlation is attributed to the BIL-assisted de-doping of PEDOT from PSS chains to form PEDOT-rich domains. However, excess BIL increases viscosity leading to PEDOT:PSS aggregation. (c) Cyclic voltammetry spectra of different BIL/CP ratio-based hygrotrode and carbon-based wet stimulation electrode. (d) Effect of BIL:GelMA molar ratio on area-specific impedance. The ratio of BIL to conducting polymer (CP; named as BIL:CP ratio) of CP/BIL film is kept 4:1.

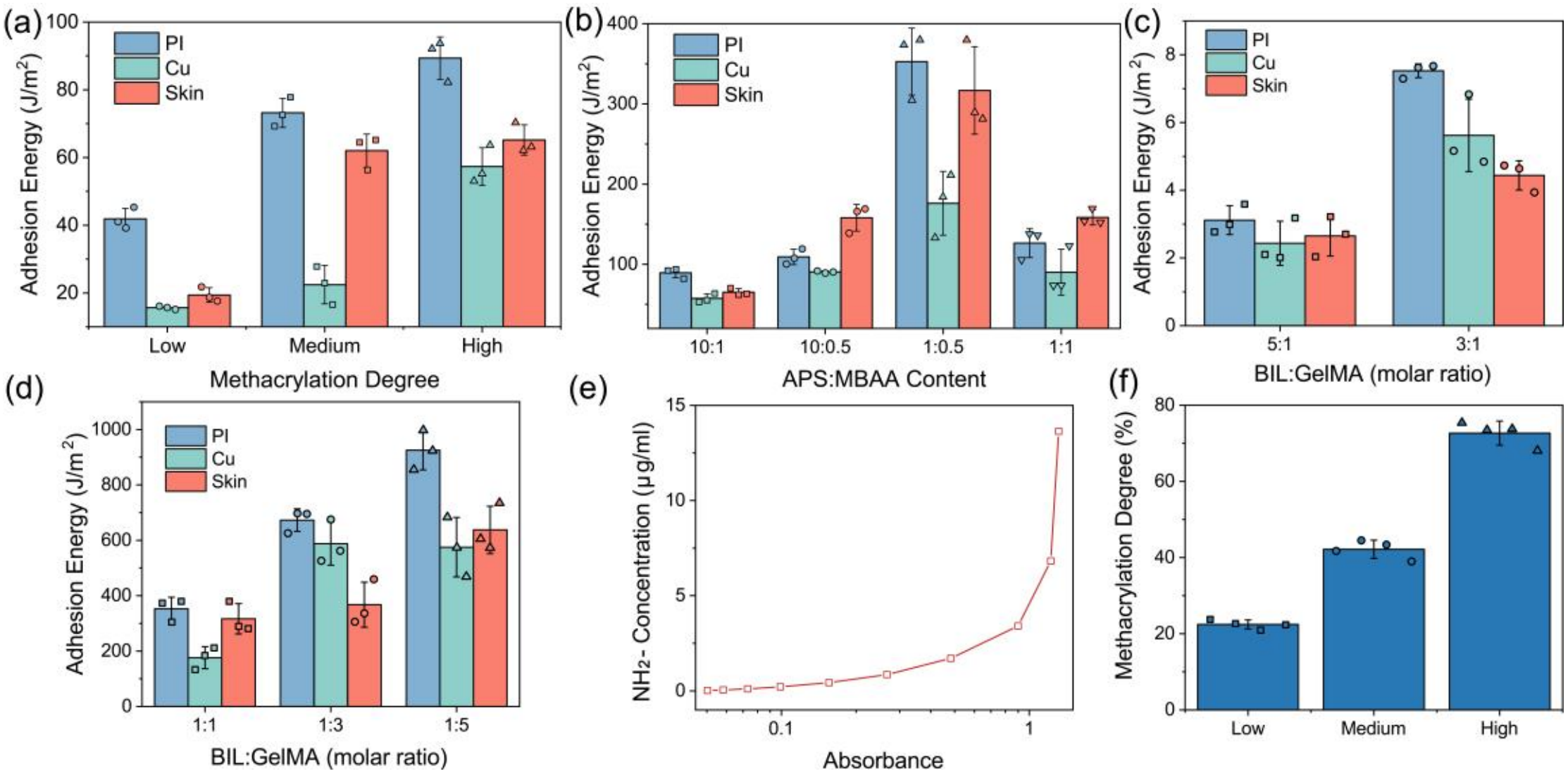

Supplementary Fig. 3. Optimization of adhesiveness. (a) Effect of GelMA's methacrylation degree on interfacial adhesion energy (on typical polymer (polyimide), metal substrate (copper) and skin). (b) Effect of APS:MBAA content (weight percentage over monomer weight) on interfacial adhesion energy. It is because ester groups added by methacrylation and low crosslinking density lead to higher interfacial energy(*90*). (c, d) Effect of BIL:GelMA molar ratio on interfacial adhesion

energy. (e) UV absorbance calibration line of free primary amine groups. (f) Methacrylation degree of GelMA-Low, GelMA-medium and GelMA-high (n = 4).

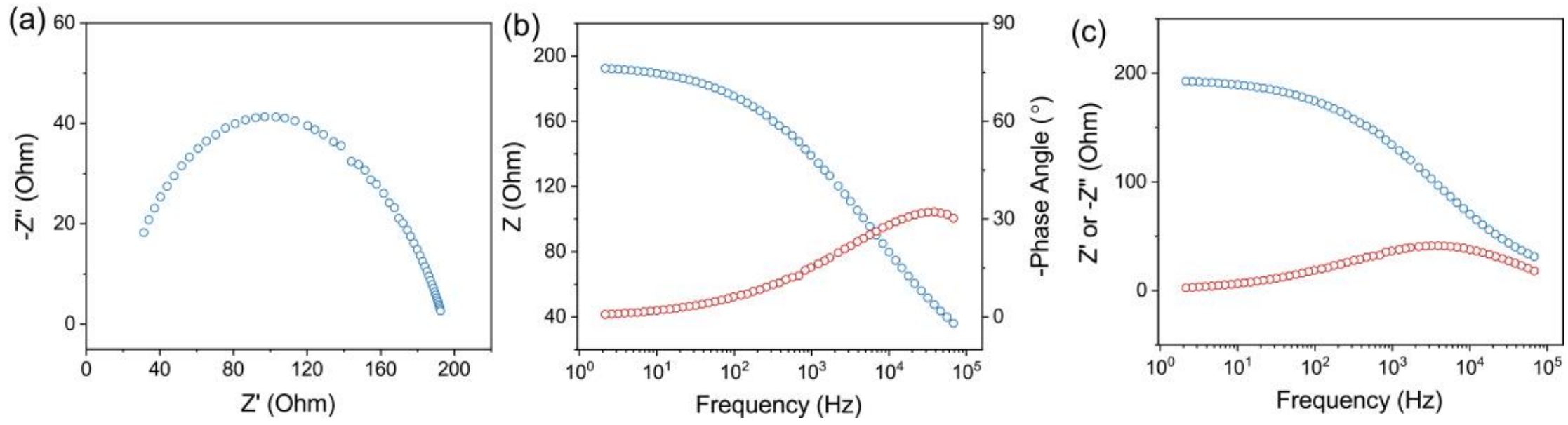

Supplementary Fig. 4. EIS of hygrotrode (experimental data: scatter; fitted data: bold line). (a) Nyquist plot of experimental and fitting data of hygrotrode. (b) Bode plot of experimental and fitting data of hygrotrode. (c) Real and imaginary part of impedance of hygrotrode.

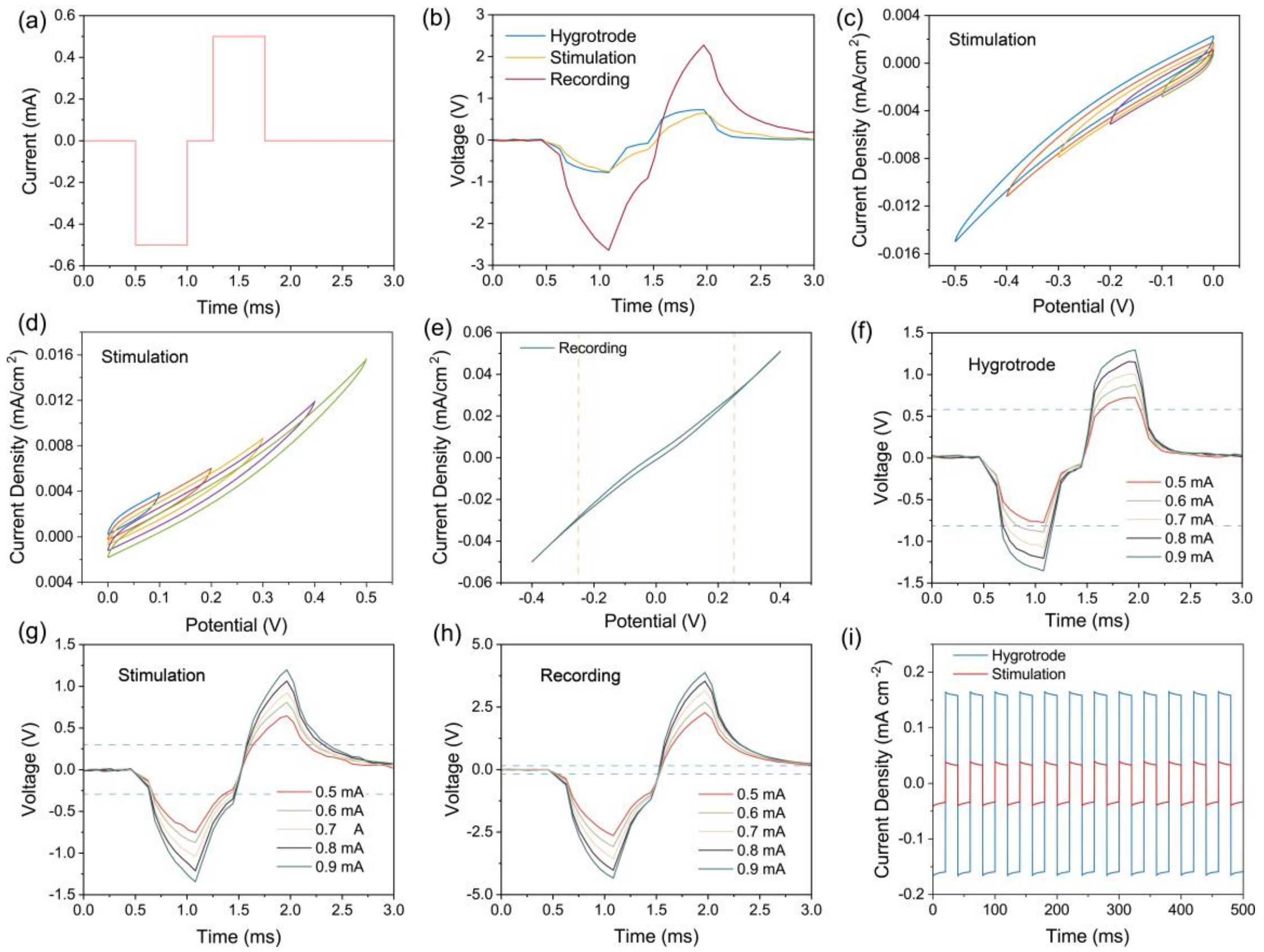

Supplementary Fig. 5. Stimulation characterizations. (a) Biphasic current pulse generated by hygrotrode, carbon-based wet stimulation electrode and Ag/AgCl-based wet recording electrode

(amplitude: 0.5 mA). Amplitude of cathodic and anodic current are equal, with equal phase duration of 500 μs ($t_c = t_a = 500\ \mu s$). A 250 μs dwell is employed between phases to measure the maximum cathodic electrochemical potential excursions (E_{mc}). (b) Voltage transients of hygrotrode (area: 0.785 cm^2), wet stimulation electrode (area: 16 cm^2) and wet recording electrode (area: 4 cm^2) in response to 0.5 mA biphasic current pulse. (c) CV spectrum shows the negative polarization below -0.3 V, proving that the cathodic limit of wet stimulation electrode is -0.3 V. (d) CV spectrum shows that positive polarization above 0.3V, proving that the anodic limit of wet stimulation electrode is 0.3V. (e) CV spectrum shows resistive behavior above 0.25 V and below -0.25 V, proving that the safe potential window of wet recording electrode is -0.25 ~ 0.25 V. (f) Voltage transients of hygrotrode in response to biphasic current pulse ranging from 0.5 to 0.9 mA. (g) Voltage transients of wet stimulation electrode in response to biphasic current pulse ranging from 0.5 to 0.9 mA. (h) Voltage transients of wet stimulation electrode in response to biphasic current pulse ranging from 0.5 to 0.9 mA. (i) Current density of hygrotrode and wet stimulation electrode, in response to 25Hz, ±0.5V biphasic voltage pulse.

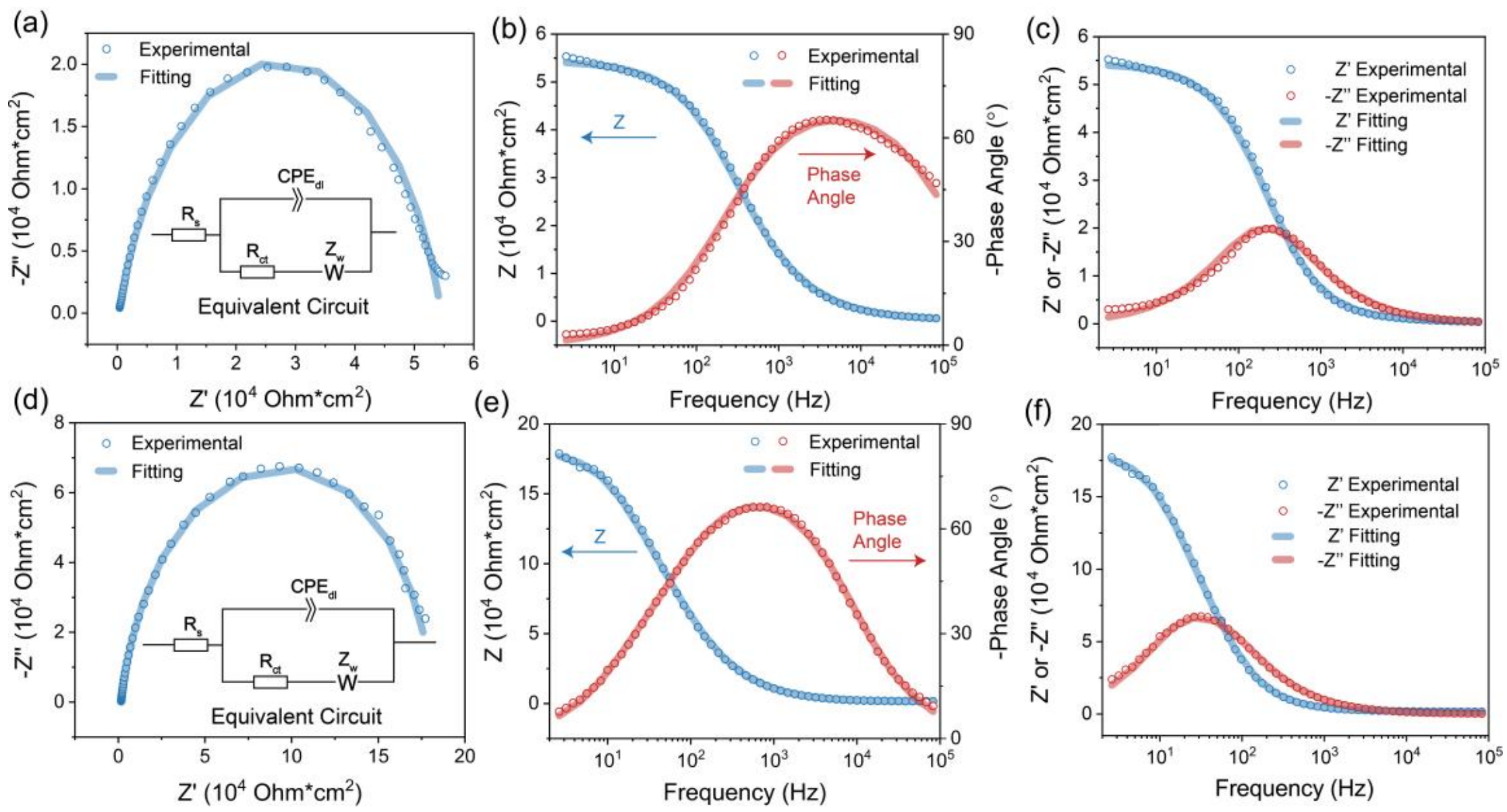

Supplementary Fig. 6. Randles circuit modelling. (a) Nyquist plot of experimental and fitting data of hygrotrode-skin interface (Inset: equivalent circuit for fitting). (b) Bode plot of experimental and fitting data of hygrotrode-skin interface. (c) Real and imaginary part of impedance of hygrotrode-skin interface. (d) Nyquist plot of experimental and fitting data of wet recording electrode-skin interface (Inset: equivalent circuit for fitting). (e) Bode plot of experimental and fitting data of wet recording electrode-skin interface. (f) Real and imaginary part of impedance of wet recording electrode-skin interface.

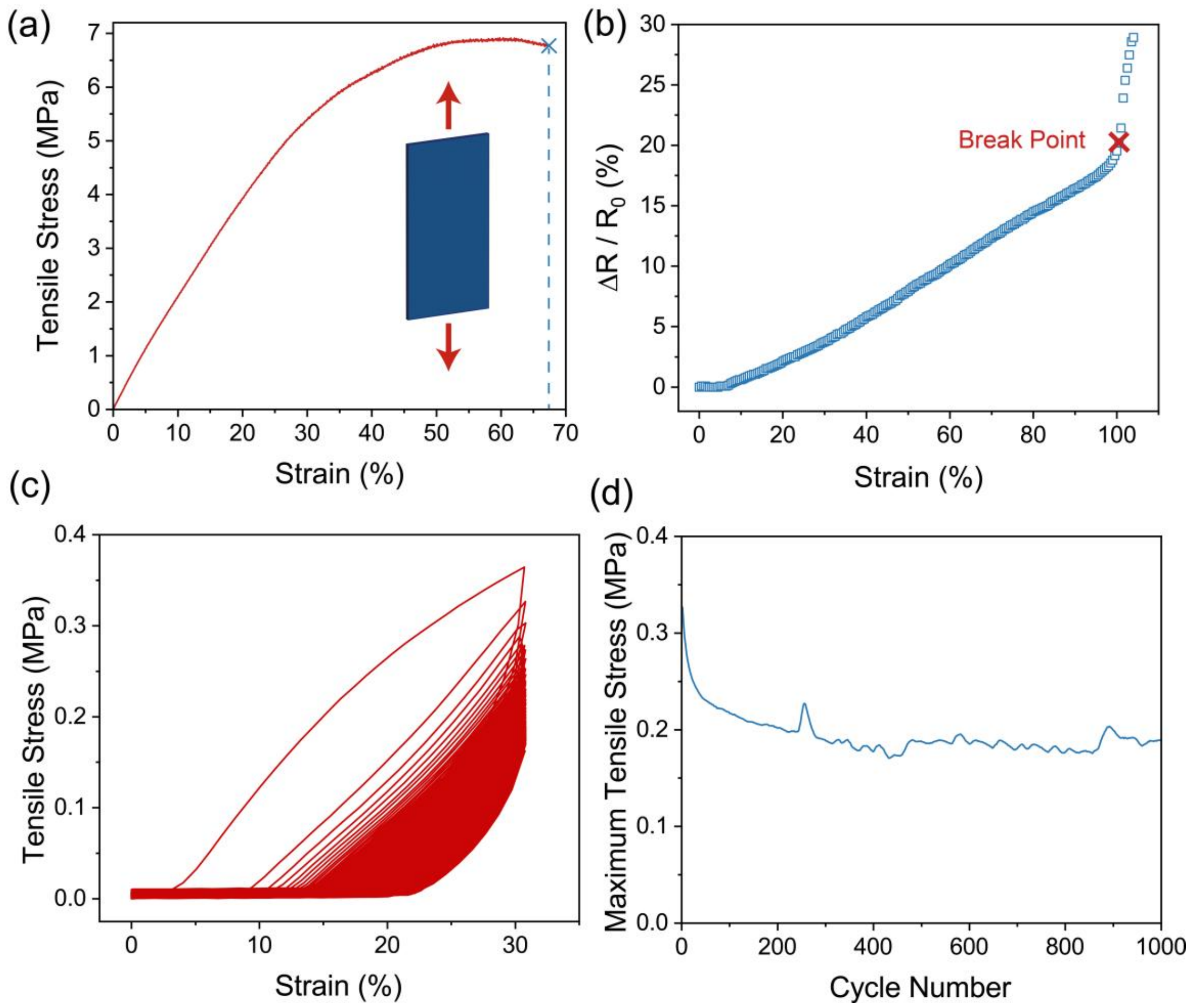

Supplementary Fig. 7. Mechanical properties of hygrotrode. (a) Tensile stress vs. strain curve of hygrotrode. Inset: scheme for stretchability characterization set-up. (b) Resistance variation of

hygrotrode till elongation at break. (c) Cyclic tensile stress vs. strain curves of hygrotrode. (d) Maximum tensile stress as a function of cycle number.

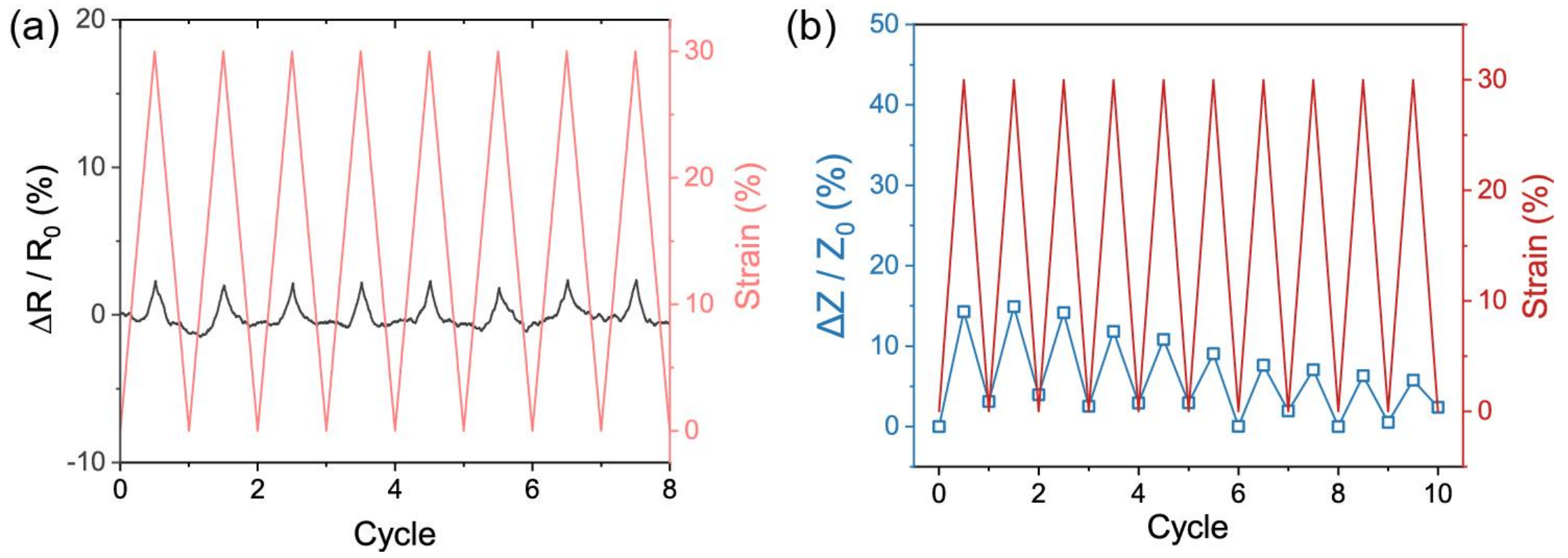

Supplementary Fig. 8. Mechanical property variation of hygrotrode during stretching-releasing cycles (maximum strain: 30%). (a) Resistance variation of hygrotrode during stretching-releasing cycles. (b) ASI variation of hygrotrode during stretching-releasing cycles.

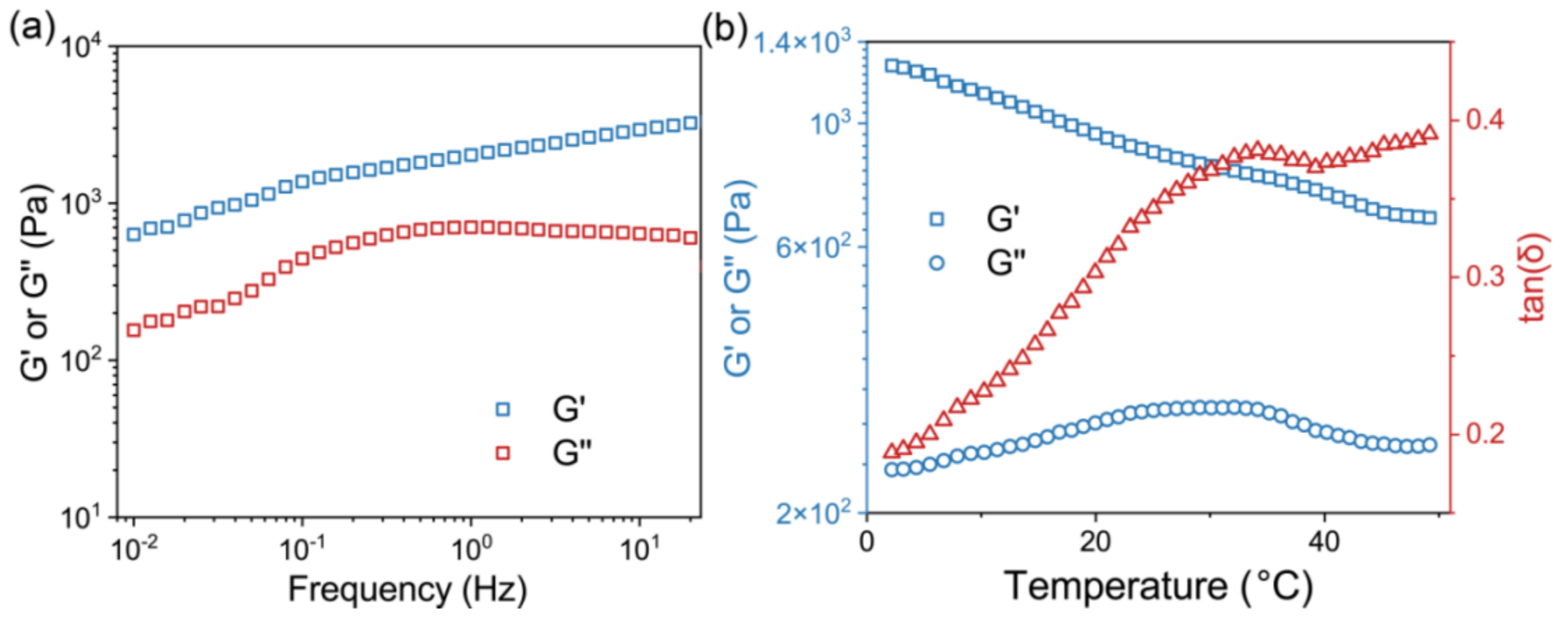

Supplementary Fig. 9. Rheology of hygrotrode. (a) Storage (G') and loss modulus (G'') change in frequency-domain (1% shear strain, 25 °C). (b) Storage and loss modulus change in temperature-domain (1% shear strain, 1 Hz). The peak of tan(δ) (ratio of the loss modulus to the storage modulus) at ~34.1 °C indicates a gel-sol transition of the hygrotrode.

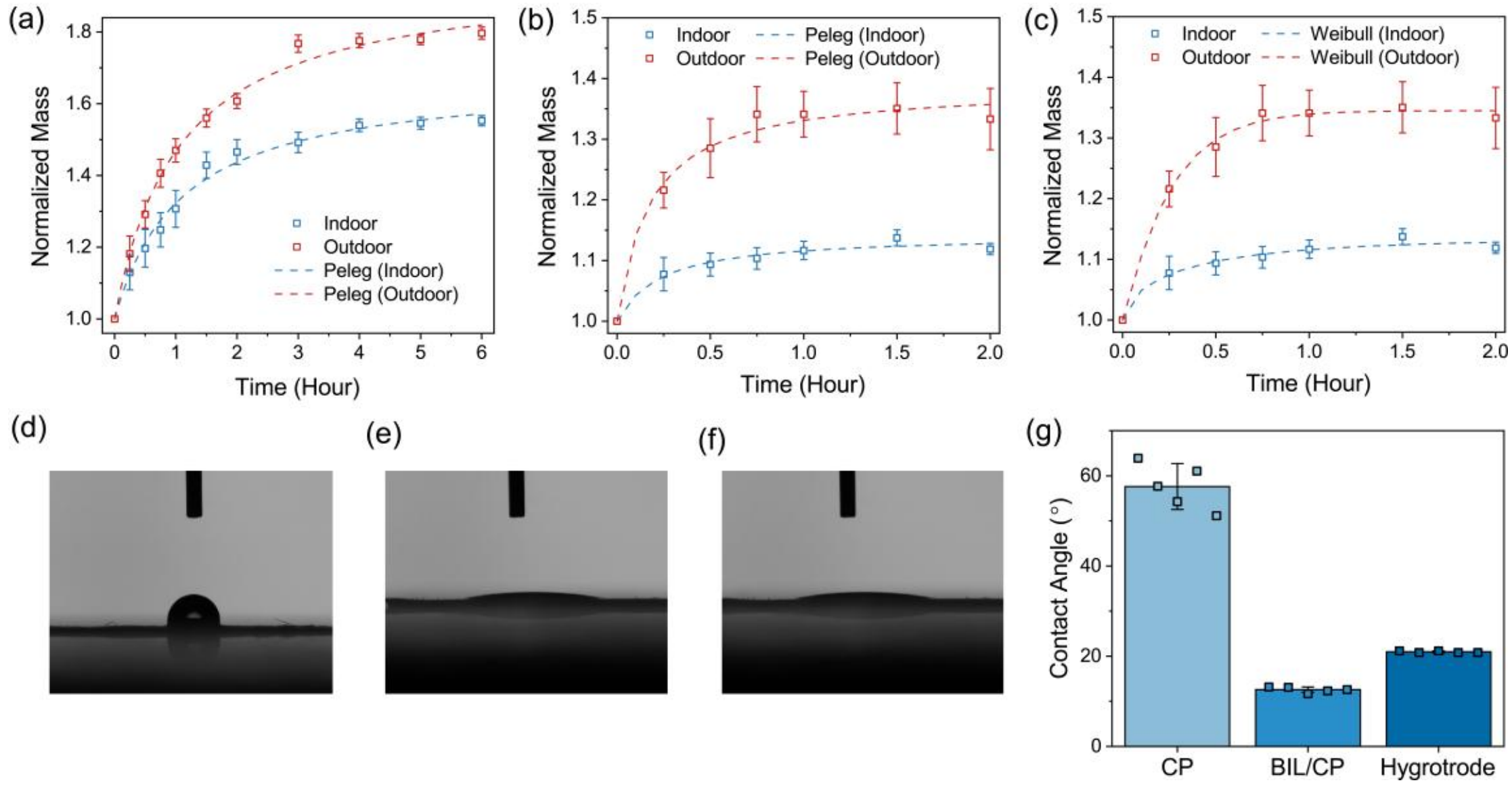

Supplementary Fig. 10. Hygroscopicity of hygrotrode and contact angle analysis. (a) Rehydration curve of hygrotrode films at indoor & outdoor environment, Peleg model is used for data fitting(*85*). Rehydration curve of on-skin hygrotrode at indoor & outdoor environment. Experimental data was fitted by (b) Peleg and (c) Weibull model. Water contact angle on (d) pristine PEDOT:PSS film, (e) BIL/CP film and (f) hygrotrode film. (g) Water contact angle comparison showed BIL-enabled high hydrophilicity, facilitating high hygroscopicity.

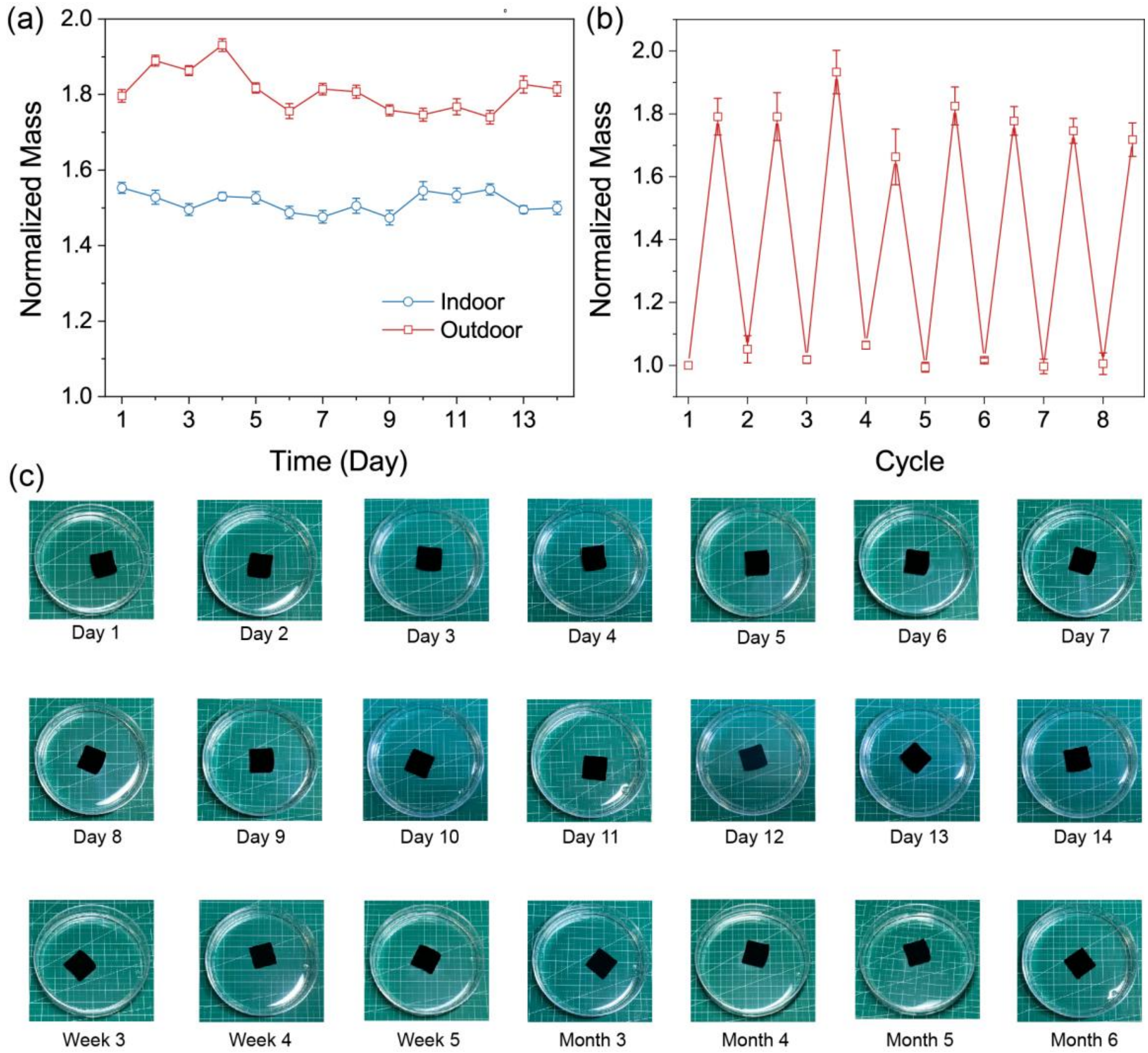

Supplementary Fig. 11. Stability of hygrotrode's hygroscopicity. (a) Change of normalized mass of hygrotrode during long-term drying process, in outdoor & indoor environment. (b) Cyclic dried and rehydration process of hygrotrode films. (c) Long-term stability of hygrotrode film in wet physiological environment confirms its property stability. hygrotrode (primary: BIL/CP = 4:1; secondary: BIL:GelMA = 1:3) shows good stability in 1X PBS more than 6 months. No obvious degradation or dissolution was observed.

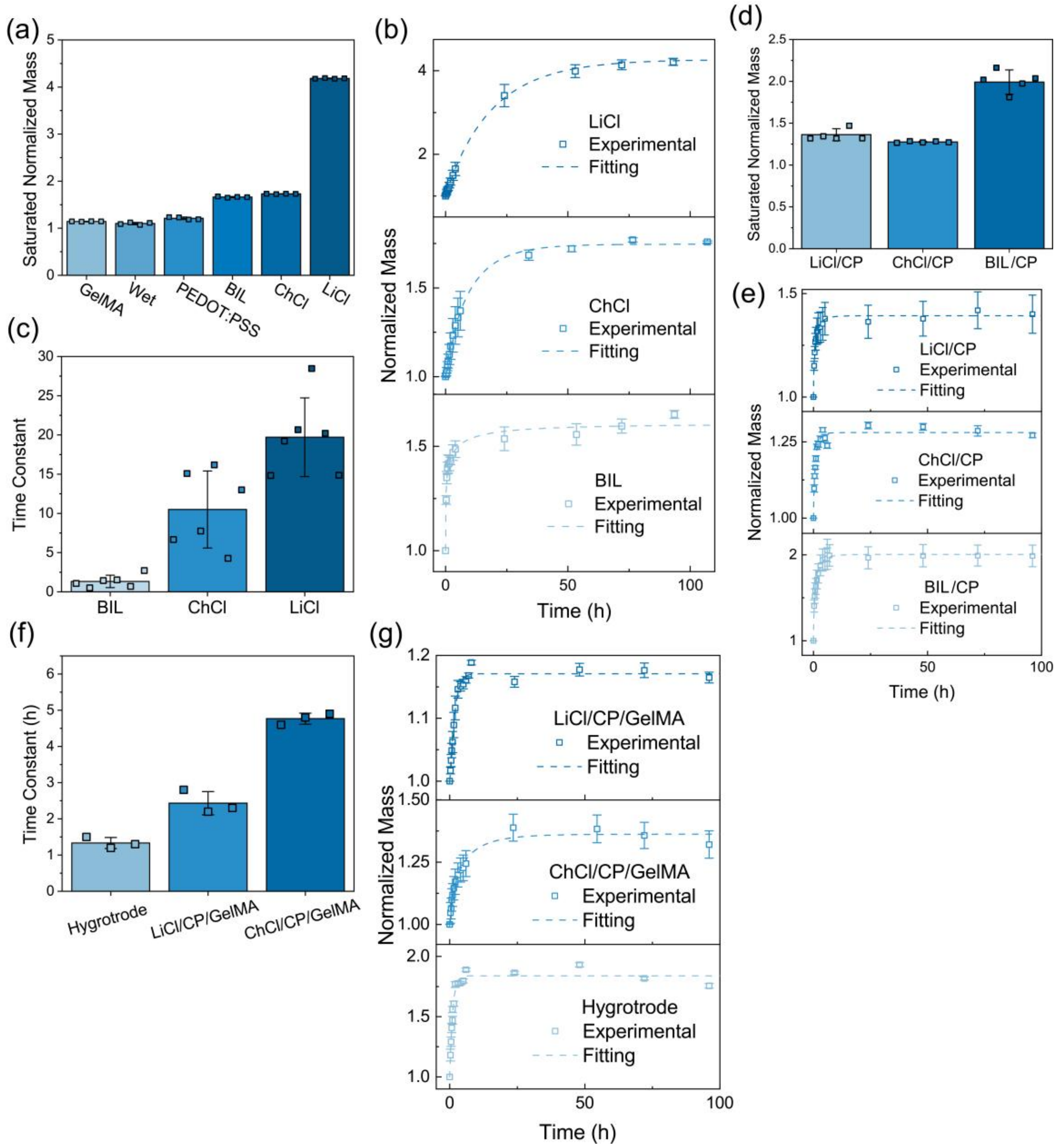

Supplementary Fig. 12. Hygroscopicity component contribution analysis. (a) SNM, (b) rehydration curve and (c) time constant of single-component system (n = 4). (d) SNM and (e) rehydration curve of binary system (n = 4). (f) Time constant and (g) rehydration curve of ternary system (n = 4). Measurements were performed at a constant temperature of 30 °C and constant relative humidity of 70% RH.

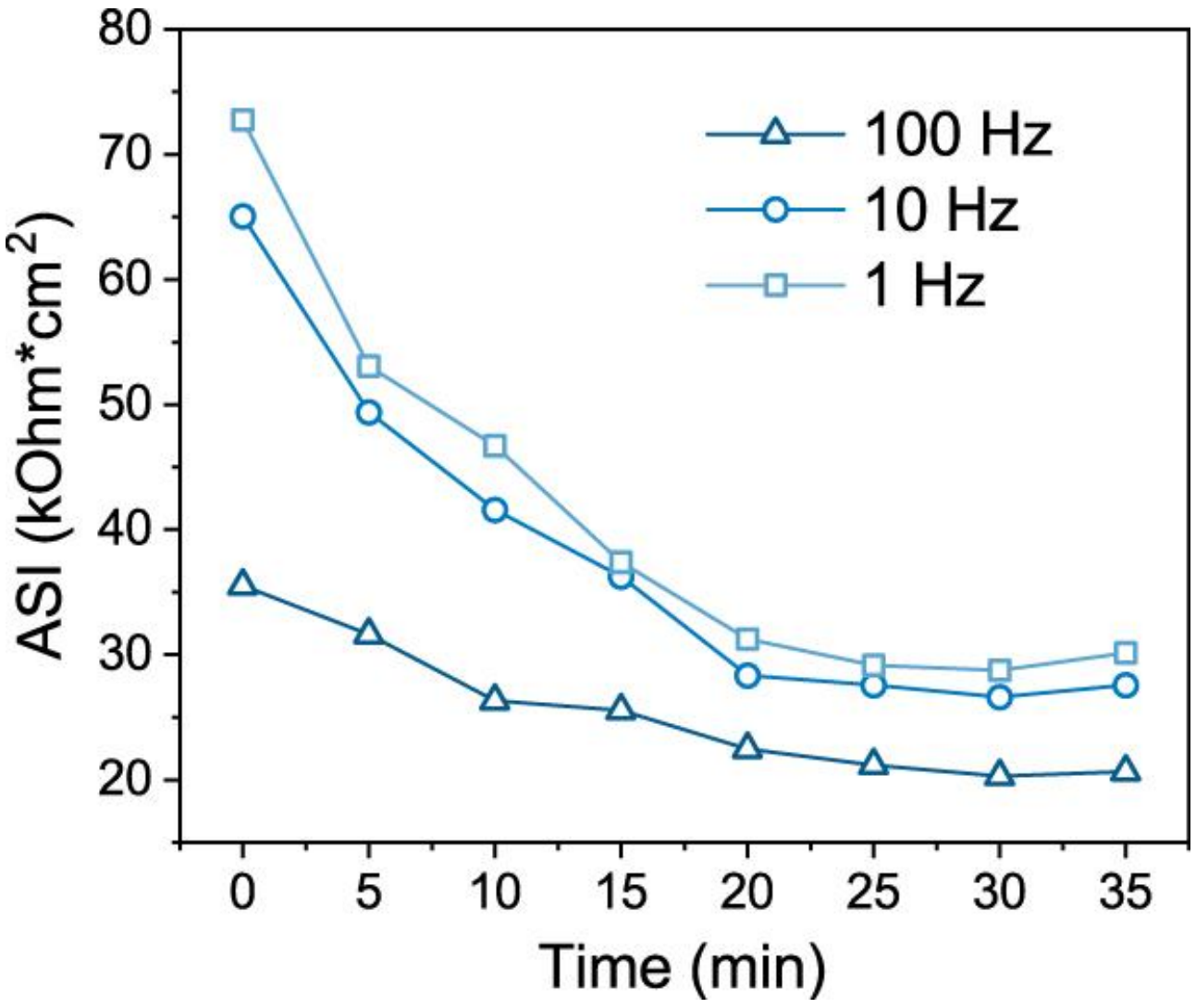

Supplementary Fig. 13. ASI change during on-skin rehydration (Bode plot).

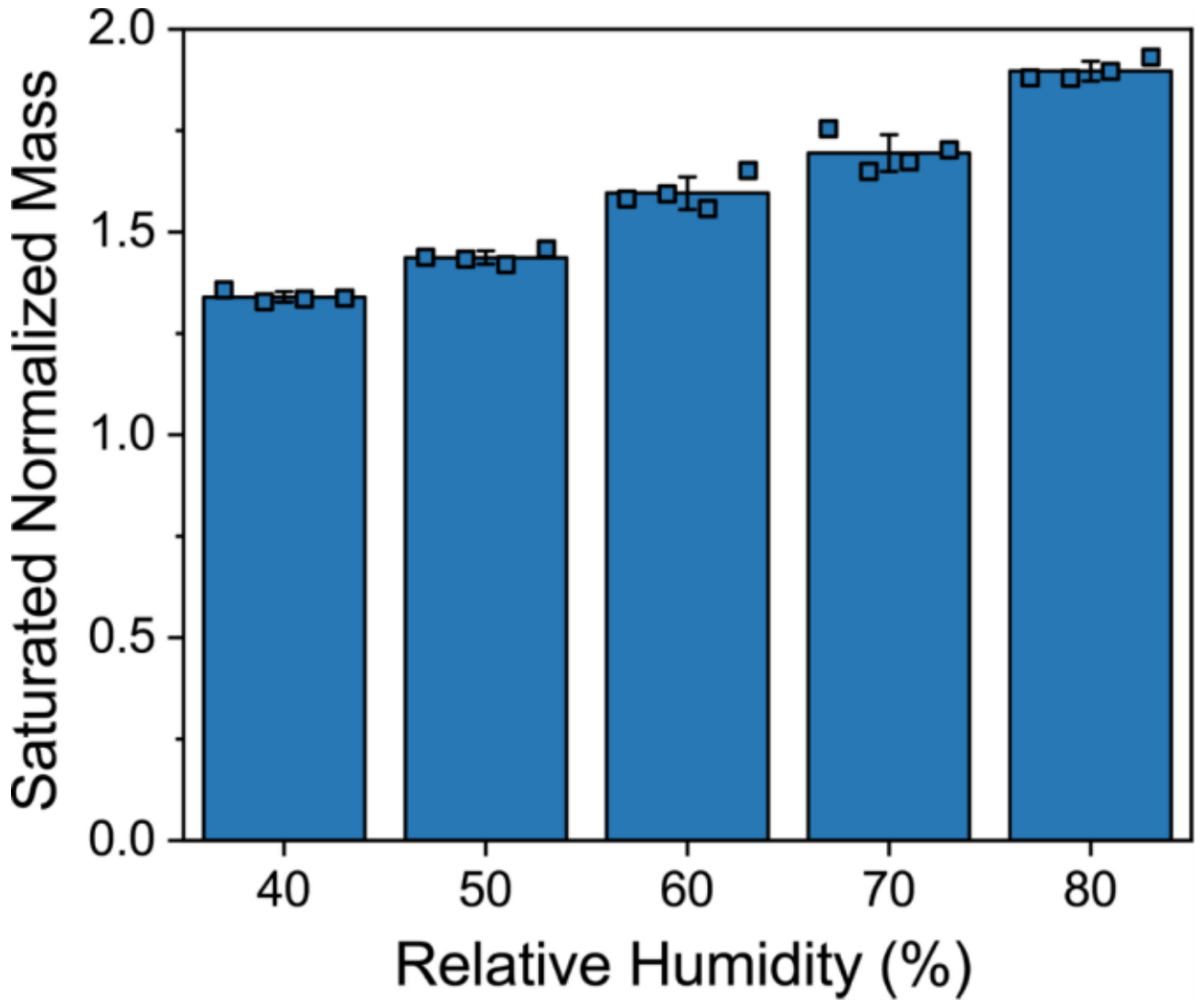

Supplementary Fig. 14. Saturated normalized mass of hygrotrode under different relative humidity.

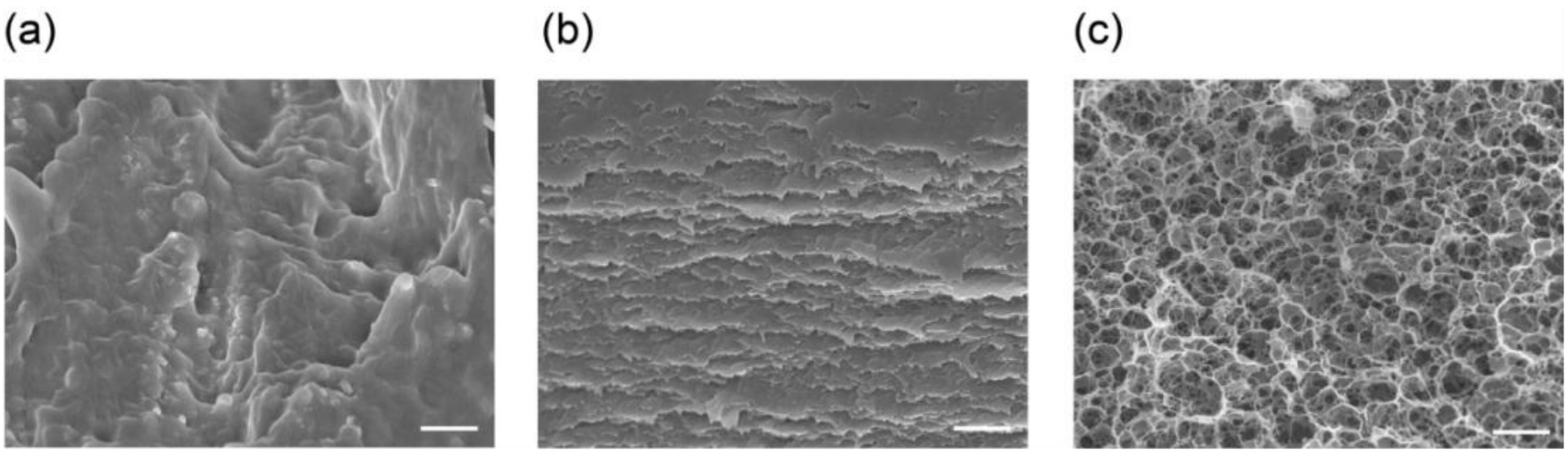

Supplementary Fig. 15. Scanning electron microscope (SEM) images of hygrotrode that (a) was dried. (b) naturally rehydrated at ambient environment. (c) rehydrated in PBS. (Scale bar: 1 μm).

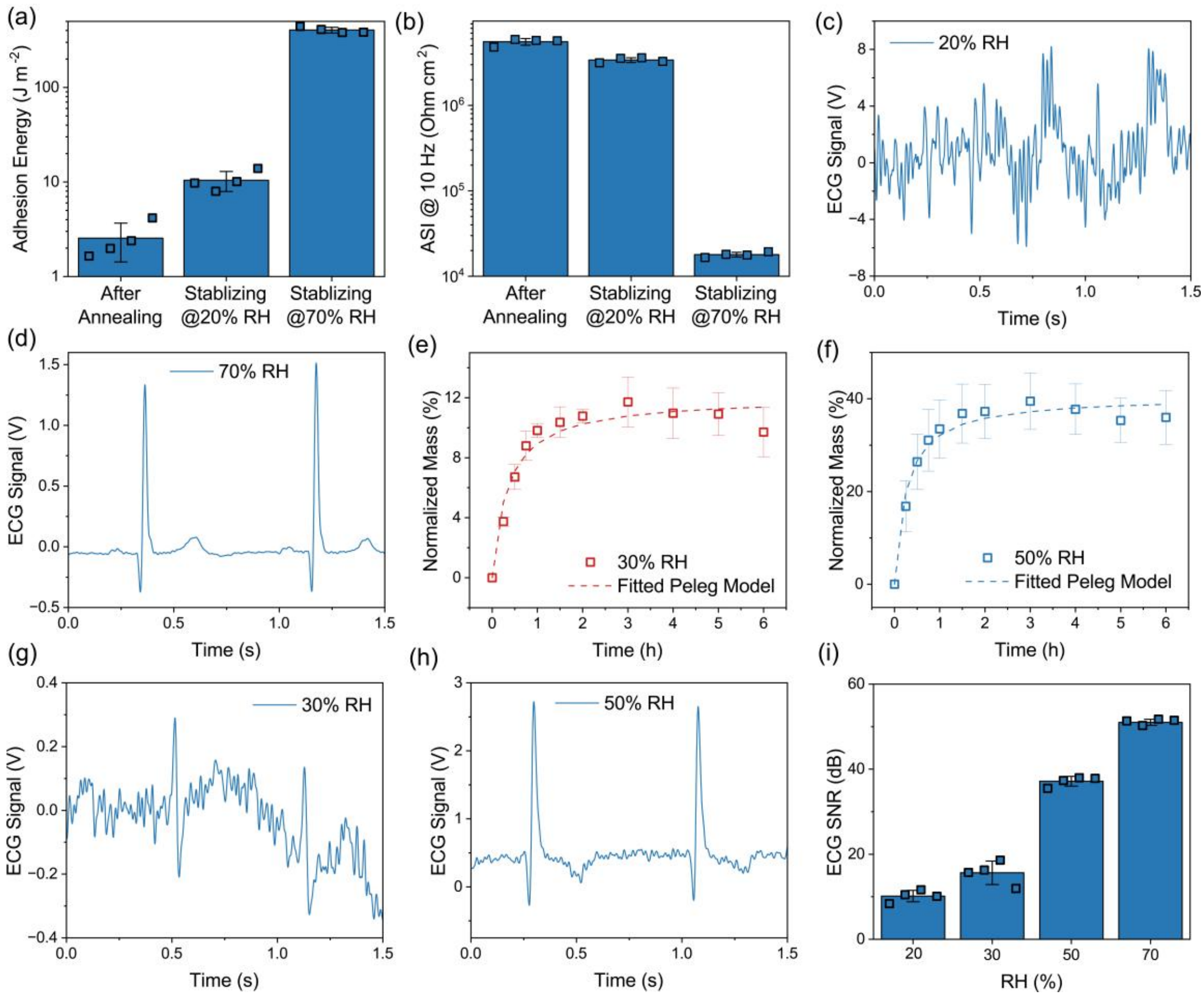

Supplementary Fig. 16 Hygroscopic behavior and ECG acquisition performance of the hygrotrode under different RH conditions. (a, b) Comparison of EIS performance and adhesion of hygrotrodes after annealing and stabilization at 20% and 70% RH. (c, d) ECG data acquired by 20% RH-stabilizing and 70% RH-stabilizing hygrotrode, respectively. (e, f) Time-dependent normalized mass change of the hygrotrode at 30% RH and 50% RH, respectively. and representative ECG signals of the hygrotrode at 50% RH. (c, d) Representative ECG signals of the hygrotrode at 30% RH and 50% RH, respectively. (i) Quantified ECG SNR as a function of RH. Data are presented as mean ± standard deviation.

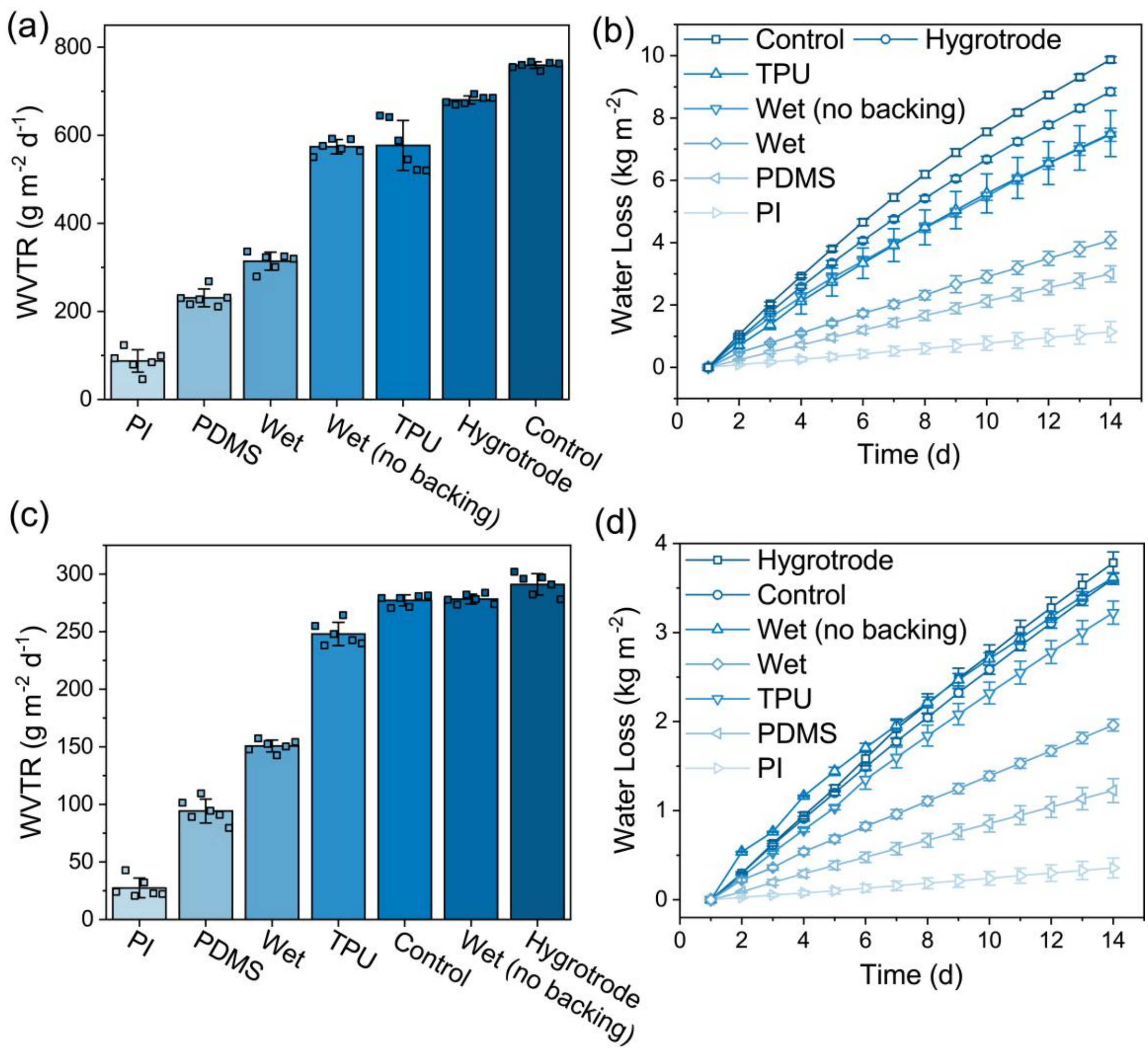

Supplementary Fig. 17. WVTR comparison among different electrodes and packaging materials. Packaging materials include polyimide (PI), polydimethylsiloxane (PDMS) and thermoplastic polyurethane (TPU). (a) WVTR comparison (T: 37℃; RH: 40%). (b) Water loss comparison of different electrode and commonly used packaging materials (T: 37℃; RH: 40%). (c) WVTR comparison (T: 25℃; RH: 50%). (d) Water loss comparison (T: 25℃; RH: 50%). The thickness of PI, PDMS, TPU and the hygrotrode are ~0.1mm. The thickness of the wet electrode (3M thin-film commercial electrode 2360) is ~1mm. The thickness of the wet electrode without backing is ~0.5mm. The 3M 2360 Red Dot™ ECG electrode consists of an Ag/AgCl-based solid conductive layer supported by a polymer backing (PET) and a medical-grade pressure-sensitive

adhesive (acrylic-based). “Control” refers to the condition without any cover on the bottle cap. The thickness of each specimen was measured using a digital caliper.

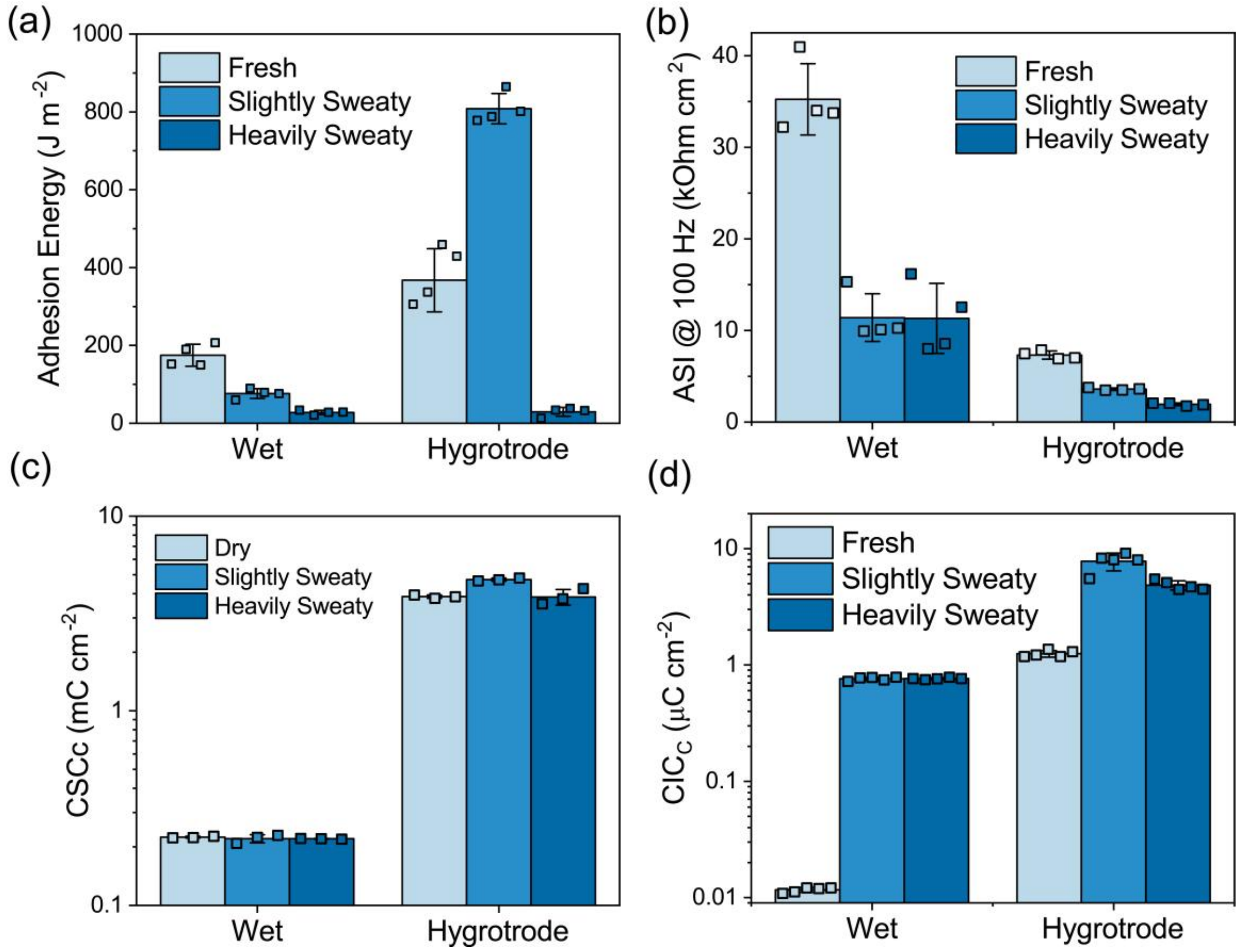

Supplementary Fig. 18. Adhesion and electrical property tested on sweaty skin. (a) Adhesion energy on skin (n = 3), (b) ASI (at 100 Hz, n = 4), (c) CSC_C (n = 3) and (d) CIC_C (n = 5) of wet electrode and hygrotrode on dry, slightly sweaty and heavily sweaty skin.

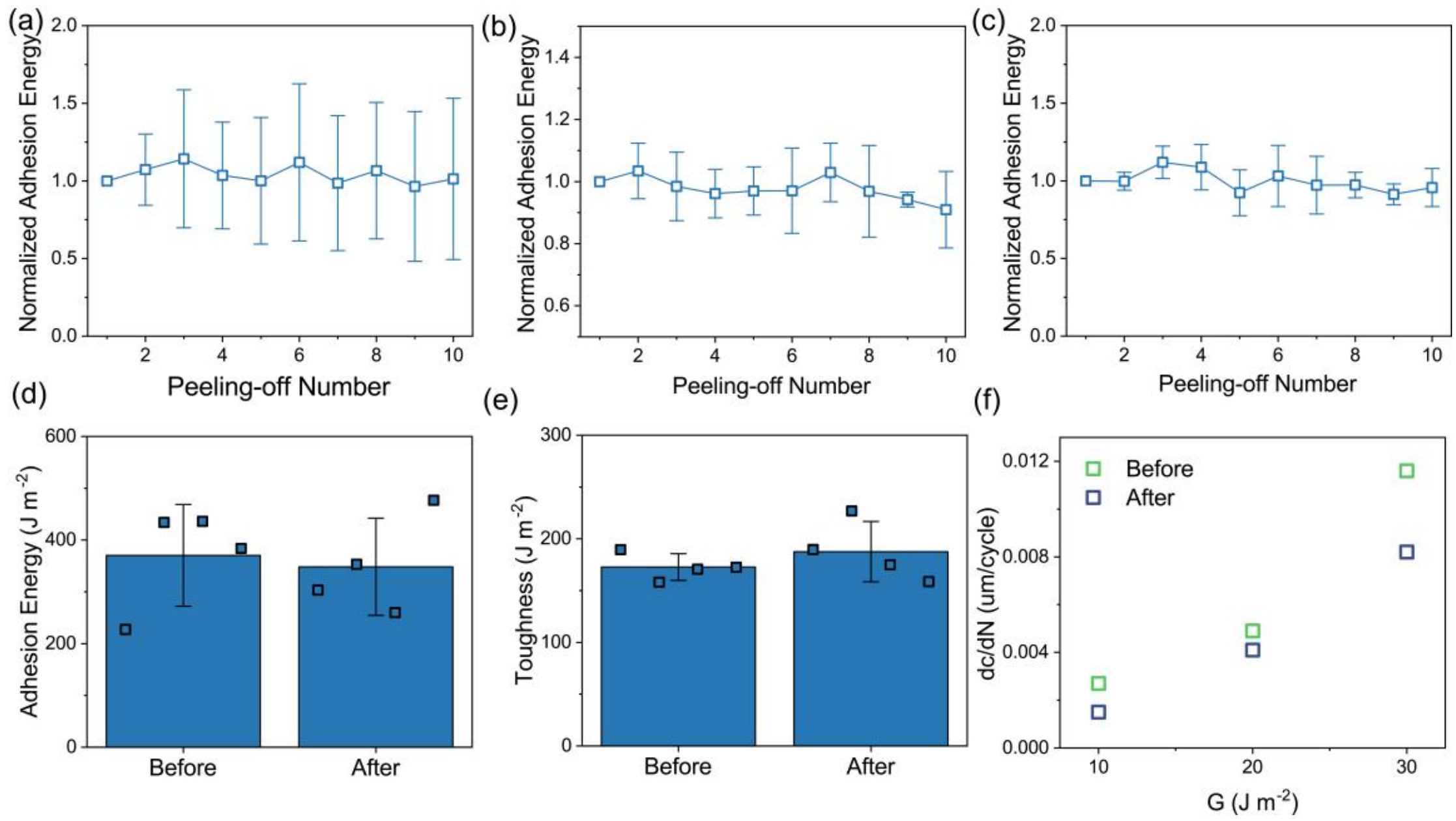

Supplementary Fig. 19. Mechanical property stability. (a) Adhesion stability of hygrotrode on PI substrate during multiple peeling-off (n = 4). (b) Adhesion stability of hygrotrode on Cu substrate during multiple peeling-off (n = 4). (c) Adhesion stability of hygrotrode on skin during multiple peeling-off (n = 4). (d, e, f) Adhesion, toughness and fatigue resistance change after prolonged wear.

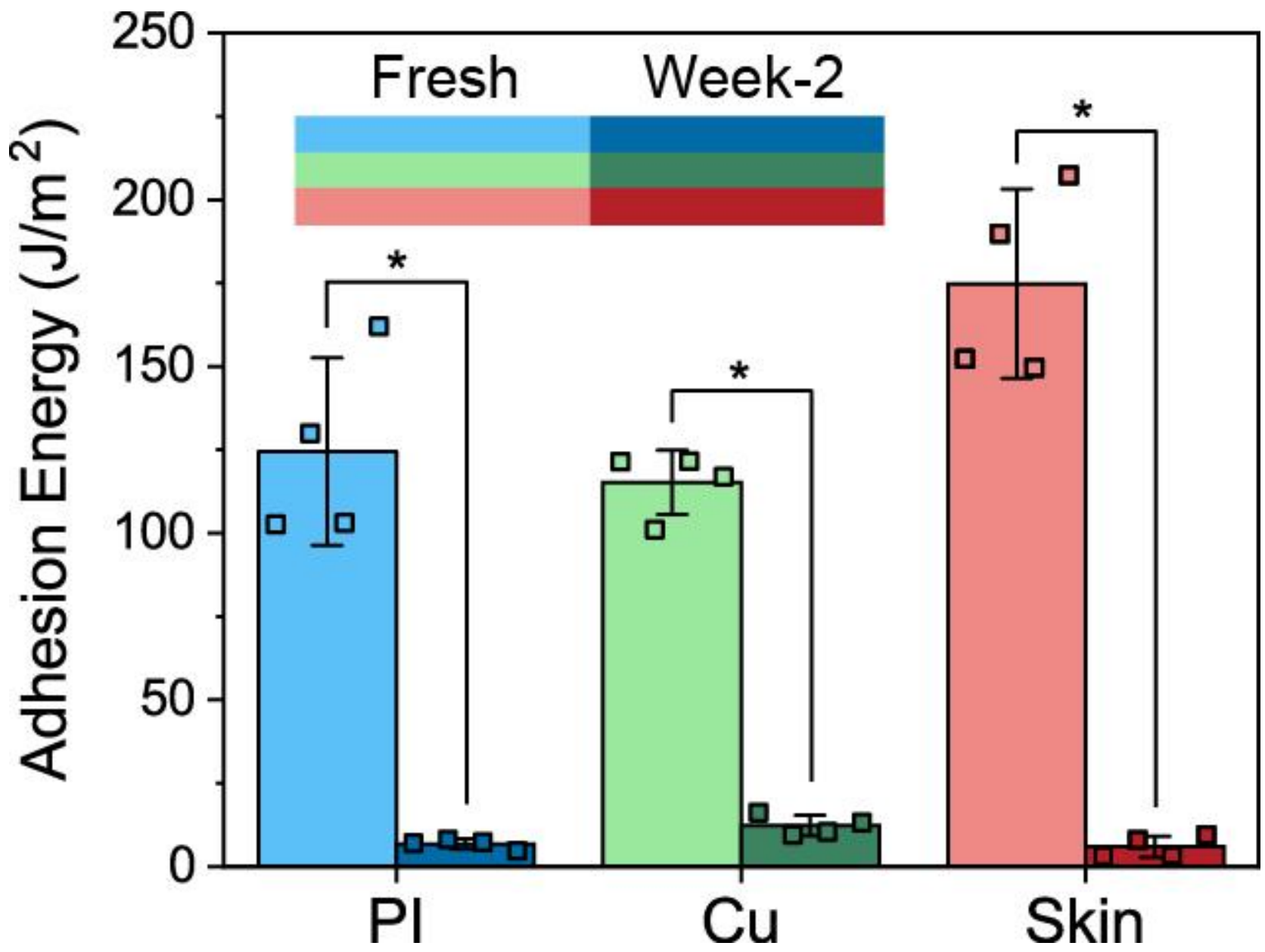

Supplementary Fig. 20. Adhesion energy change of wet electrode on different substrates in long-term period (n = 4). The *P*-values for comparison of SNR are as follows: for wet electrode

on PI, $P = 1.61\times10^{-4}$; for wet electrode on Cu, $P = 9.32\times10^{-7}$; for wet electrode on skin, $P = 2.22\times10^{-5}$. $^*P < 0.01$. All error bars denote the s.d.

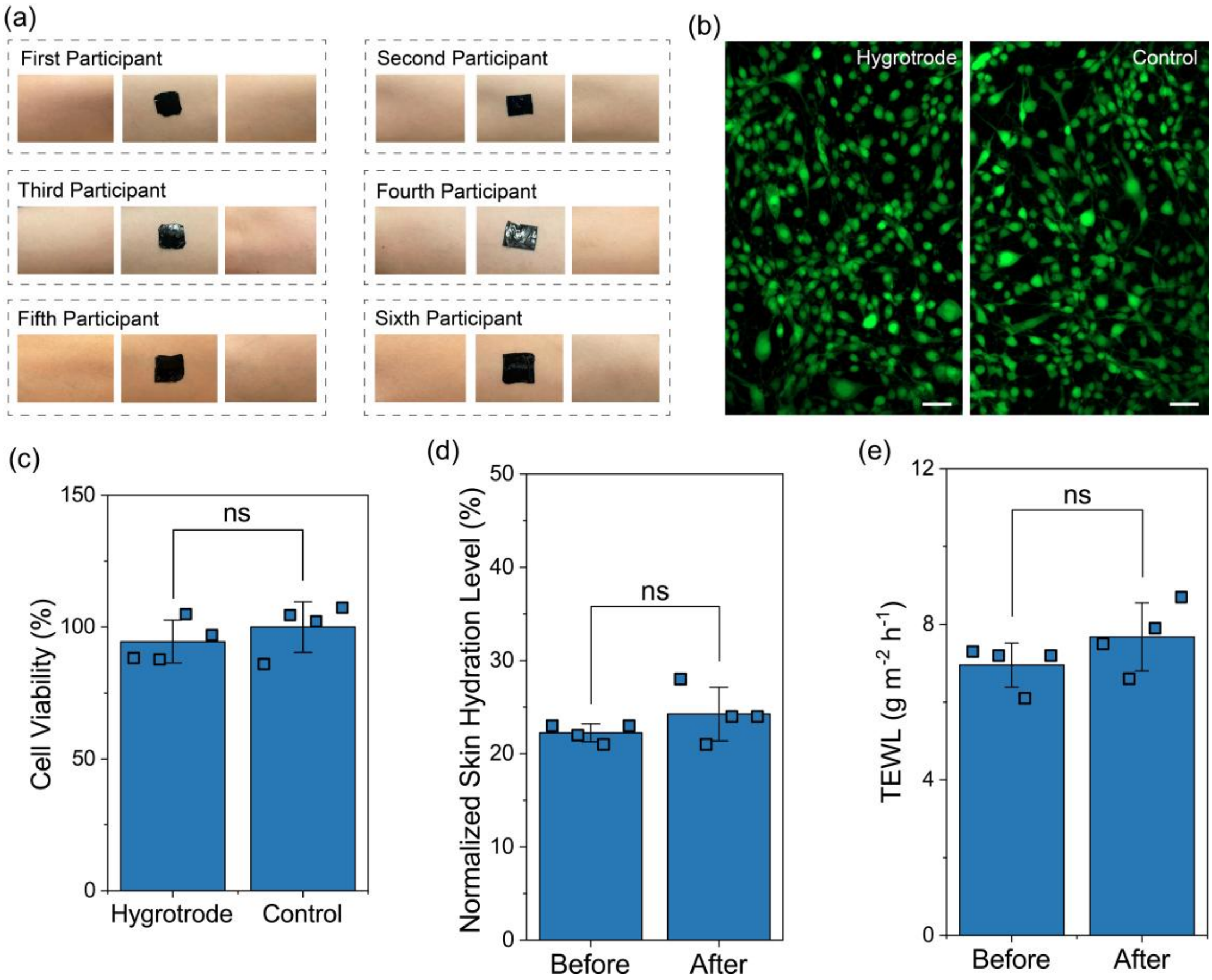

Supplementary Fig. 21 Biocompatibility evaluation. (a) Skin irritation assessment (n=6). Representative photographs of the skin (left) before application, (middle) during wear, and (right) after 8 h of continuous wear. (b) Representative stained images of 3T3 cells seeded on the hygrotrode 3 days post-seeding. (c) Quantitative analysis of 3T3 cell viability revealed no statistically significant differences ($p > 0.05$). (a) Skin hydration level and (b) TEWL change before and after hygrotrode peeling-off revealed no statistically significant differences ($p > 0.05$).

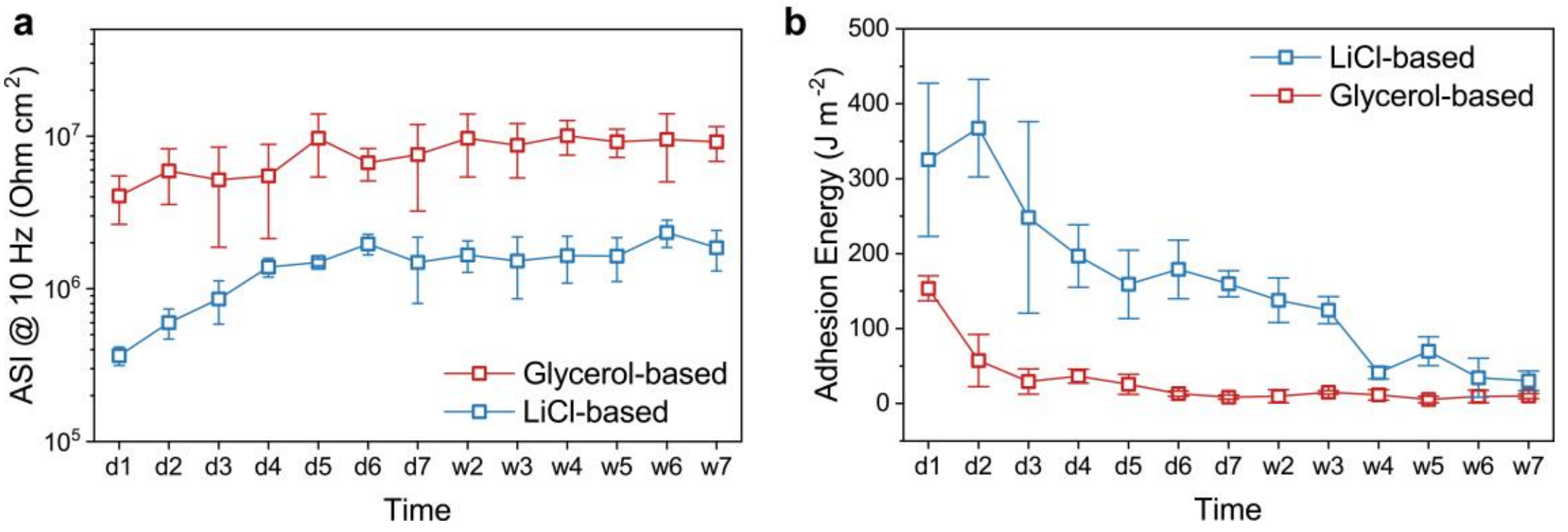

Supplementary Fig. 22 Long-term (a) ASI and (b) adhesion energy evaluation of LiCl-based and glycerol-based hygroscopic electrode.

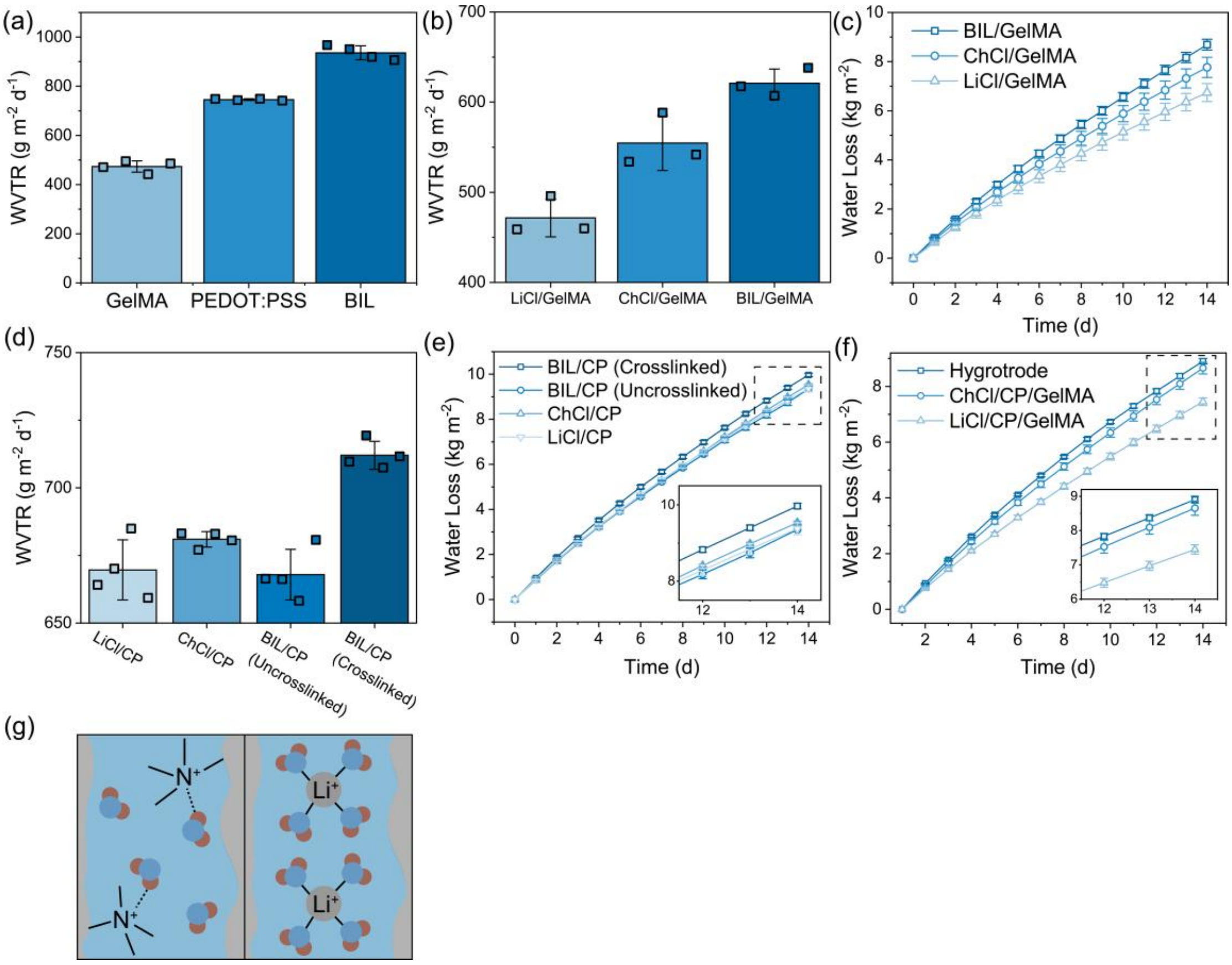

Supplementary Fig. 23. WVTR component contribution analysis. (a) WVTR of single-component system (n = 4). (b) WVTR and (c) water-loss of HM/GelMA binary system (n = 4). (d) WVTR and (e) water-loss of HM/CP binary system (n = 4). (f) WVTR of ternary system (n = 4). (g) Schematics of different water state in crosslinked BIL and LiCl-based hydrogel.

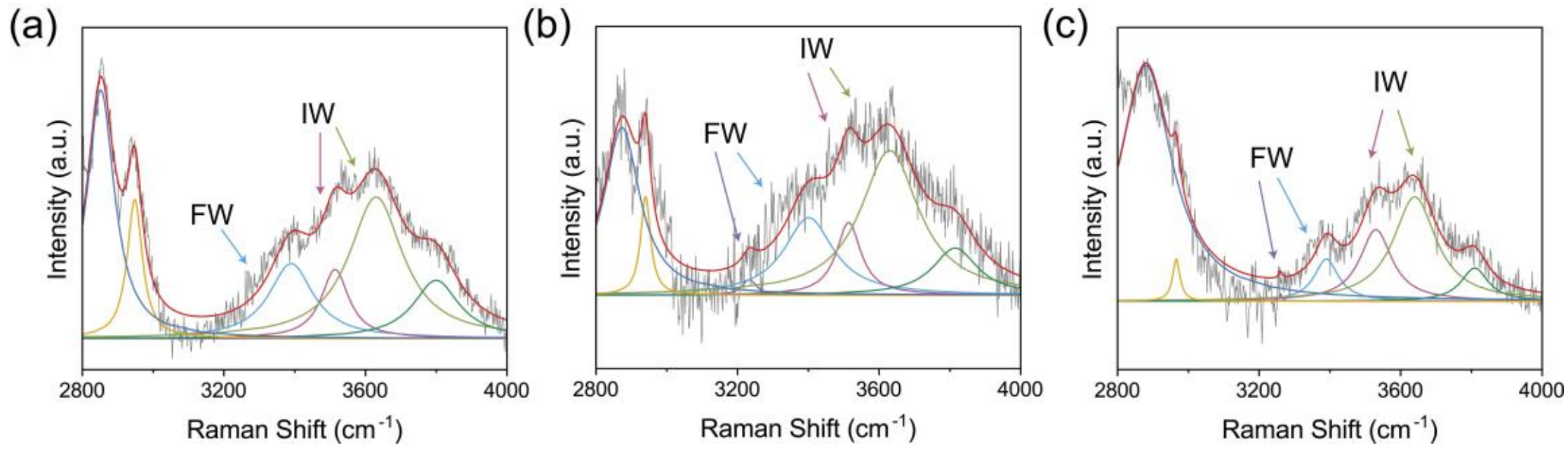

Supplementary Fig. 24 Raman spectra of (a) BIL-, (b) ChCl- and (c) LiCl-based hygroscopic systems (water content: 10 g g-1). The peaks at 3233 and 3401 cm−1 are attributed to free water (FW), while the peaks at 3514 and 3630 cm−1 correspond to intermediate water (IW).

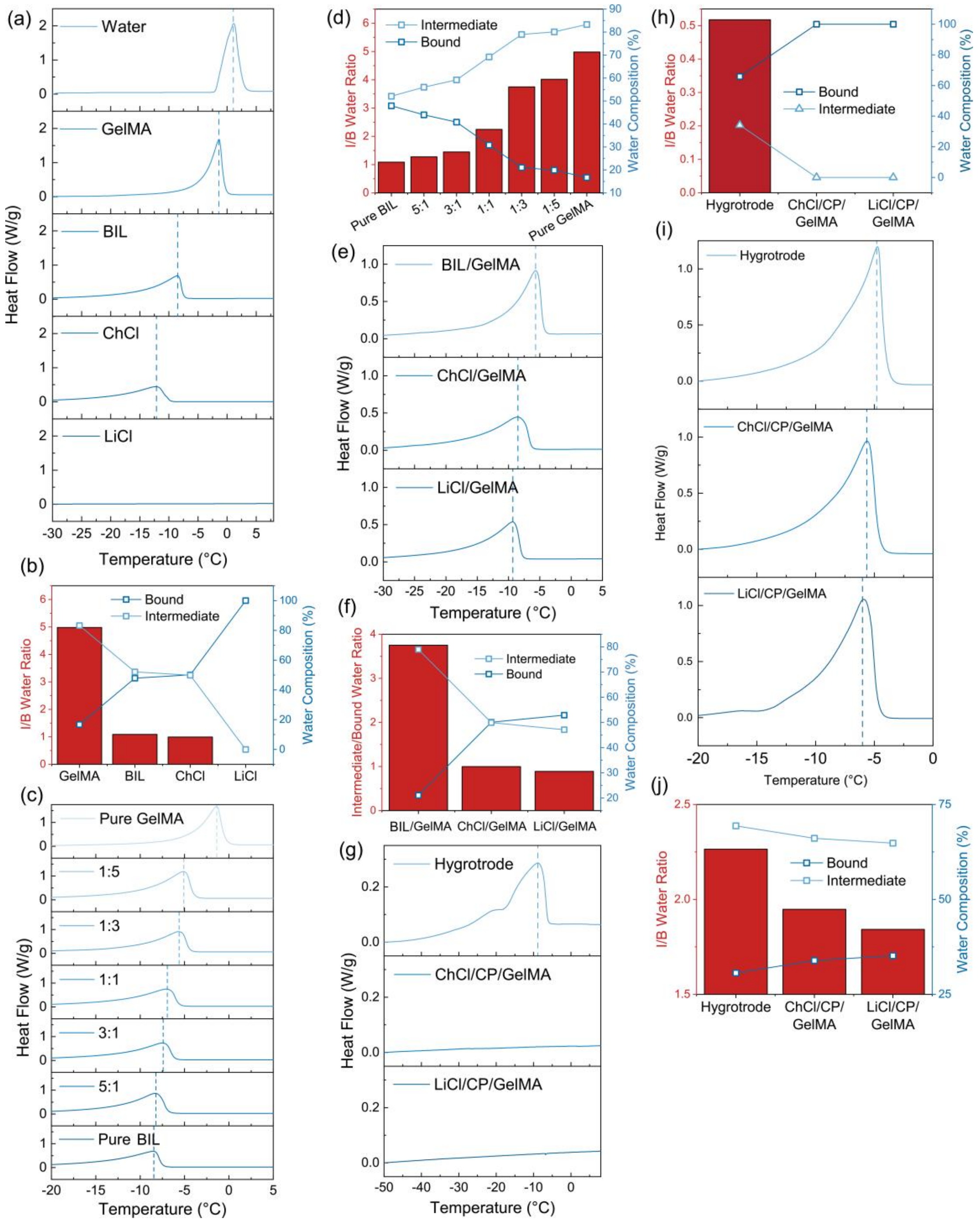

Supplementary Fig. 25. DSC analysis on water status (water content is 3 g g^{-1} unless stated).

(a) DSC spectra of single-component systems. (b) Ratio of IW to BW and their corresponding composition in single-component systems. (c) DSC spectra of BIL/GelMA system with different mass ratio. (d) Ratio of IW to BW and their corresponding composition in BIL/GelMA system with different mass ratio. (e) DSC spectra of HM/GelMA binary systems. (f) Ratio of IW to BW and their

corresponding composition in HM/GelMA binary systems. (g) DSC spectra of ternary systems (water content: 1 g g^{-1}, close to the saturation level). (h) Ratio of IW to BW and their corresponding composition in ternary systems (water content: 1 g g^{-1}). IW was only observed in hygrotrode among all tested ternary systems. (i) DSC spectra of ternary systems (water content: 2 g g^{-1}). (j) Ratio of IW to BW and their corresponding composition in ternary systems (water content: 2 g g^{-1}).

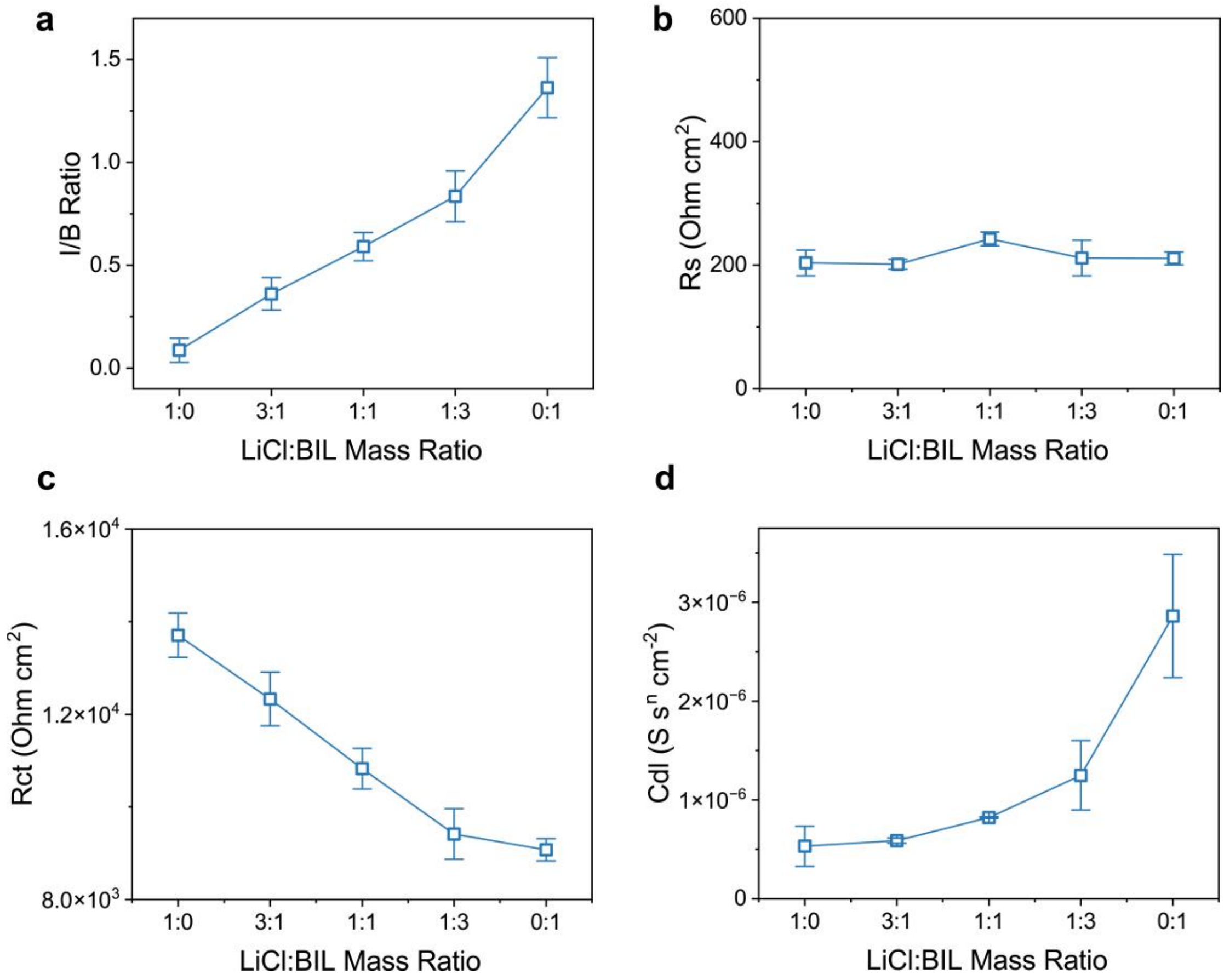

Supplementary Fig. 26 Correlation between water-state distribution and electrochemical impedance parameters of hygrotrodes with different LiCl:BIL ratios. (a) I/B ratio determined for hygrotrodes with varying LiCl:BIL mass ratios. (b–d) Electrochemical impedance parameters (R_s, R_{ct}, and C_{dl}) obtained from Randles-model fitting of the EIS spectra, shown as a function of the LiCl:BIL mass ratio. All samples were prepared with identical total water content (1 g g^{-1}). Data are presented as mean ± standard deviation.

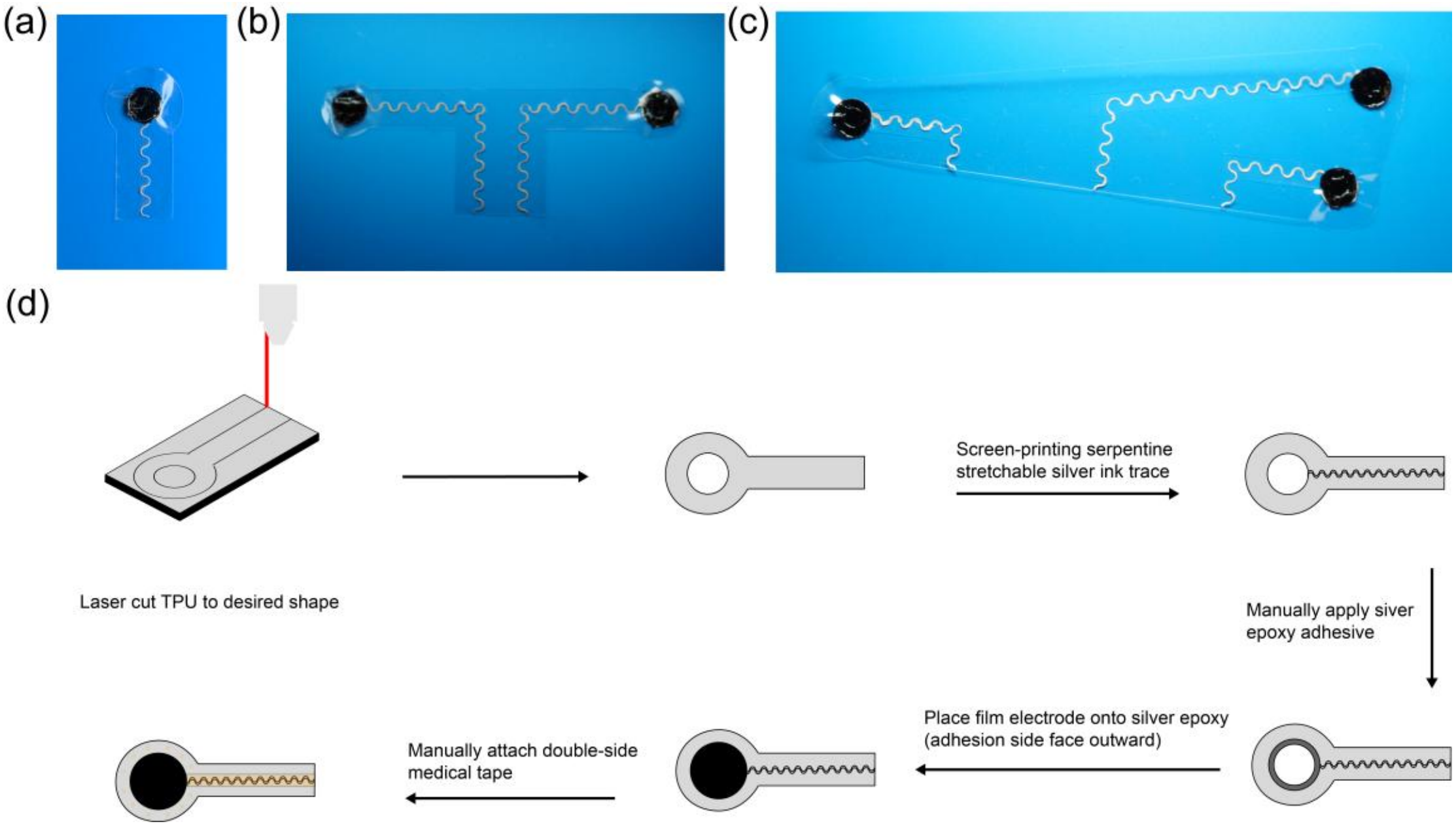

Supplementary Fig. 27. Electrode array design. Optical image of (a) single-electrode patch for EOG monitoring, (b) electrode patch for ECG monitoring and (c) electrode array for fore-head EEG monitoring. (d) Fabrication process for hygrotrode device (using one-channel electrode as example).

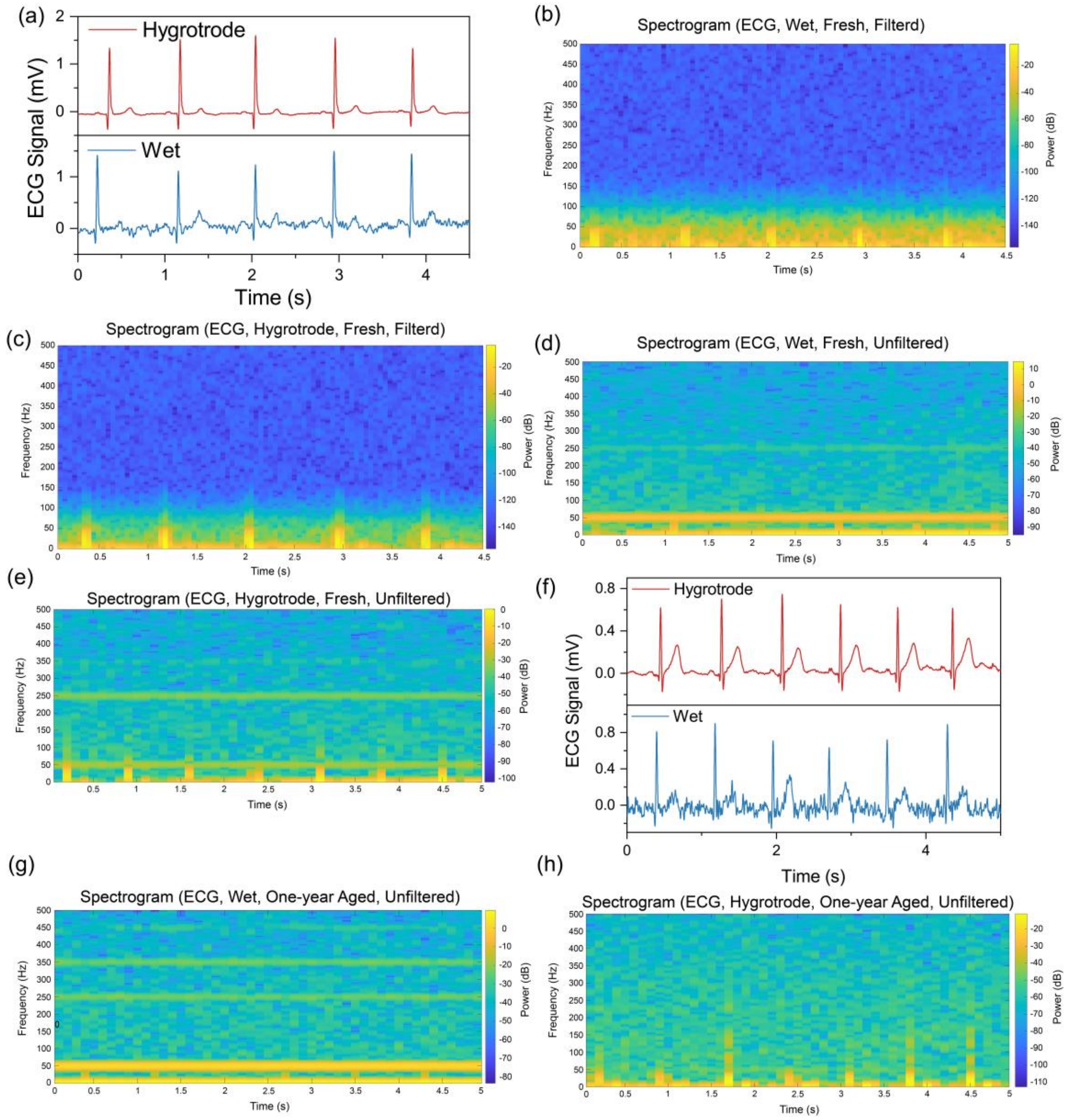

Supplementary Fig. 28. Filtered ECG signals and their spectrograms. (a) Filtered ECG data acquired by fresh wet electrode & hygrotrode. (b) Spectrogram plot of ECG signal acquired by fresh wet electrode. (c) Spectrogram plot of ECG signal acquired by fresh hygrotrode. (d) Spectrogram plot of unfiltered ECG signal acquired by fresh wet electrode. (e) Spectrogram plot of unfiltered ECG signal acquired by fresh hygrotrode. (f) Filtered ECG data acquired by one-year aged wet electrode & hygrotrode. (g) Spectrogram plot of unfiltered ECG signal acquired by one-year aged wet electrode. (h) Spectrogram plot of unfiltered ECG signal acquired by one-year aged hygrotrode.(e) Comparison of signal-to-noise ratio (SNR) of filtered ECG data acquired by wet

electrode & hygrotrode, which are fresh or one-year aged. Unpaired, two tailed t-test (n=5) was used for statistical analysis. The P values for comparison of SNR are as follows: for fresh wet electrode versus one-year aged wet electrode, P = 0.01476; for fresh hygrotrode versus one-year aged hygrotrode, P = 0.9763; for fresh wet electrode versus fresh hygrotrode, $P = 4.45\times10^{-3}$; for one-year aged wet electrode versus one-year aged hygrotrode, $P = 1.62\times10^{-6}$. *P < 0.05; **P < 0.01. NS, not significant. All error bars denote the s.d.

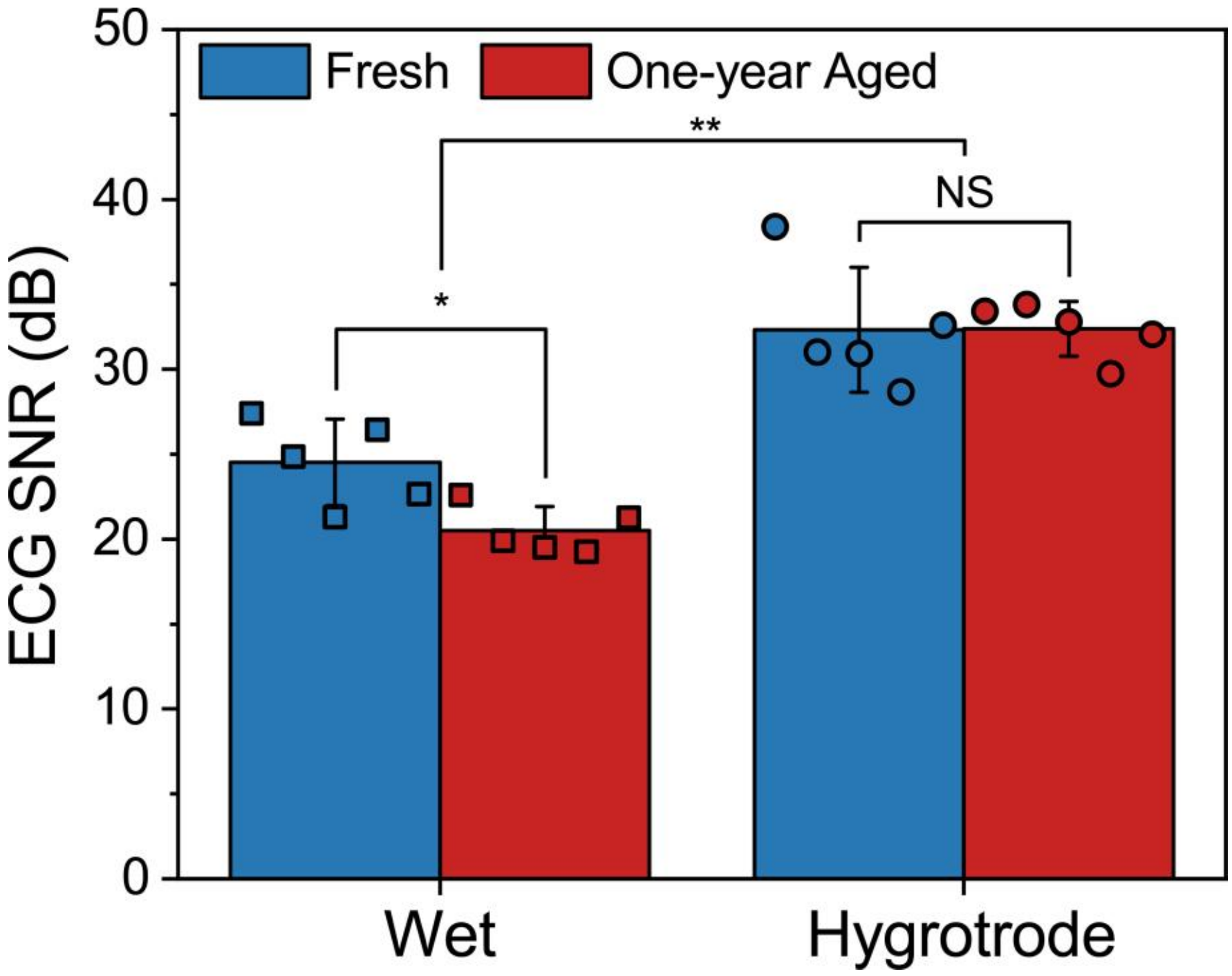

Supplementary Fig. 29. Comparison of SNR of filtered ECG data acquired by wet electrode & hygrotrode, which are fresh or one-year aged. Unpaired, two tailed t-test (n=5) was used for statistical analysis. The P values for comparison of SNR are as follows: for fresh wet electrode versus one-year aged wet electrode, P = 0.01476; for fresh hygrotrode versus one-year aged hygrotrode, P = 0.9763; for wet electrode and hygrotrode, P < 0.0001. *P < 0.05; **P < 0.0001. NS, not significant. All error bars denote the s.d.

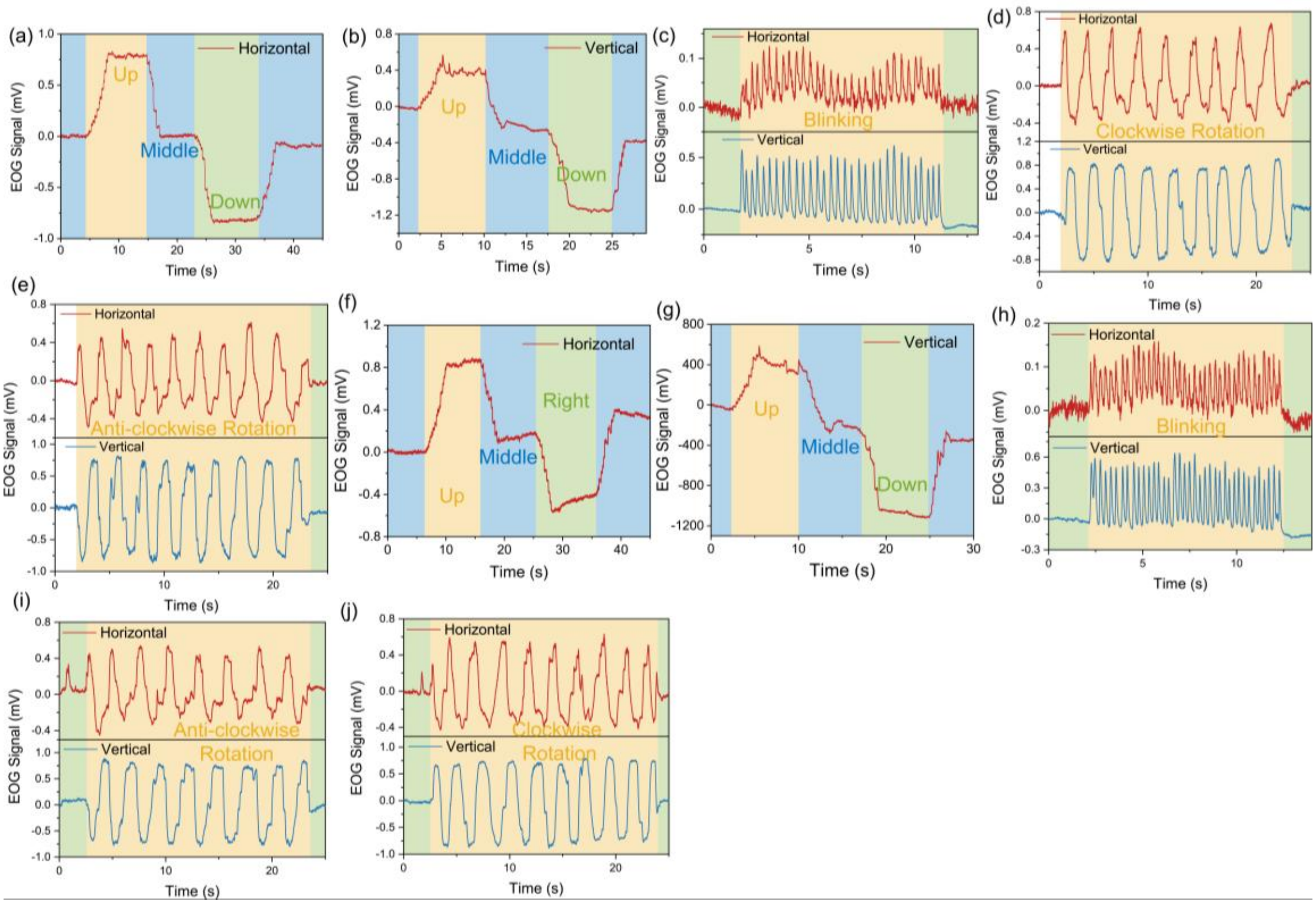

Supplementary Fig. 30. EOG signals. Fresh hygrotrode-acquire EOG signal generated by (a) left-middle-right eyeball horizontal movement, (b) up-middle-down eyeball vertical movement, (c) blinking, (d) clockwise eyeball rotation and (e) anti-clockwise eyeball rotation. One-year aged hygrotrode-acquired EOG signal generated by (f) left-middle-right eyeball horizontal movement, (g) up-middle-down eyeball vertical movement, (h) blinking, (i) clockwise eyeball rotation and (j) anti-clockwise eyeball rotation.

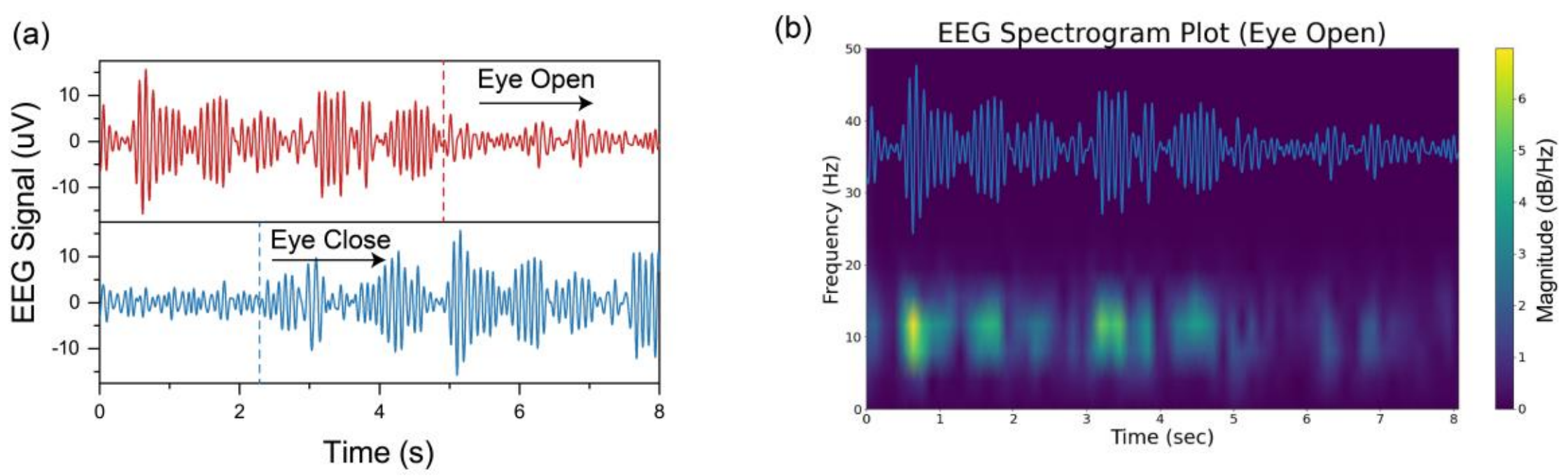

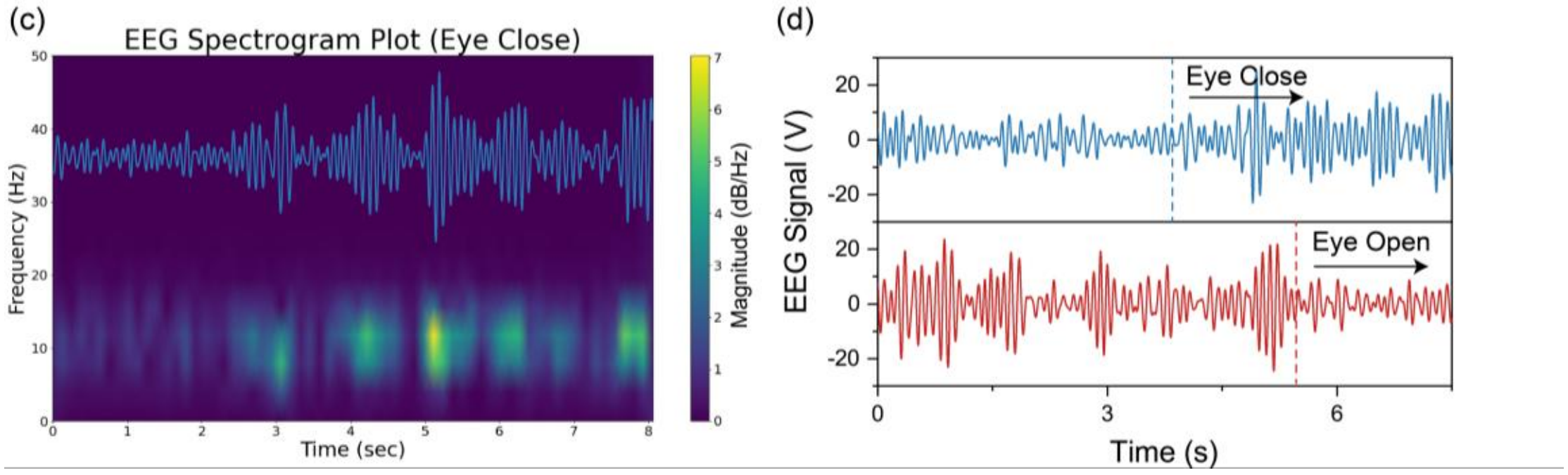

Supplementary Fig. 31. EEG signals and spectrograms. (a) EEG data acquired by fresh hygrotrode patch. (b) Spectrogram plot of EEG signal when performing eye-close movement. (c) Spectrogram plot of EEG signal when performing eye-open movement. (d) EEG data acquired by one-year aged hygrotrode patch.

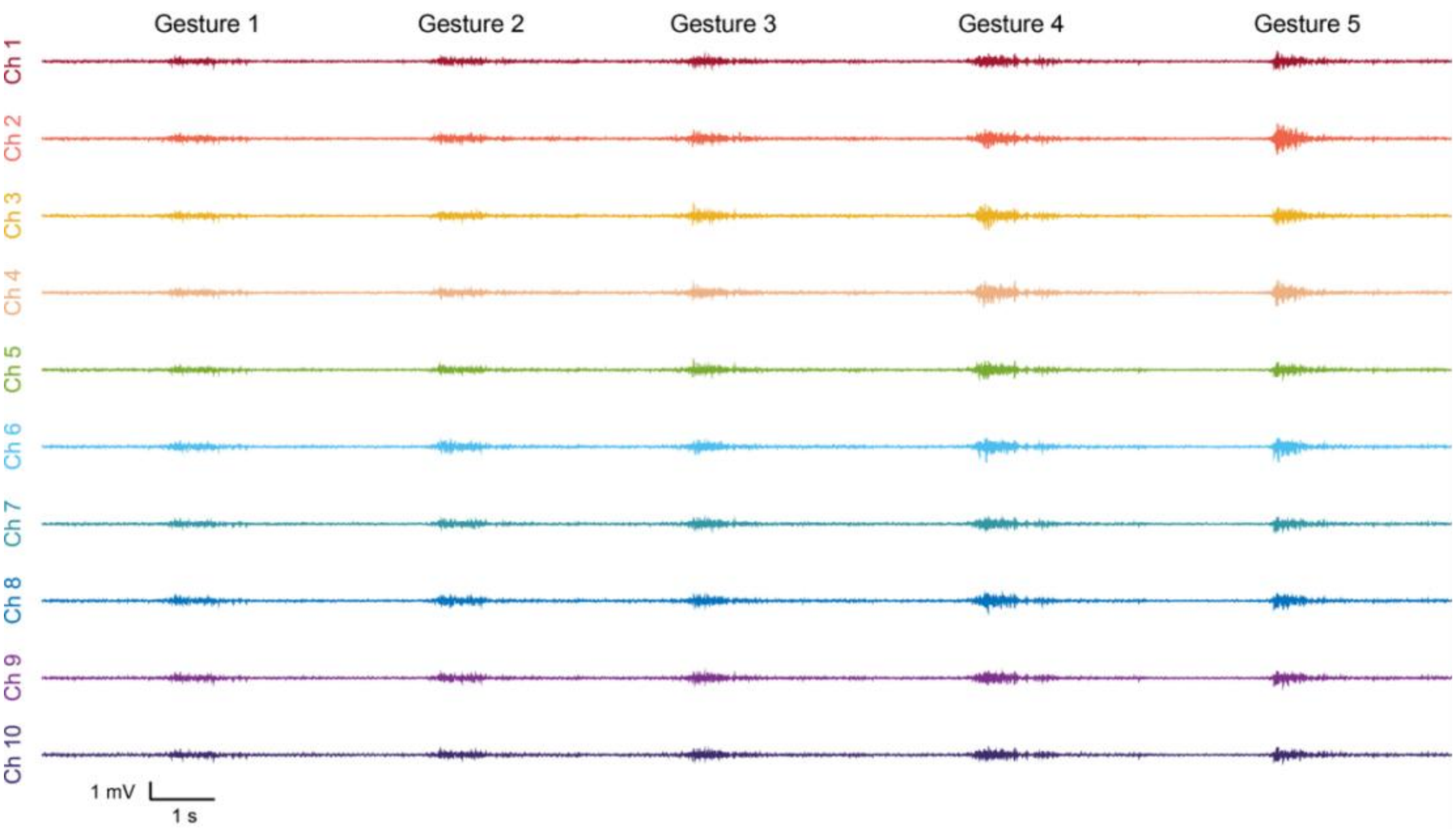

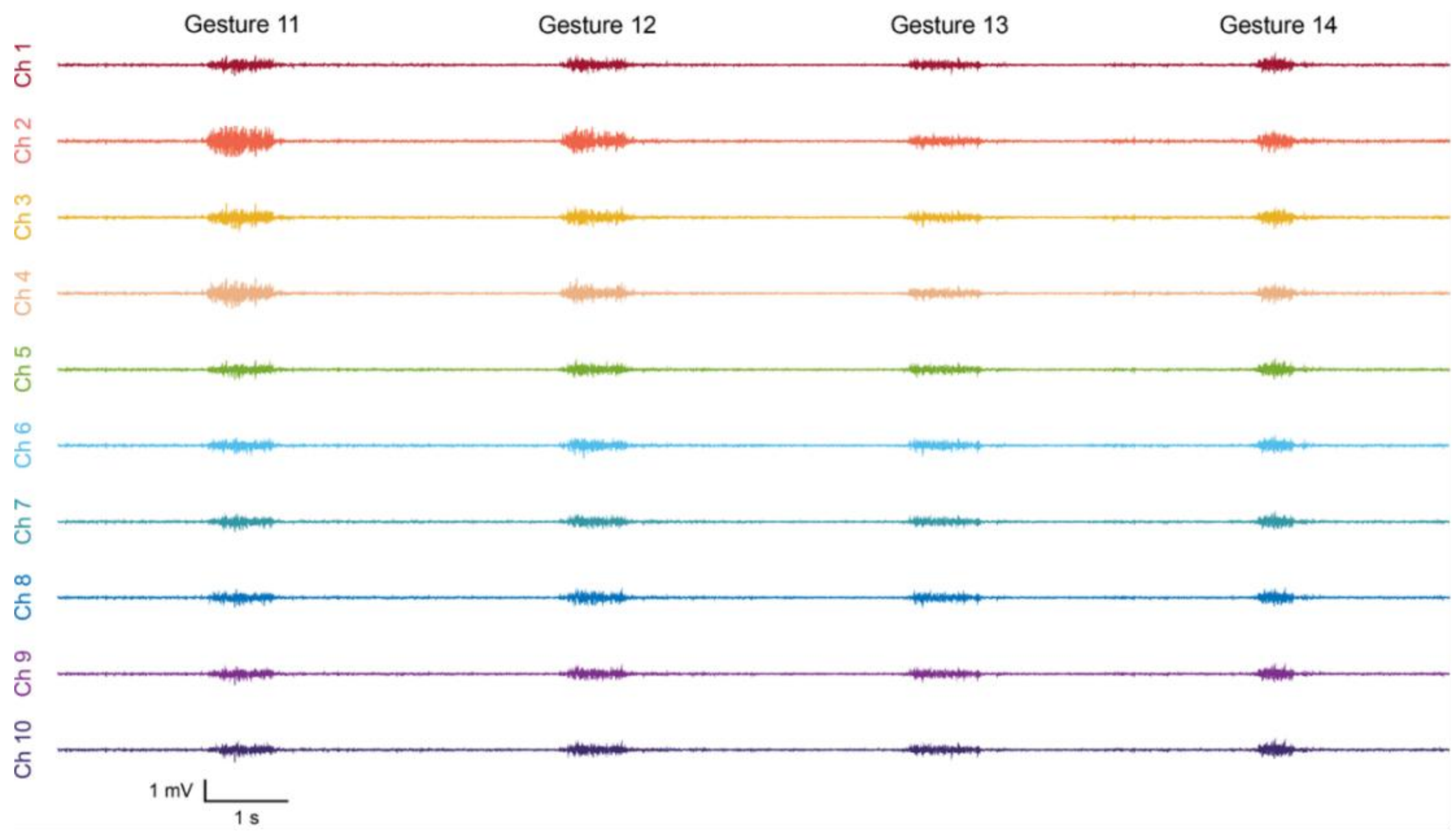
Gesture 11
Gesture 12
Gesture 13
Gesture 14
Ch 1
Ch 2
Ch 3
Ch 4
Ch 5
Ch 6
Ch 7
Ch 8
Ch 9
Ch 10
1 mV
1 s

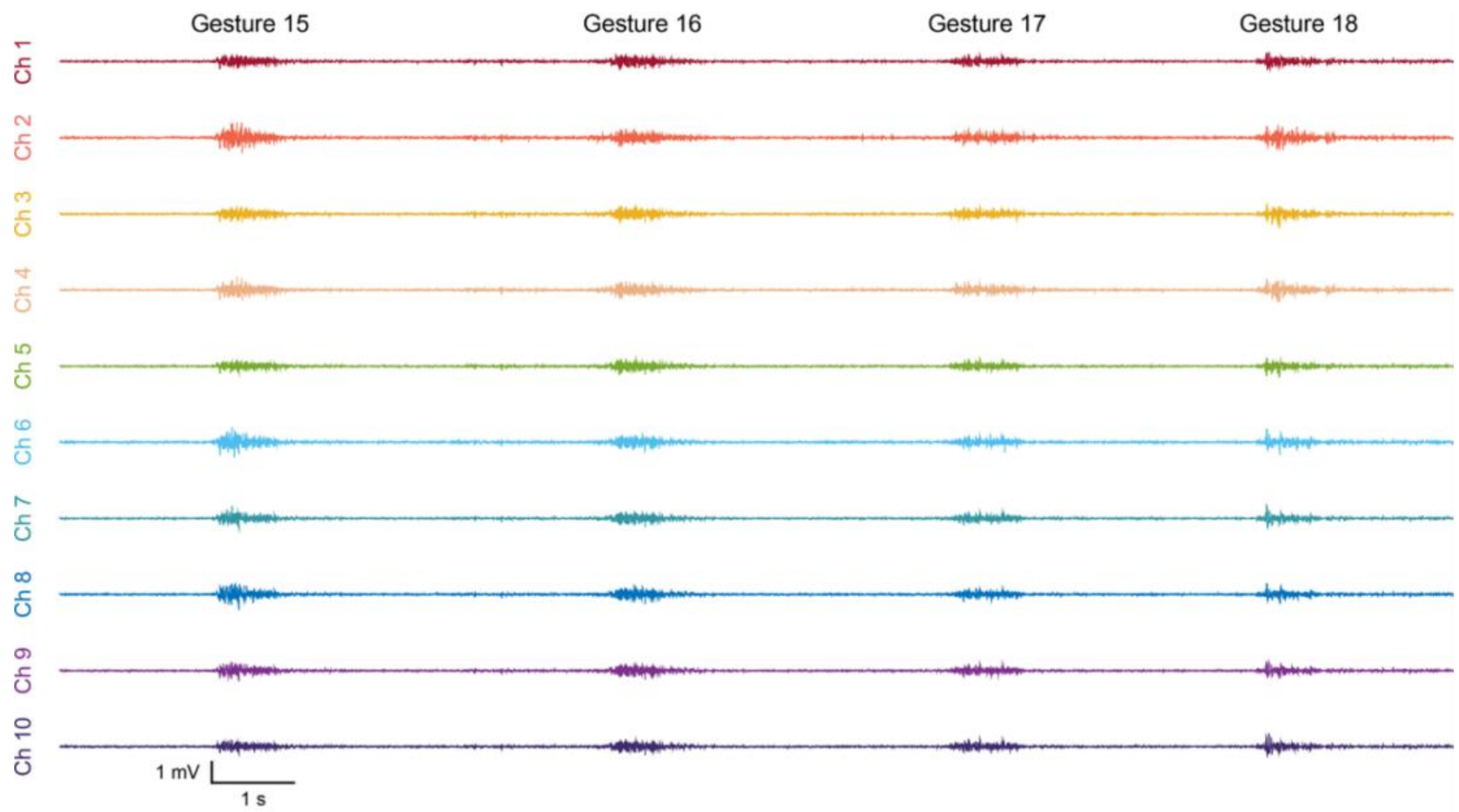
Gesture 15
Gesture 16
Gesture 17
Gesture 18
Ch 1
Ch 2
Ch 3
Ch 4
Ch 5
Ch 6
Ch 7
Ch 8
Ch 9
Ch 10
1 mV
1 s

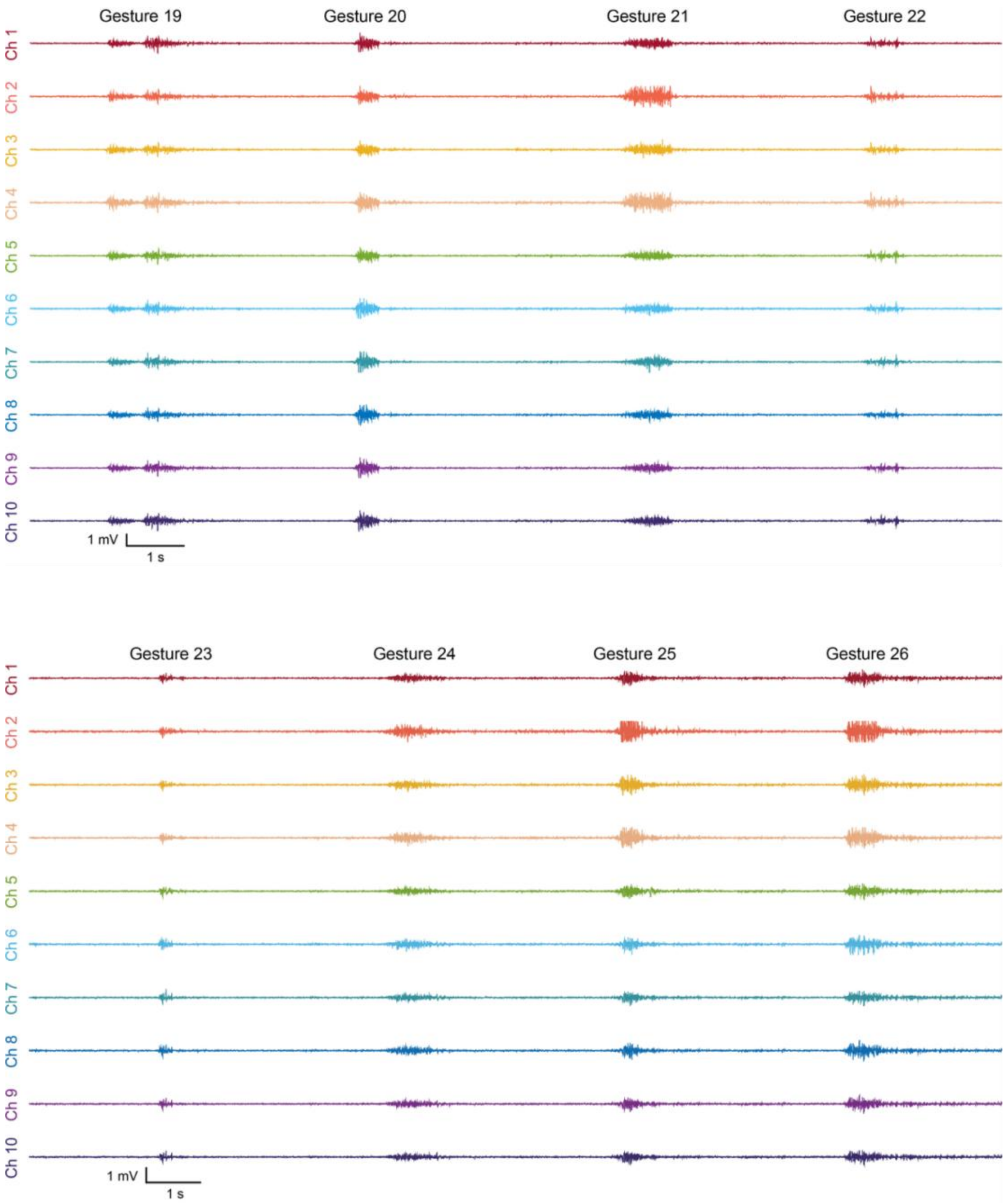

Supplementary Fig. 32. EMG signals of 26 tested gestures acquired by 10-channel hygrotrode array (gesture ID referring to table S4).

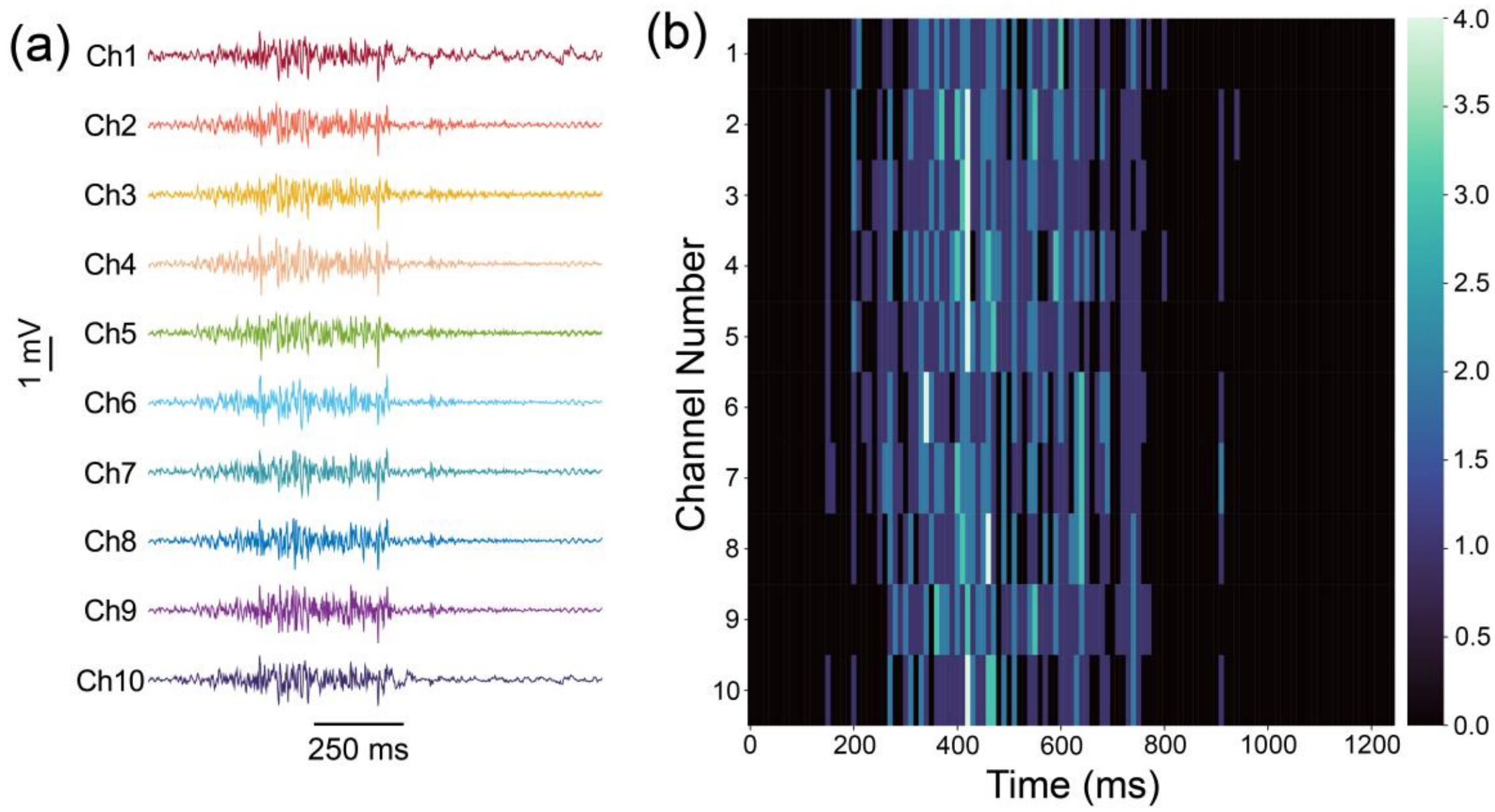

Supplementary Fig. 33. (a) 10-channel hygrotrode array-acquired sEMG data and (b) its Peri-Stimulus Time Histogram (PSTH) when fisting.

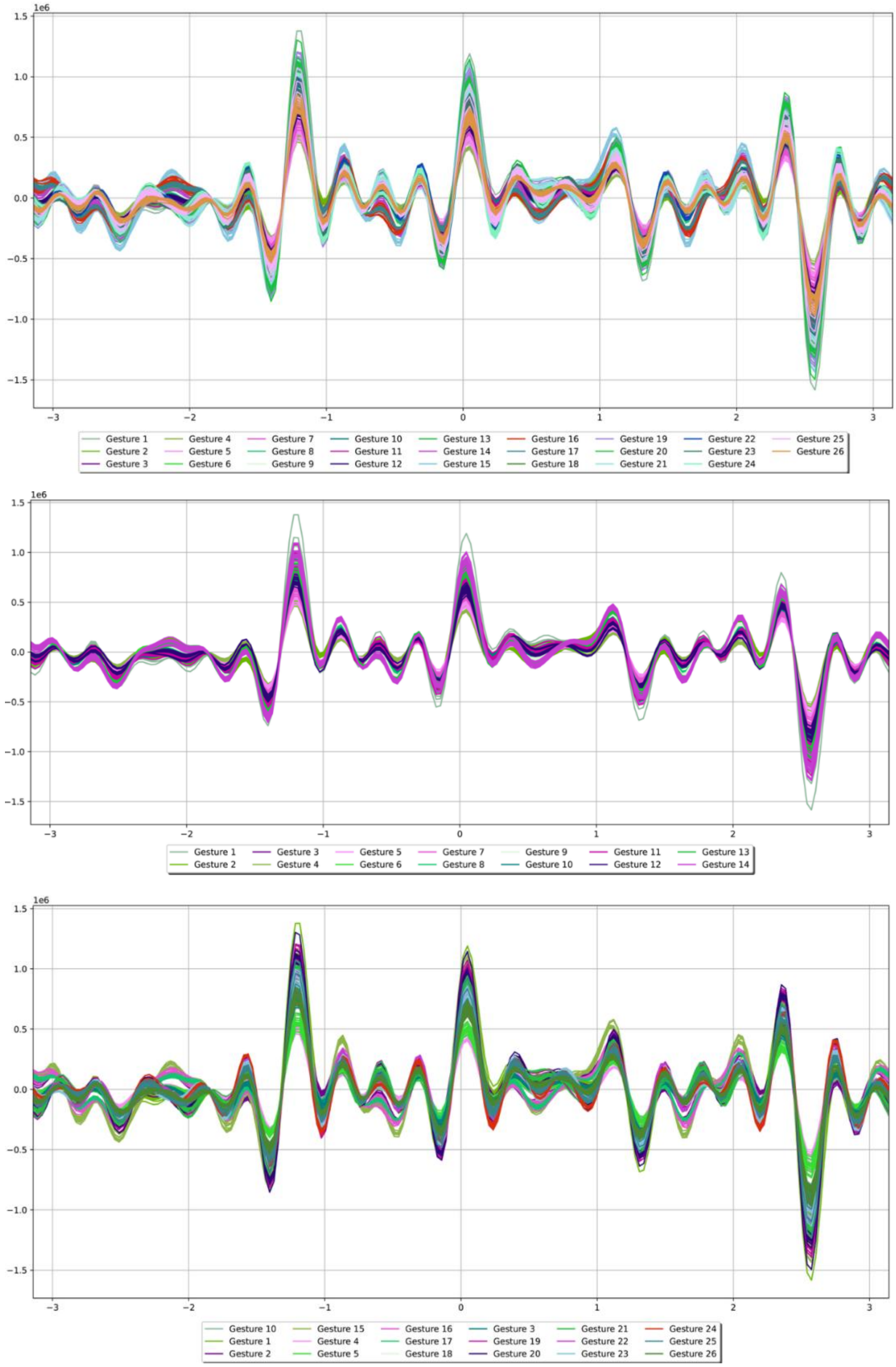

Supplementary Fig. 34. Andrew's curve for full dataset (prosthesis control + ASL gesture, top), group 1 (prosthesis control, middle) & group 2 (ASL gesture, bottom) (pandas API 1.3.4). Magnitude differences at peaks represent unique bio-signals in each gesture.

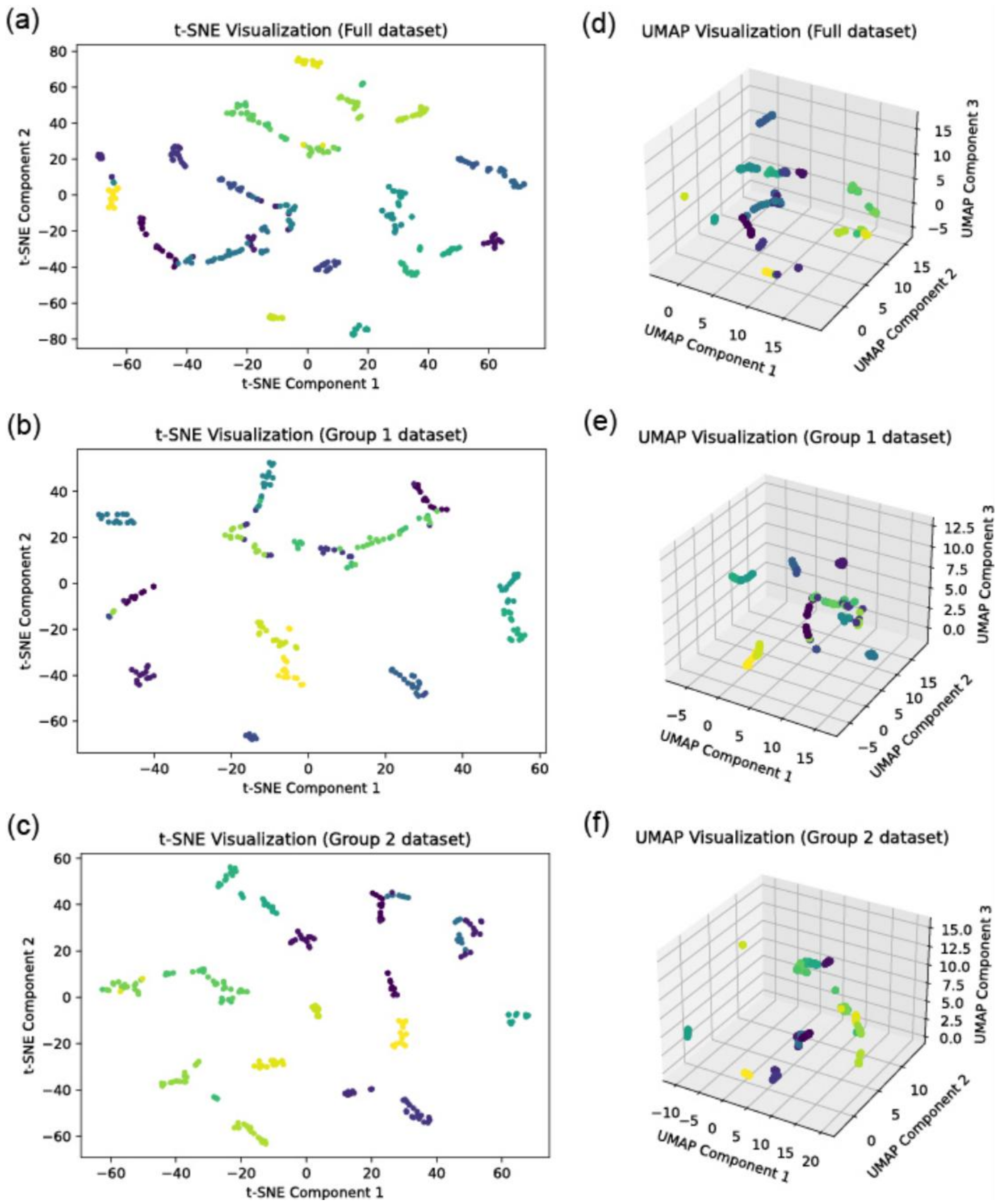

Supplementary Fig. 35. Data visualization after dimension reduction by t-distributed stochastic neighbor embedding (t-SNE) (scikit-learn API 0.24.2) and uniform manifold approximation and projection (UMAP) (umap-learn API 0.5.3) algorithms. (a-c) 2D data visualization by t-SNE. (d-f) 3D data visualization by UMAP. Clear clustering shows feasibility of these sEMG signals for gesture recognition.

(a)

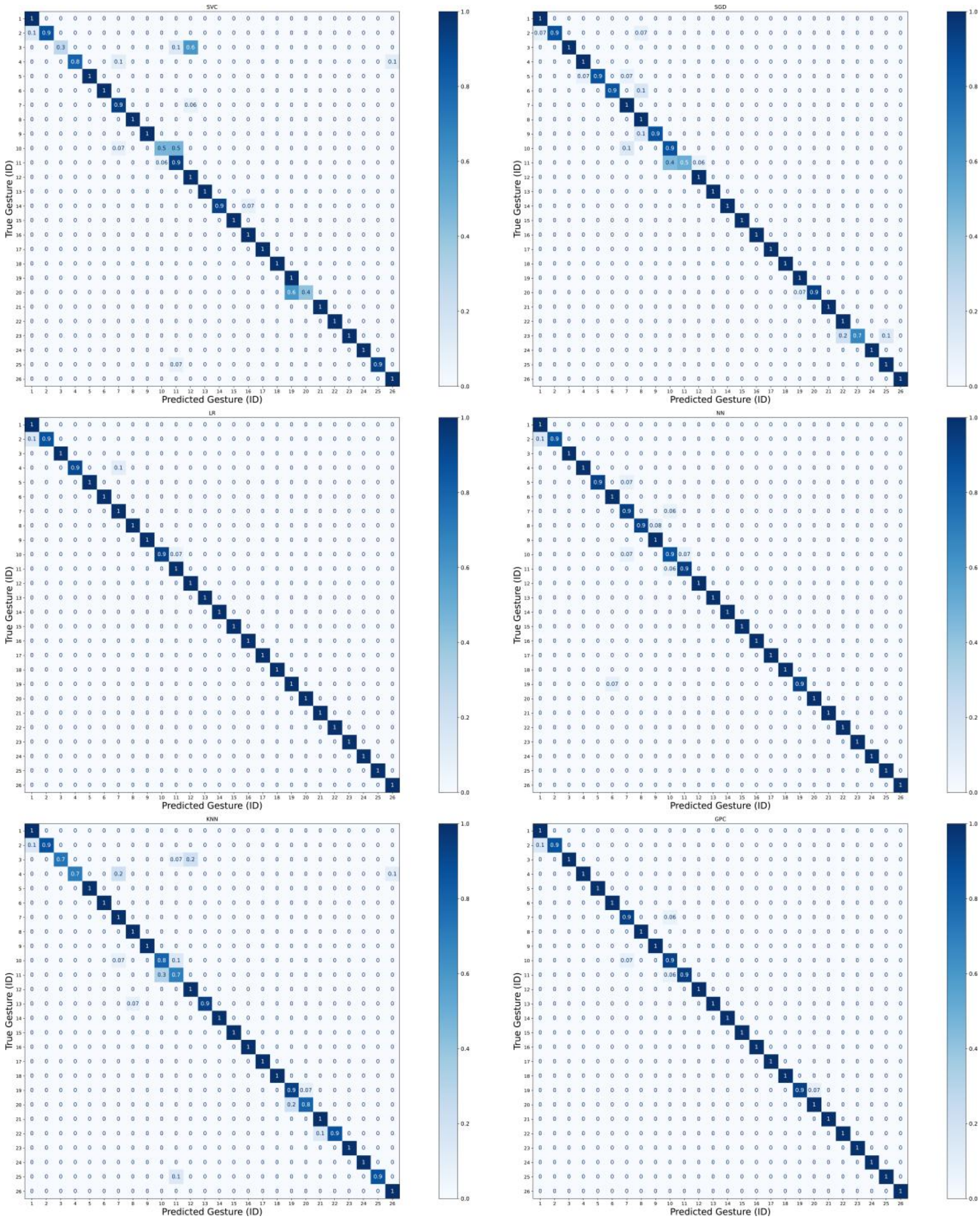

(b)

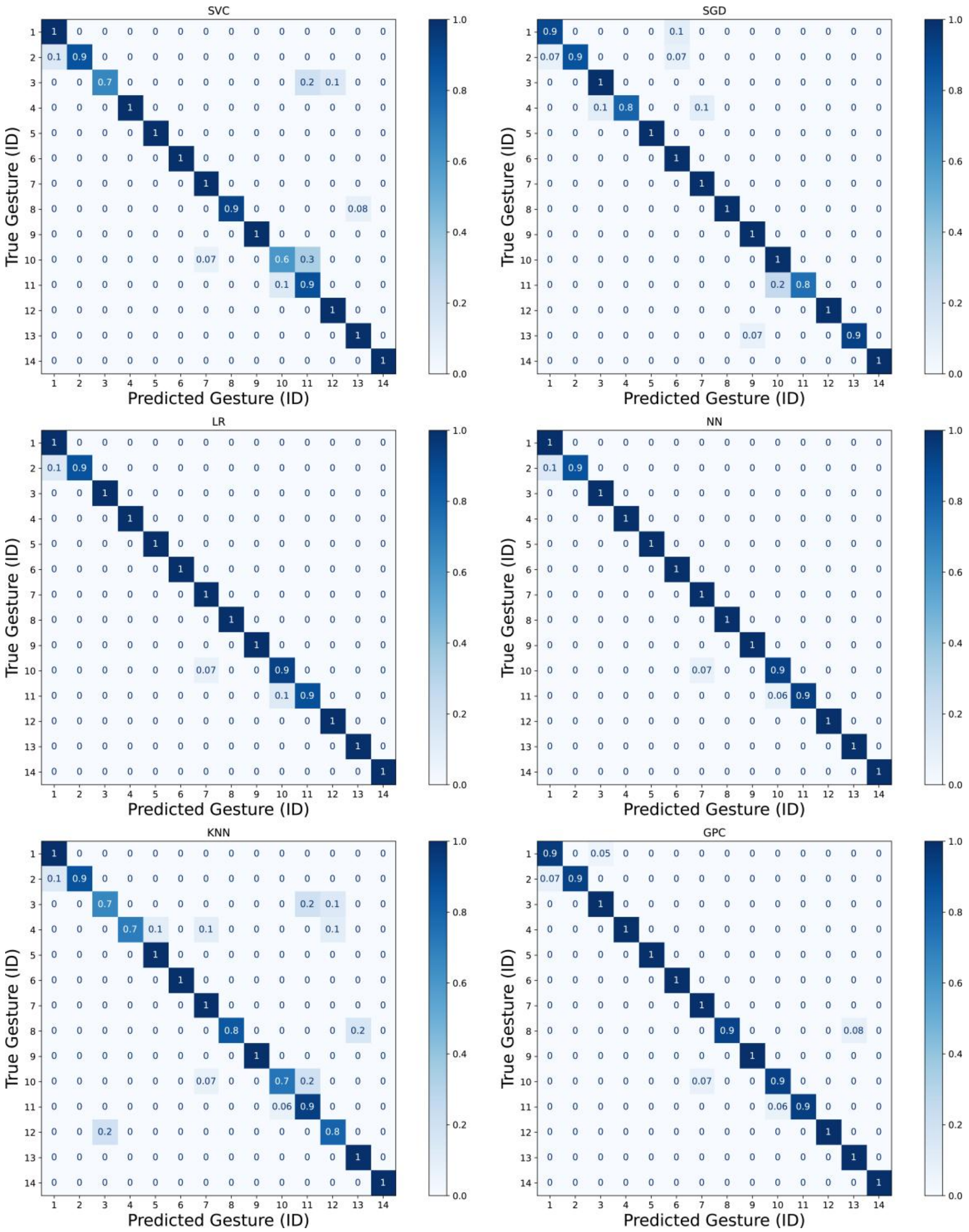

(c)

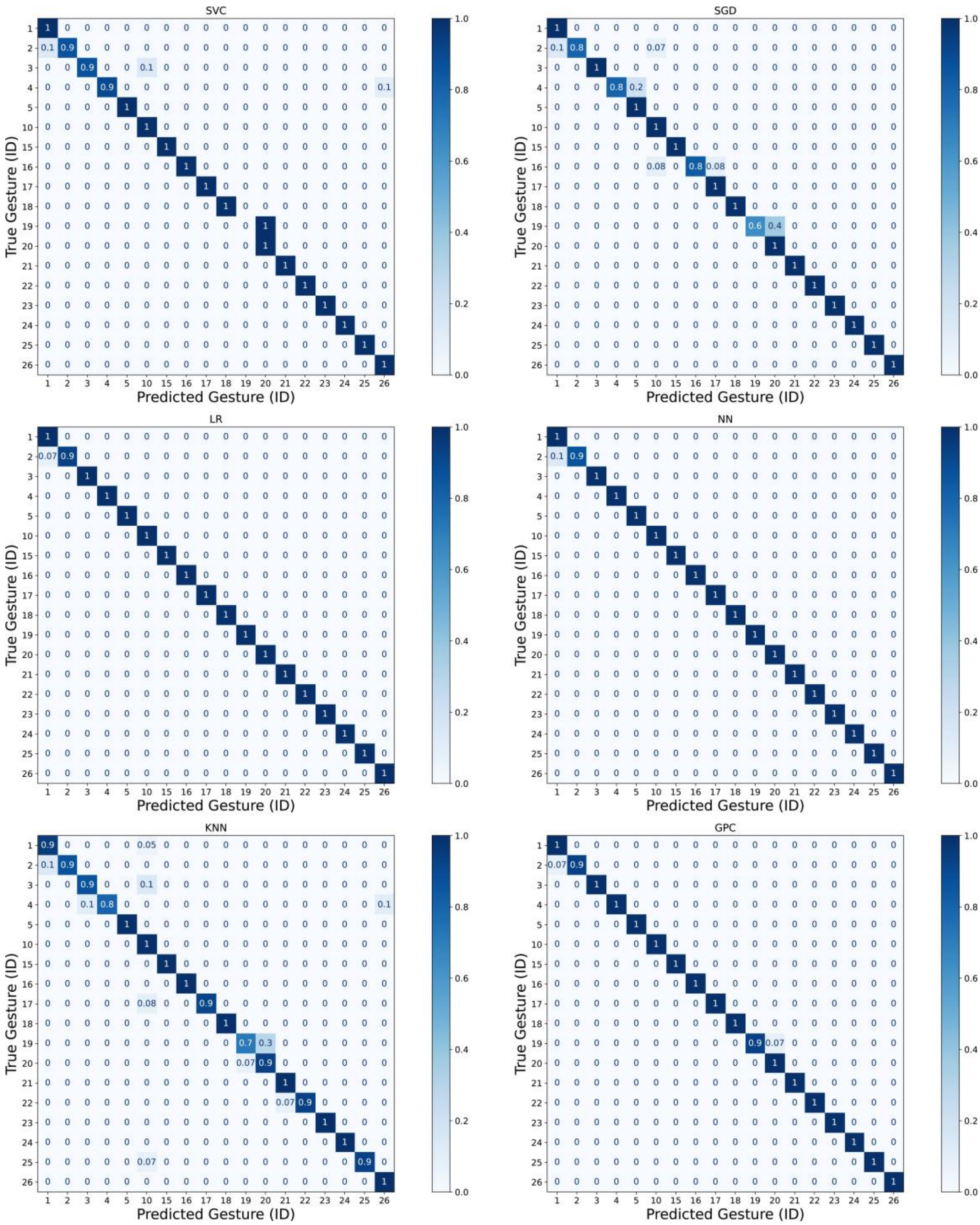

Supplementary Fig. 36. Confusion matrices of sEMG signals acquired by fresh hygrotrode. Confusion matrices for (a) 6 tested machine learning models (all gestures, prosthesis control & ASL gestures), (b) 6 tested machine learning models (group 1, prosthesis control gestures), (c) 6 tested machine learning models (group 2, ASL gestures).

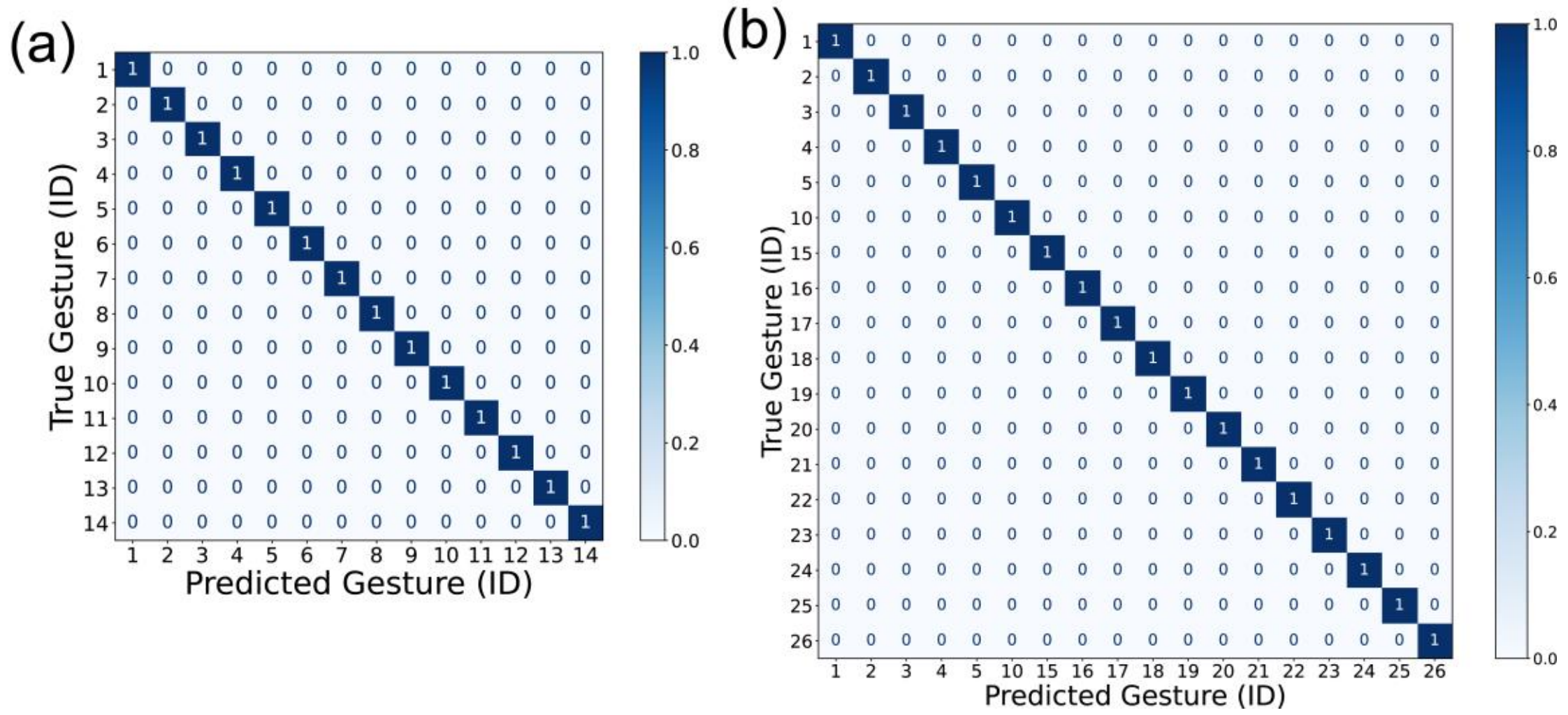

Supplementary Fig. 37. Confusion matrices of sEMG signals acquired by fresh hygrotrode after hyperparameter optimization. Confusion matrices for (a) optimized linear regression model (group 1, prosthesis control gestures) and (b) optimized linear regression model (group 2, ASL gestures).

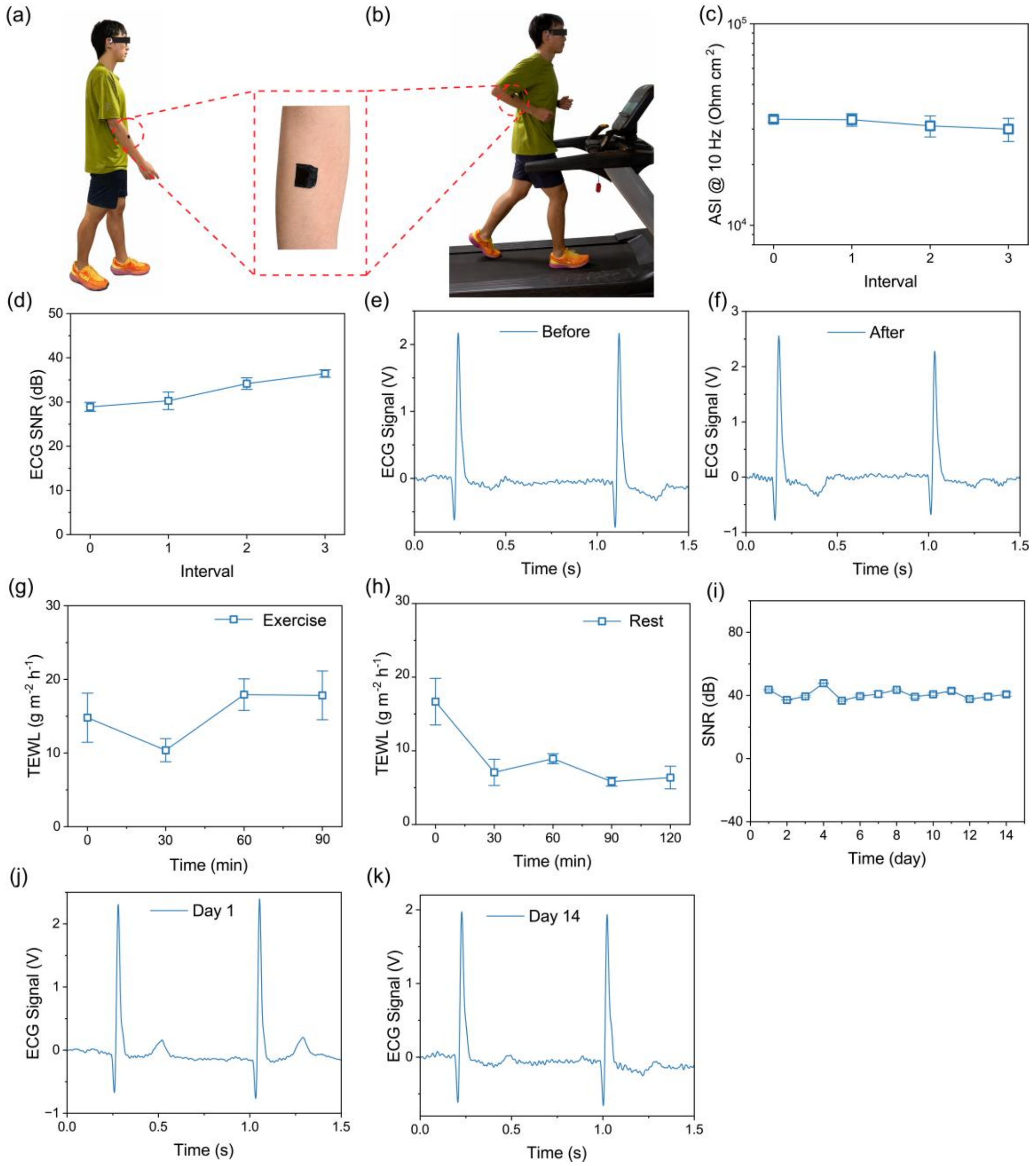

Supplementary Fig. 38 Electrical performance and TEWL change During 90-min exercise and 14-day continuous monitoring. (a, b) Representative images of the hygrotrode worn during walking and running. (c, d) ASI (at 10 Hz) and ECG SNR measured after intensive exercise intervals. (e, f) Representative ECG signals recorded before and after successive exercise intervals. (g) TEWL measured during continuous running exercise. (h) TEWL measured during rest condition. (i) ECG SNR change over time. (j, k) Acquired ECG signal on day 1 and day 14.

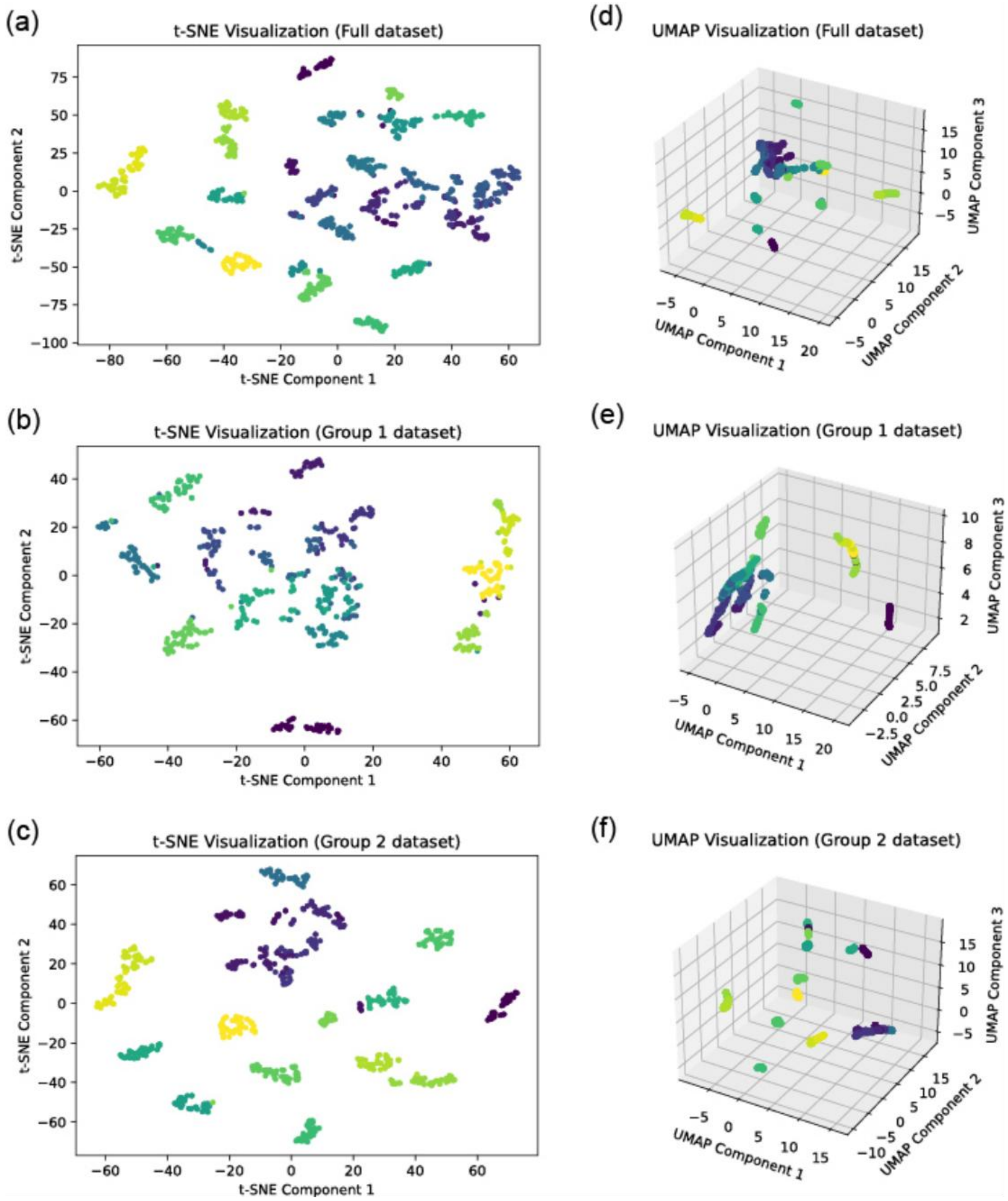

Supplementary Fig. 39. Visualization of data acquired by one-year dried hygrotrode array, after dimension reduction by t-distributed stochastic neighbor embedding (t-SNE) (scikit-learn API 0.24.2) and uniform manifold approximation and projection (UMAP) (umap-learn API 0.5.3) algorithms. (a-c) 2D data visualization by t-SNE. (d-f) 3D data visualization by UMAP. Clear clustering shows feasibility of these sEMG signals for gesture recognition.

(a)

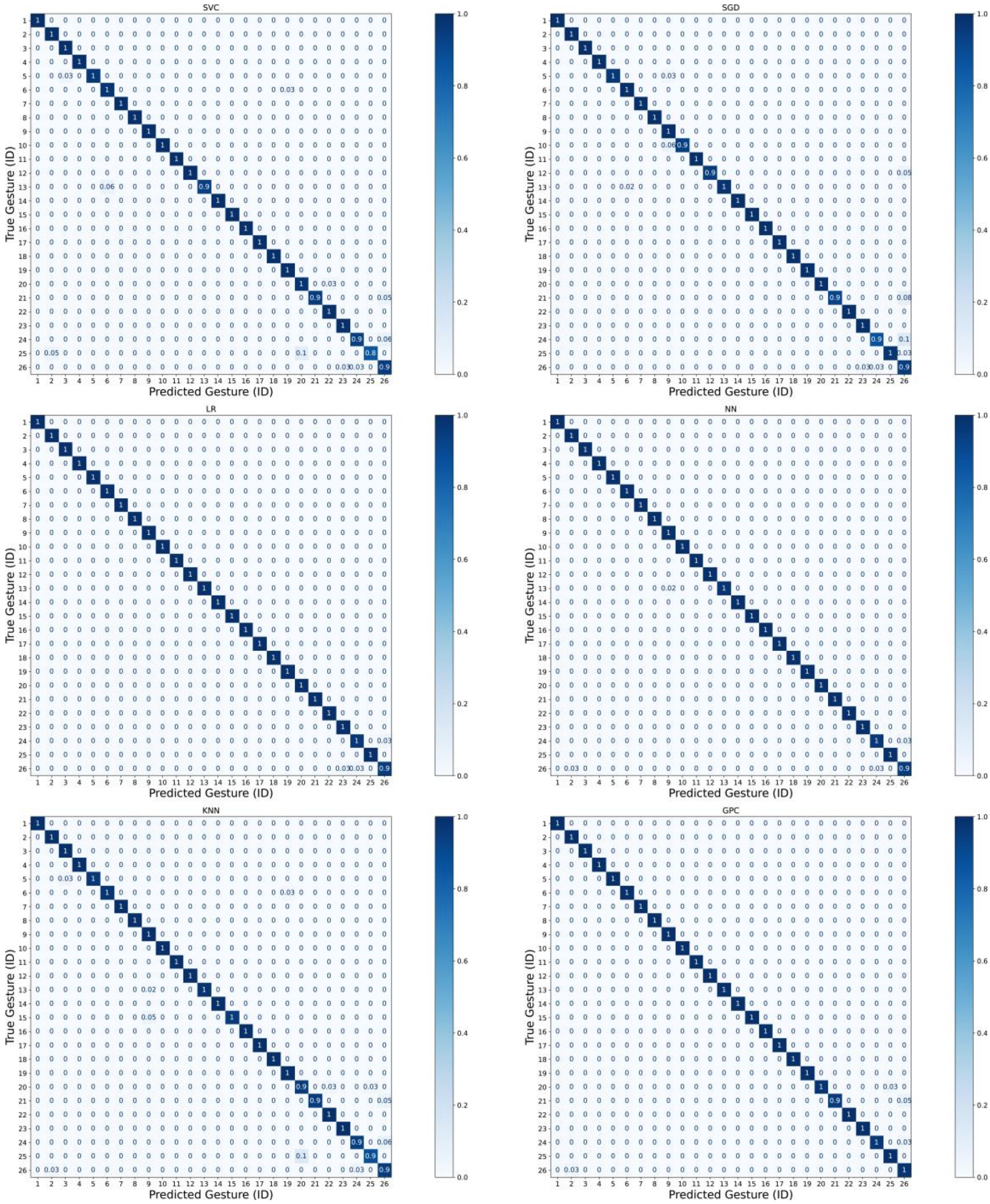

(b)

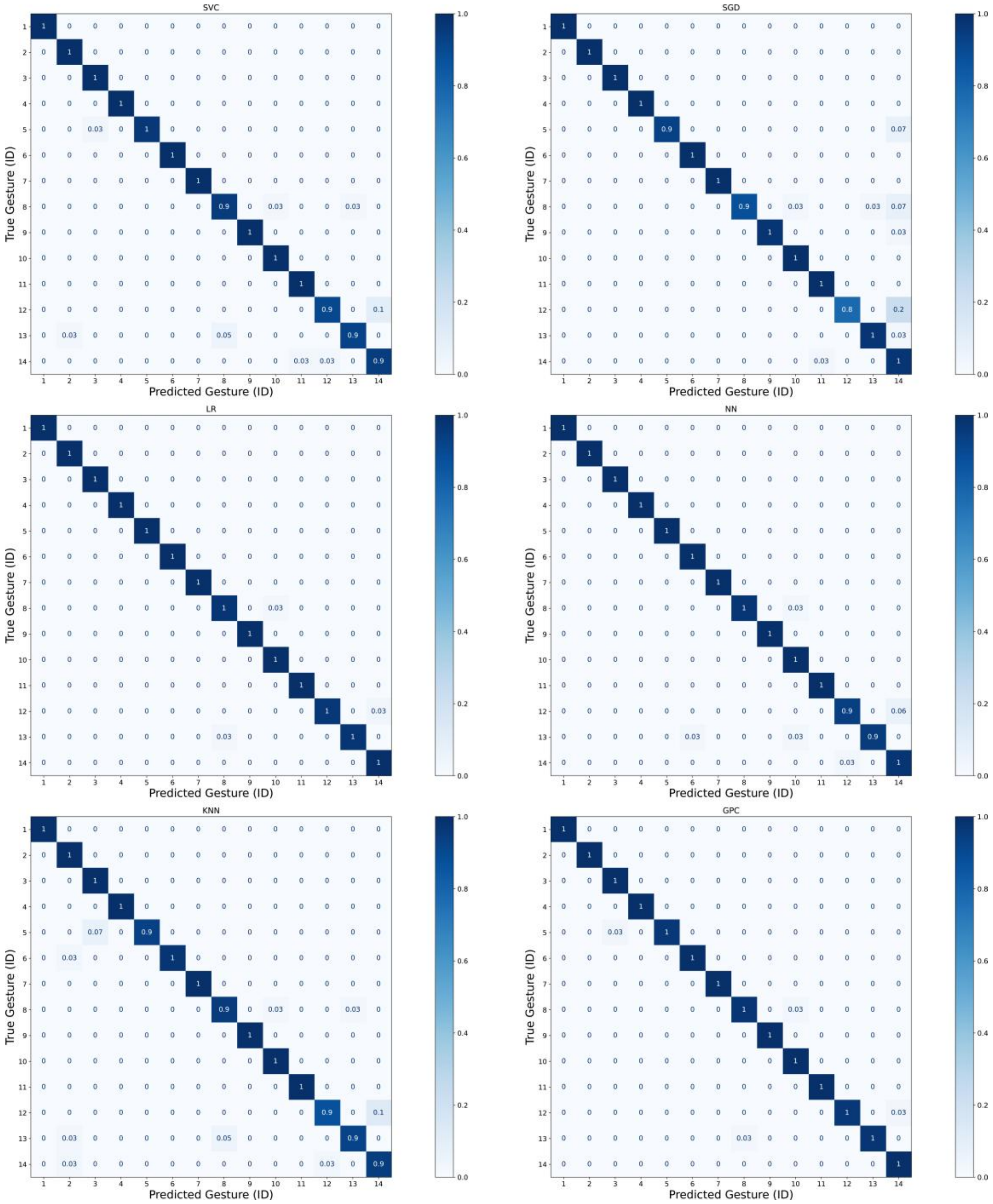

(c)

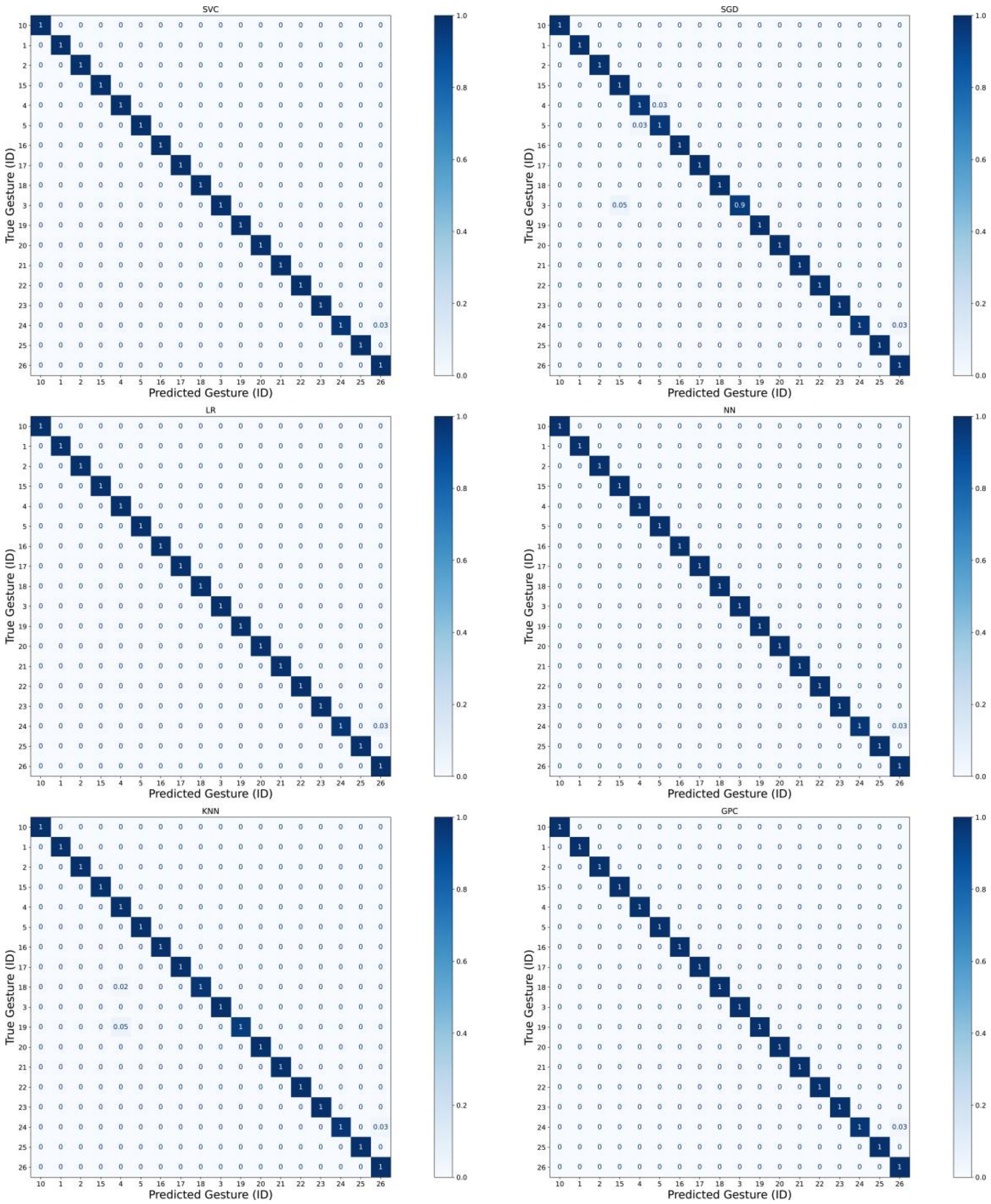

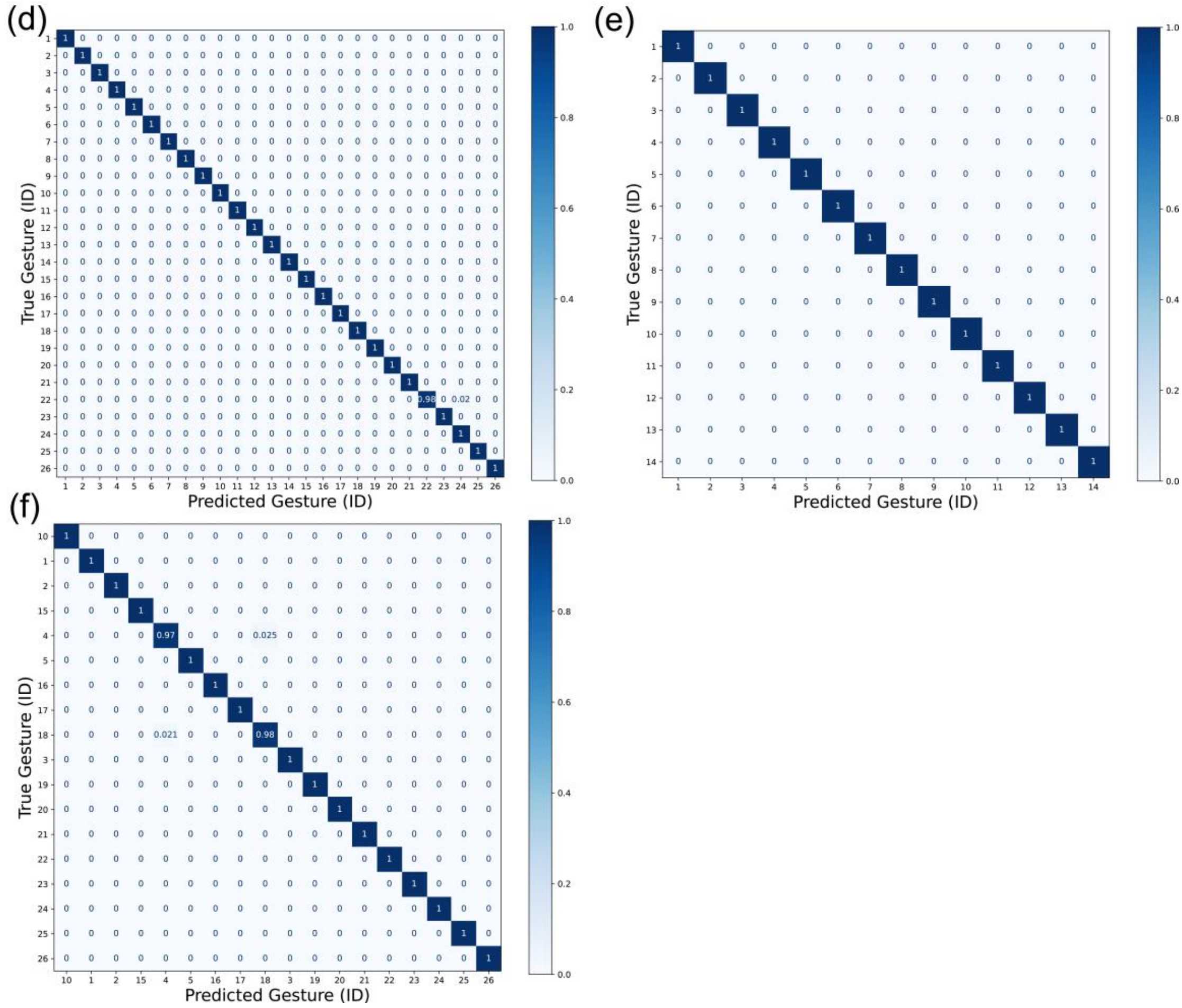

Supplementary Fig. 40. Confusion matrices of sEMG signals acquired by one-year dried hygrotrode. Confusion matrices for (a) 6 tested machine learning models (all gestures, prosthesis control & ASL gestures), (b) 6 tested machine learning models (group 1, prosthesis control gestures), (c) 6 tested machine learning models (group 2, ASL gestures), (d) optimized linear regression model (group 1, prosthesis control gestures) and (e) optimized linear regression model (group 2, ASL gestures).

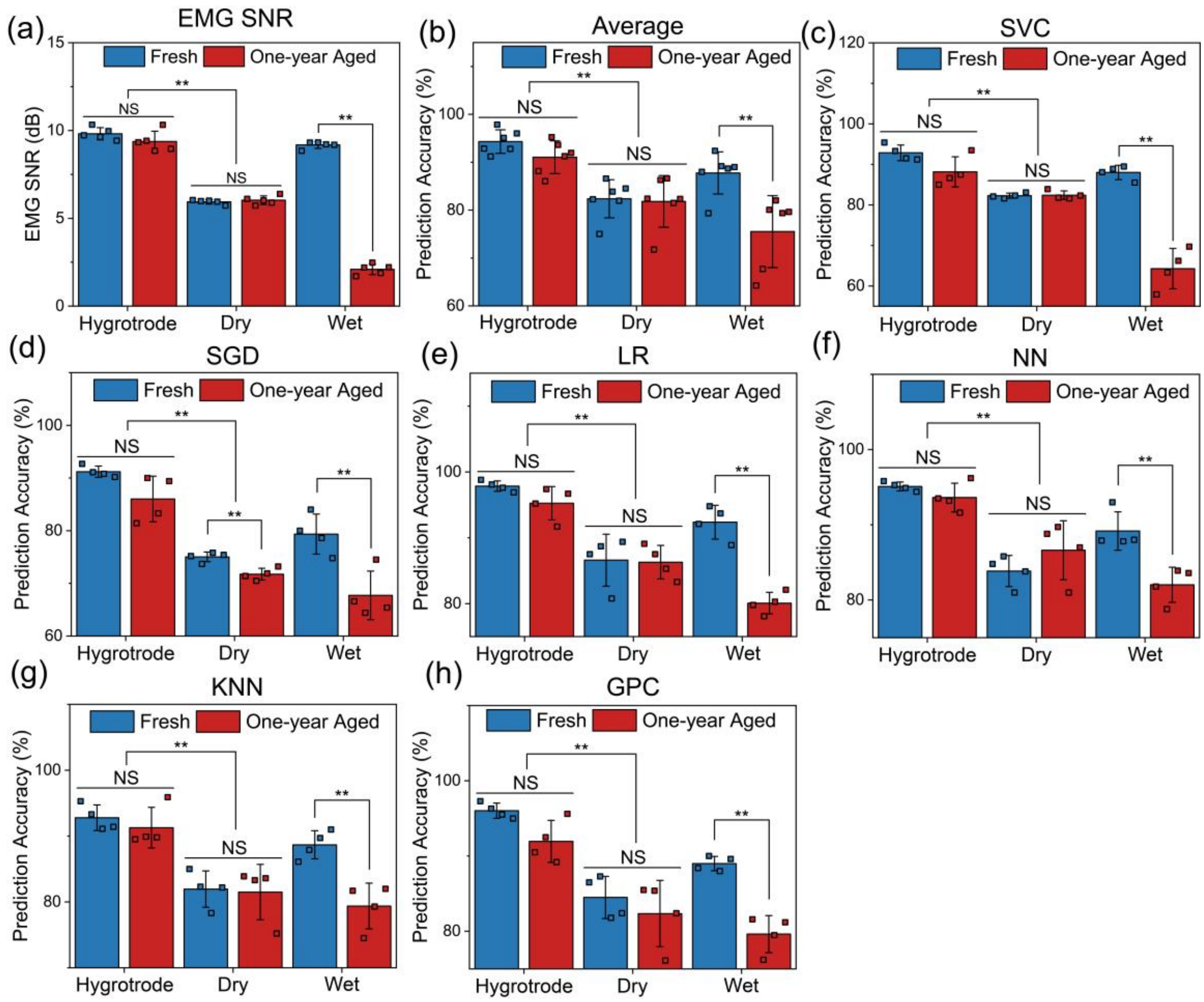

Supplementary Fig. 41. Comparison of (a) SNR of EMG signals (n=5), (b) average gesture prediction accuracy predicted by six models (n=6), gesture prediction accuracy predicted by (c) SVC, (d) SGD, (e) LR, (f) NN, (g) KNN and (h) GPC models, acquired by hygrotrode, metallic dry electrode and wet electrode (n=4). One-way ANOVA with Tukey post-hoc test: NS $P > 0.05$, $*P < 0.05$, $**P < 0.01$, $***P < 0.001$ versus control. All error bars denote the s.d. The P values for comparison of SNR are as follows: for sub-figure (a), for fresh hygrotrode versus one-year aged hygrotrode P = 0.19047, for fresh metallic electrode versus one-year aged metallic electrode P = 0.49954, for hygrotrode versus metallic electrode $P < 1\times10^{-4}$, for fresh wet electrode versus one-year aged wet electrode $P < 1\times10^{-4}$; for sub-figure (b), for fresh hygrotrode versus one-year aged hygrotrode P = 0.09126, for fresh metallic electrode versus one-year aged metallic electrode P = 0.84604, for hygrotrode versus metallic electrode $P = 8.62\times10^{-3}$, for fresh wet electrode

versus one-year aged wet electrode $P < 1\times10^{-4}$; for sub-figure (c), for fresh hygrotrode versus one-year aged hygrotrode $P = 0.08048$, for fresh metallic electrode versus one-year aged metallic electrode $P = 0.80522$, for hygrotrode versus metallic electrode $P = 3.58551\times10^{-4}$, for fresh wet electrode versus one-year aged wet electrode $P = 1.17\times10^{-3}$; for sub-figure (d), for fresh hygrotrode versus one-year aged hygrotrode $P = 0.09306$, for fresh metallic electrode versus one-year aged metallic electrode $P = 4.5\times10^{-3}$, for hygrotrode versus metallic electrode $P < 1\times10^{-4}$, for fresh wet electrode versus one-year aged wet electrode $P = 8.62\times10^{-3}$; for sub-figure (e), for fresh hygrotrode versus one-year aged hygrotrode $P = 0.13048$, for fresh metallic electrode versus one-year aged metallic electrode $P = 0.90307$, for hygrotrode versus metallic electrode $P < 1\times10^{-4}$, for fresh wet electrode versus one-year aged wet electrode $P = 4.28884\times10^{-4}$; for sub-figure (f), for fresh hygrotrode versus one-year aged hygrotrode $P = 0.22214$, for fresh metallic electrode versus one-year aged metallic electrode $P = 0.27048$, for hygrotrode versus metallic electrode $P < 1\times10^{-4}$, for fresh wet electrode versus one-year aged wet electrode $P = 6.25\times10^{-3}$; for sub-figure (g), for fresh hygrotrode versus one-year aged hygrotrode $P = 0.44806$, for fresh metallic electrode versus one-year aged metallic electrode $P = 0.8648$, for hygrotrode versus metallic electrode $P < 1\times10^{-4}$, for fresh wet electrode versus one-year aged wet electrode $P = 6.05\times10^{-3}$; for sub-figure (h), for fresh hygrotrode versus one-year aged hygrotrode $P = 0.05481$, for fresh metallic electrode versus one-year aged metallic electrode $P = 0.44721$, for hygrotrode versus metallic electrode $P < 1\times10^{-4}$, for fresh wet electrode versus one-year aged wet electrode $P < 1\times10^{-4}$.

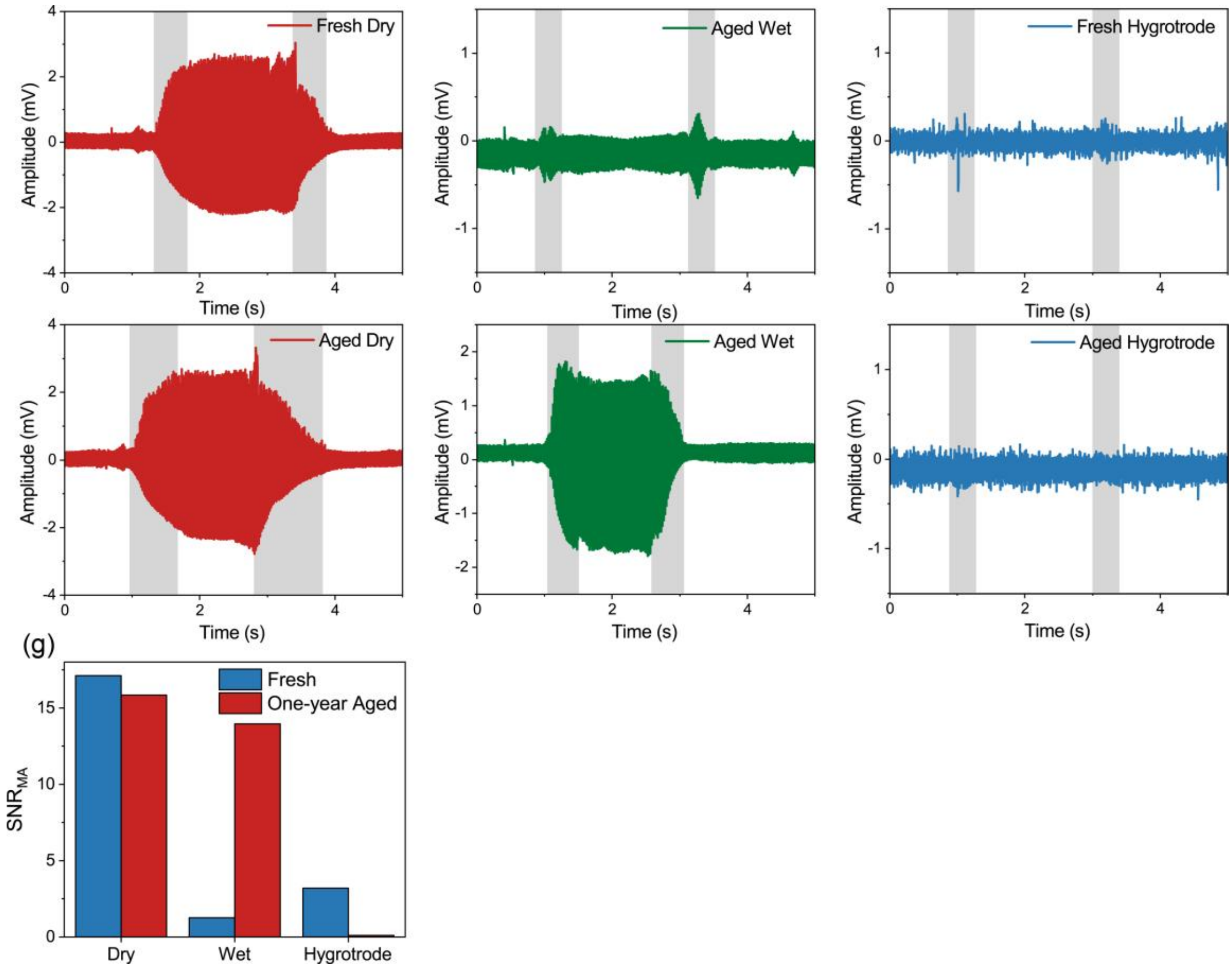

Supplementary Fig. 42. Motion artifact evaluation. EMG deviations were recorded by (a) fresh metallic electrode, (b) one-year dried metallic electrode, (c) fresh wet electrode, (d) one-year dried wet electrode, (e) fresh hygrotrode and (f) one-year dried hygrotrode. Shadow represents the application and removal of pressure. (g) Motion artifact comparison.

(a)

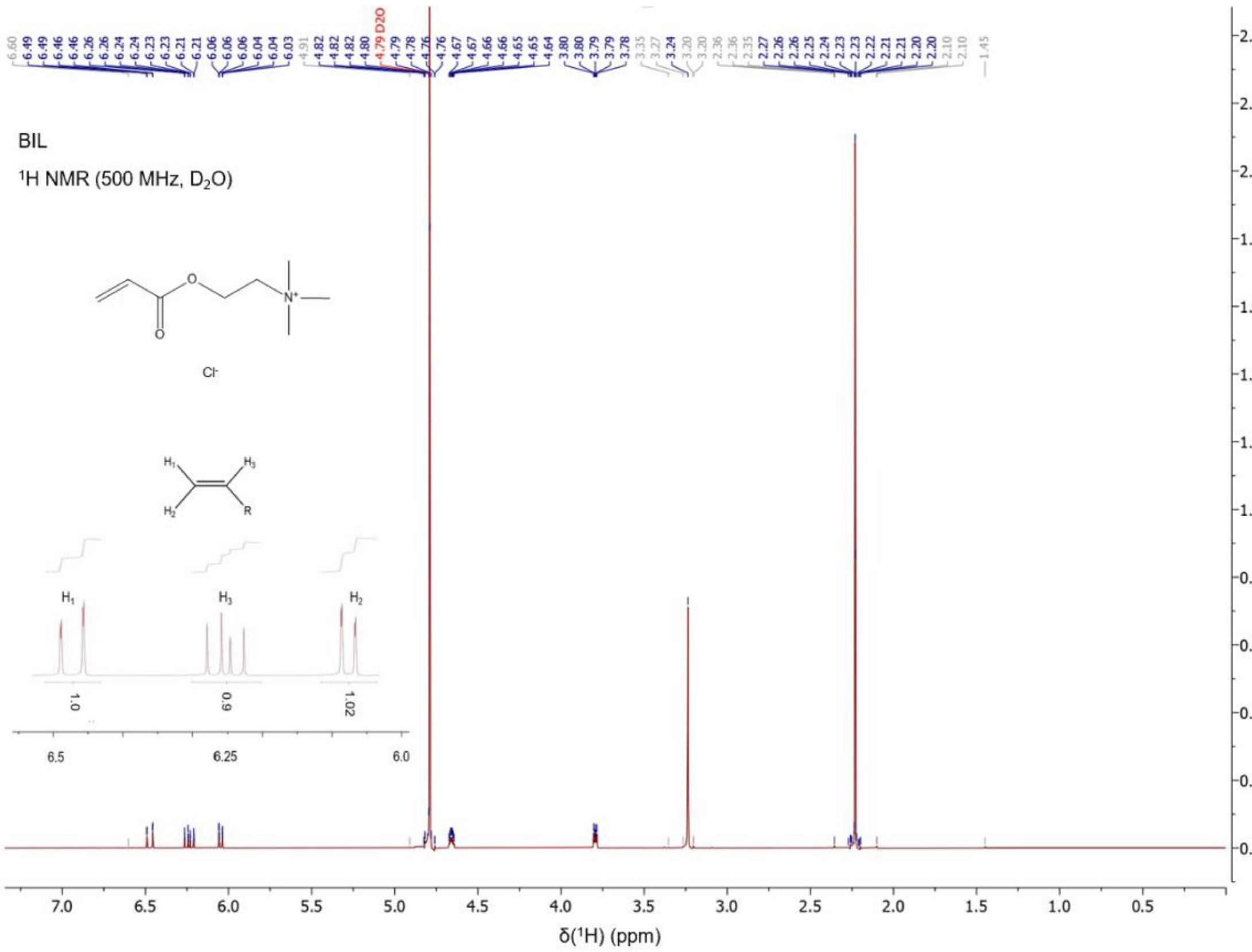
BIL
^1H NMR (500 MHz, D_2O)
Cl⁻
H_1
H_2
H_3
R
1.0
0.9
1.02
4.79 D2O
δ(^{1}H) (ppm)

(b)

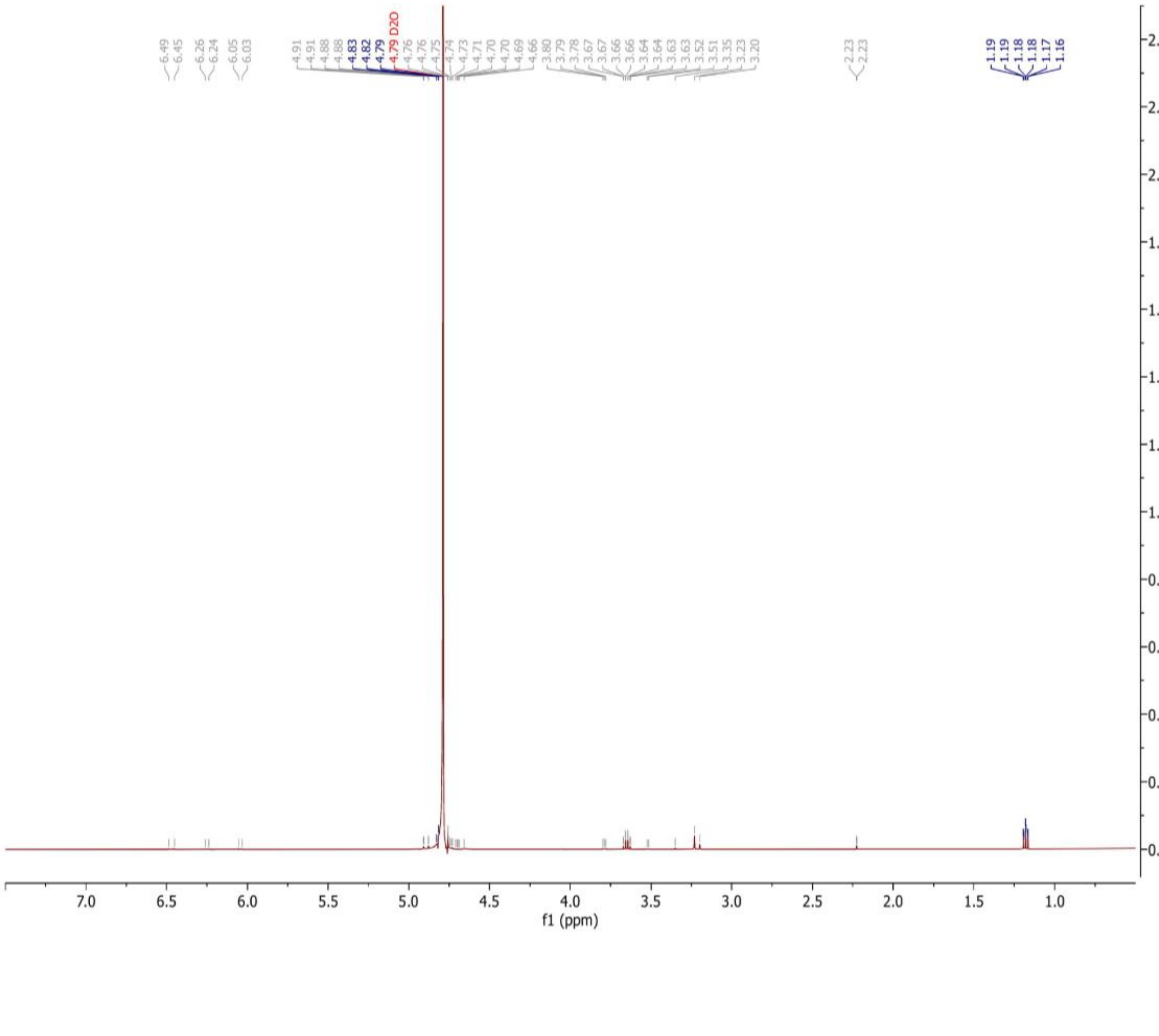
4.79 D2O
f1 (ppm)

(c)

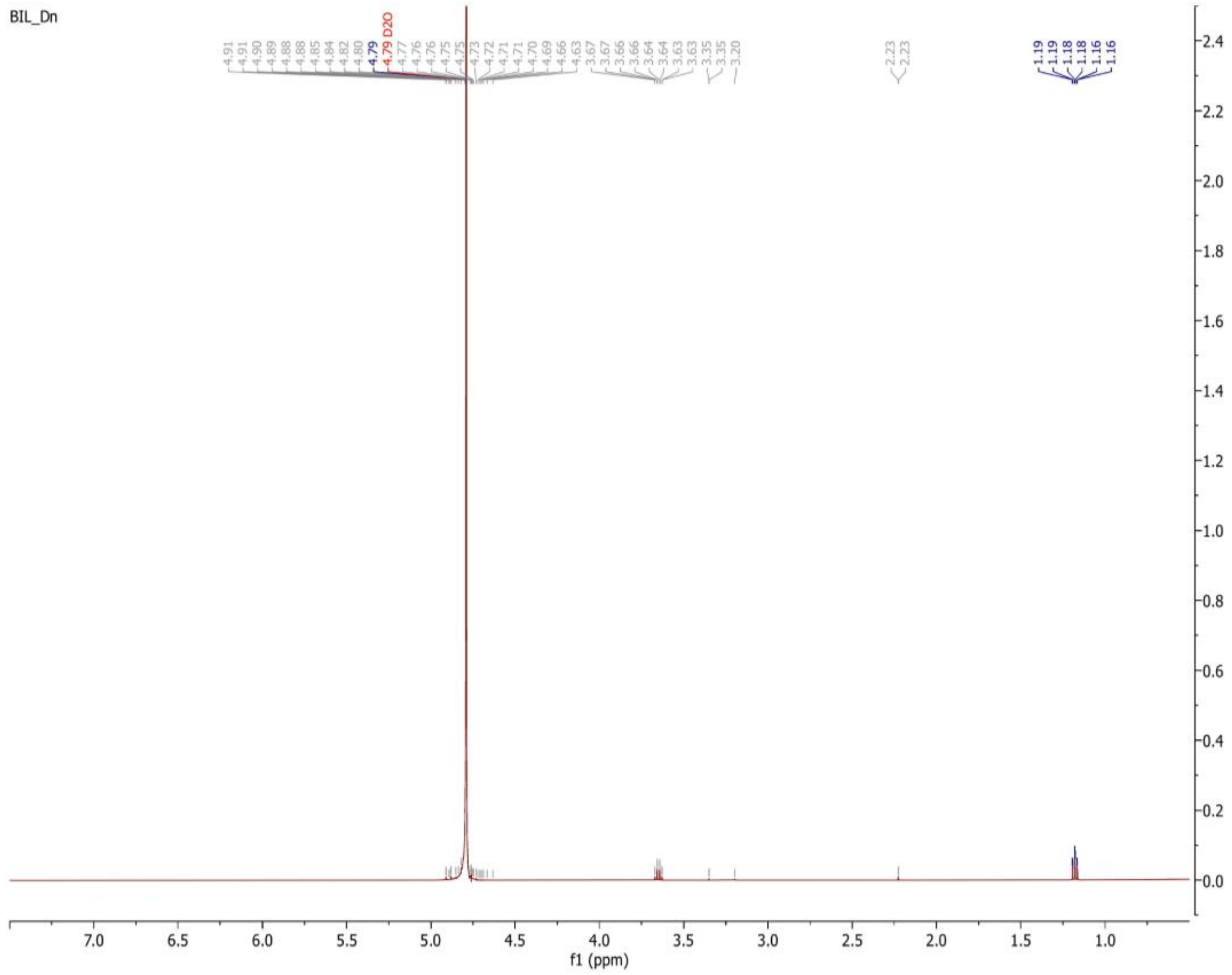

Supplementary Fig. 43. NMR Spectra. (a) ^{1}H NMR spectrum of the BIL monomer (80 wt% in H_2O), showing characteristic vinyl and alkyl proton resonances (6 ~ 6.5 ppm). (b) ^{1}H NMR spectrum of the D_2O soaking solution after overnight soaking of hygrotrode, indicating minimal residual monomer signals and relatively high degree of polymerization. (c) ^{1}H NMR spectrum of the D_2O soaking solution of hygrotrode after solvent exchange treatment, confirming the effective removal of unpolymerized monomers.

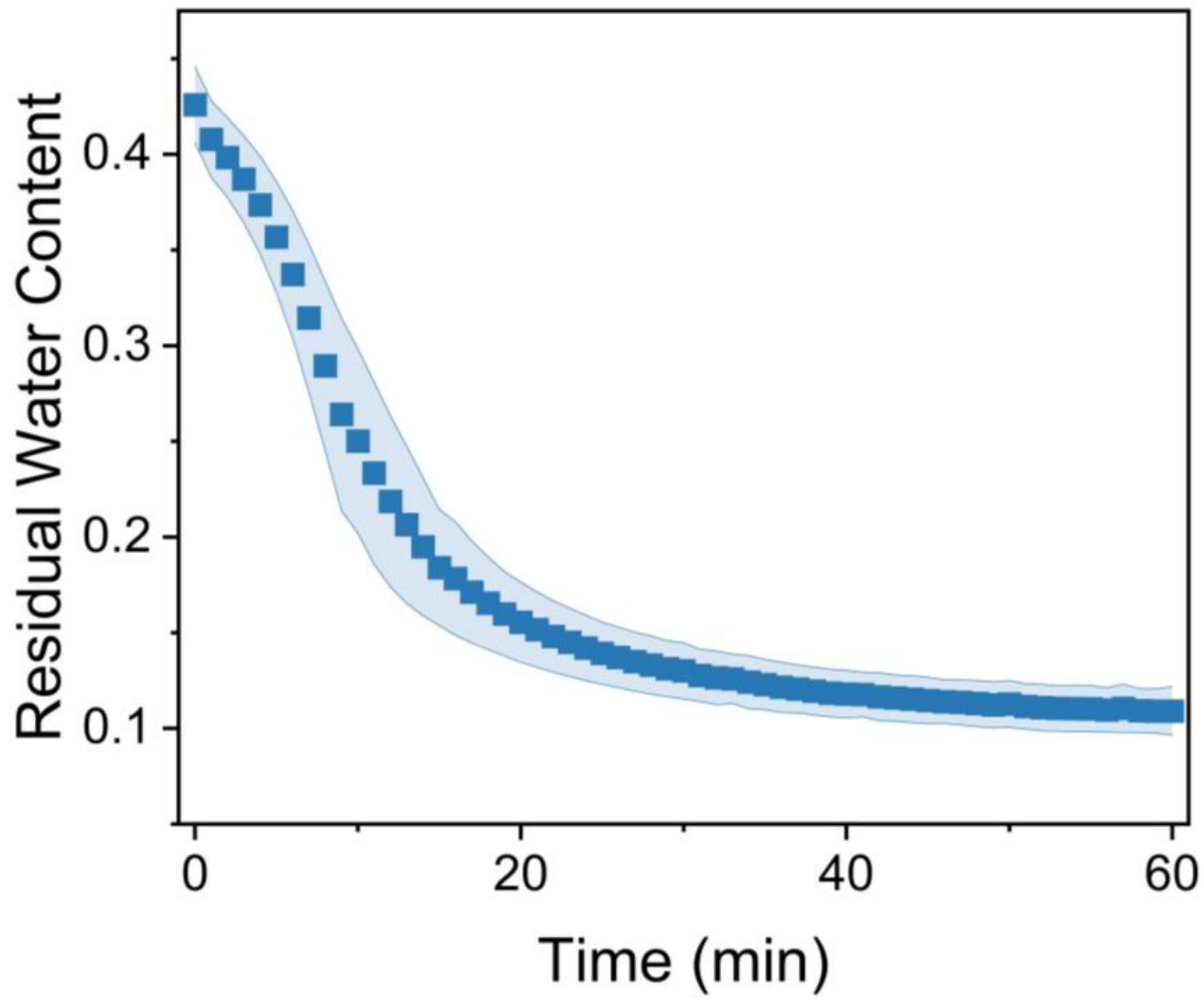

Supplementary Fig. 44 TGA curves of hygrotrode measured under nitrogen with a heating rate of 10 °C min^{-1}.

Supplementary Table 1. EIS fitting parameters of hygrotrode-skin & wet electrode-skin interface.

	Hygrotrode	Wet electrode
R_s (Ohm cm^2)	75.7	802
R_{ct} (Ohm cm^2)	10927	73304
W-R (Ohm cm^2)	2614	17034
W-Y_0 (S s^n cm^{-2})	7.033 * 10^{-5}	8.542 * 10^{-2}
W-η_0	0.5	0.5
CPE-Y_0 (10^{-7} S s^n cm^{-2})	2.069	1.019
CPE-η_0	0.814	0.863

Supplementary Table 2. Literature review of wet and dry electrodes for biopotential monitoring.

Reference	Electrode	Type	ASI at 10 Hz (kOhm cm2)	Monitoring Time

(*33*)	3M RedDot electrode	Wet	490	3 days
(*33*)	Natus adhesive electrode	Wet	169	1 day
(*34*)	Silk/PPy interlocked hydrogel	Wet	200	90 mins
(*35*)	Mxene/cellulose hydrogel	Wet	100	40 hours
(*36*)	Cholinium-based ionogel	Wet	20	72 hours
(*37*)	PVA/PAM hydrogel	Wet	40	1 day
(*38*)	PEDOT-PAM-PDA hydrogel	Wet	150	1 day
(*39*)	Mxene/HA hydrogel	Wet	100	12 hours
(*40*)	PEDOT:PSS-coated sponge	Dry	450	3 hours
(*41*)	Liquid metal-based composite	Dry	100	9 days
(*42*)	Ppy-based leather	Dry	170	12 hours
(*43*)	Au/PDMS/Zwitterionic polymer composite	Dry	300	18 hours
(*44*)	CNT/PDMS composite	Dry	7065	7 days
(*45*)	Conductive filler-knitted texture	Dry	1000	1 day
(*46*)	Silk/PEDOT:PSS/Glycerol composite film	Dry	1570	8 days
(*47*)	Silk/PTFE/AgNW film	Dry	177	6.875 days
(*48*)	Liquid metal composite	Dry	777.7	5 days
(*49*)	PEDOT:PSS/glycerol composite	Dry	120	1 day
(*50*)	PEDOT:PSS/PVA Composite	Dry	80	30 days
(*51*)	MWCNT/PDMS Composite	Dry	628	2 days
This work	Hygrotrode	Hygroscopic	20	30 days

Supplementary Table 3. Benchmarking dehydration strategies of epidermal electrode long-term physiological monitoring.

Strategies	Conductivity/ ASI	SNR/ Sensitivity	Use Time	Failure Mode/ Limitations
Encapsulation (*56–59*)	ASI @ 20 Hz: ~408 kOhm cm^2	EMG signal SNR: 19.8 dB	1-day continuous monitoring;<br>6 months storage time	Limited permerability during long-term

	ASI @ 100 Hz: ~78.5 kOhm cm^2	ECG signal: no obvious signal degradation	8-hour continuous monitoring	wear, may leading to skin irritation
	ASI @ 1000 Hz: ~15 kOhm cm^2	ECG signal SNR: 38.38 dB	14 day continuous monitoring	
	ASI @ 1000 Hz: ~3 kOhm cm^2	ECG signal SNR: 4.5 dB	10-min continuous monitoring	
Zwitterionic-based Hydrogel (*60–63*)	ASI @ 10 Hz: ~290 kOhm cm^2	ECG signal SNR: 35 dB	1-day continuous monitoring	Water-content-dependent ionic conductivity
	Conductivity: 3 S m^{-1}	ECG sensitivity: 0.46 (the ratio of the T-wave to the R-wave voltage)	35-day storage time	
	Conductivity: 1.05 S m^{-1}	ECG signal: no obvious signal degradation	1-day continuous monitoring	
	Conductivity: 0.5 S m^{-1}	ECG signal: no obvious signal degradation	12-day storage time	
Ionic/ salt Additive (*64–68*)	ASI @ 10 Hz: ~1470 kOhm cm^2	ECG signal SNR: 35.1 dB	8-day continuous monitoring	Potential leakage of salt ions and its bad biocompatibility
	ASI @ 10 Hz: ~55 kOhm cm^2	ECG signal SNR: 33.21 dB	30-min continuous monitoring	

	Conductivity: 1.04 S m^{-1}	ECG signal: no obvious signal degradation	30-day storage time	
	Conductivity: 1.78 S m^{-1}	ECG & EMG signal: no obvious signal degradation	6-day continuous monitoring	
	ASI @ 10 Hz: ~24 kOhm cm^2	ECG signal SNR: 25 dB	5-hour continuous monitoring	
Glycerol-based Organogel (*69–73*)	ASI @ 10 Hz: ~34.75 kOhm cm^2	ECG signal SNR: 36.7 dB	3-day continuous monitoring	Potential leakage of glyrecol that may lead to signal degradation
	ASI @ 10 Hz: ~140 kOhm cm^2	EEG signal SNR: 20 dB	12-hour continuous monitoring	
	ASI @ 10 Hz: ~30 kOhm cm^2	EEG signal SNR: 18.91 dB	8-hour continuous monitoring	
	ASI @ 10 Hz: ~250 kOhm cm^2	ECG signal: no obvious signal degradation	8-hour continuous monitoring; 25-day storage time	
	ASI @ 10 Hz: ~20 kOhm cm^2	EEG signal SNR: 25.2 dB	24-hour continuous monitoring	
Ionic Liquid-based Organogel (*74–78*)	ASI @ 10 Hz: ~700 kOhm cm^2	EMG signal SNR: 30 dB	Two-month storage time	Potential leakage of ionic liquid that may lead to signal degradation and biotoxicity issue
	ASI @ 10 Hz: ~196.3 kOhm cm^2	EMG signal SNR: 12 dB;	Two-week storage time	

		ECG signal SNR: 25 dB		
	ASI @ 10 Hz: ~100 kOhm cm^2	ECG signal SNR: 16.3 dB	3-day continuous monitoring; one-month storage time	
	ASI @ 10 Hz: ~250 kOhm cm^2	ECG signal: no obvious signal degradation	3-day continuous monitoring	
	ASI @ 10 Hz: ~20 kOhm cm^2	ECG signal SNR: ~16 dB	3-day continuous monitoring	
Thin film (*13*, *79–82*)	ASI @ 10 Hz: ~1050 kOhm cm^2	ECG signal SNR: 32.8 dB	8 days continuous monitoring	Potential leakage of unanchored hygroscopic species that may lead to signal degradation and biotoxicity issue
	ASI @ 10 Hz: ~157 kOhm cm^2	EMG signal SNR: 48.8	12-hour continuous monitoring	
	ASI @ 100 Hz: ~10.6 kOhm cm^2	ECG signal SNR: 25.3 dB; EMG signal SNR: 13.8 dB	13-day continuous monitoring	
	ASI @ 100 Hz: ~100 kOhm cm^2	ECG signal: no obvious signal degradation	24-hour continuous monitoring	
	ASI @ 100 Hz: ~100 kOhm cm^2	ECG signal SNR: 6.41 dB	24-hour continuous monitoring	

Hygrotrode	ASI @ 10 Hz:<br>107 kOhm cm^2	ECG signal<br>SNR: 37.2 dB	14-day continuous monitoring; one-year storage time	

Supplementary Table 4. Gesture ID and corresponding pictures (CNG: Chinese number gesture)

ID	Gesture	Pictures	ID	Gesture	Pictures
1	CNG 1;<br>ASL “1”	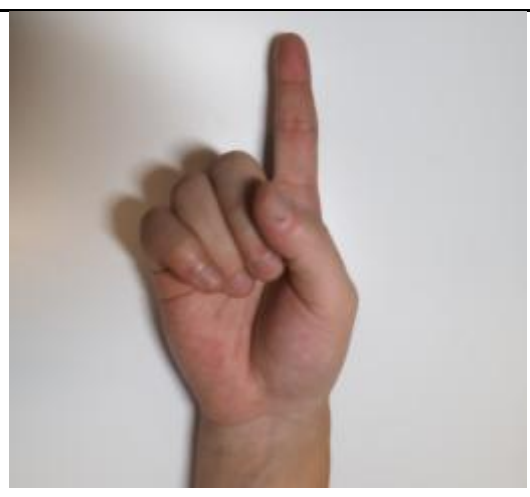	14	Gesture “wrist extension”	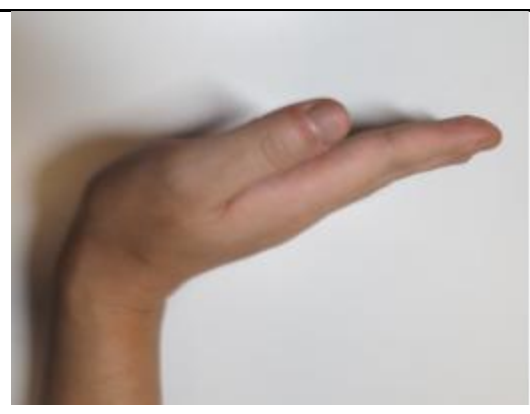
2	CNG 2;<br>ASL “2”	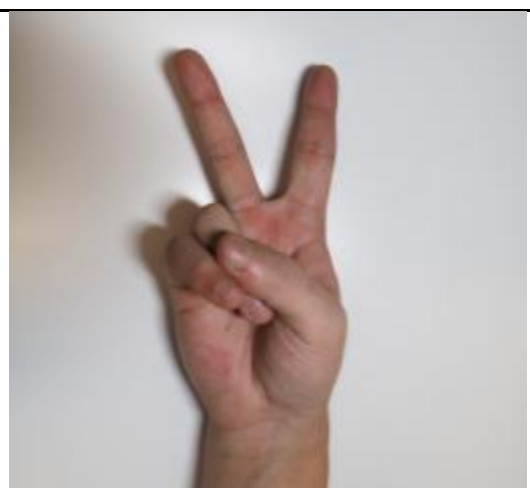	15	ASL “3”	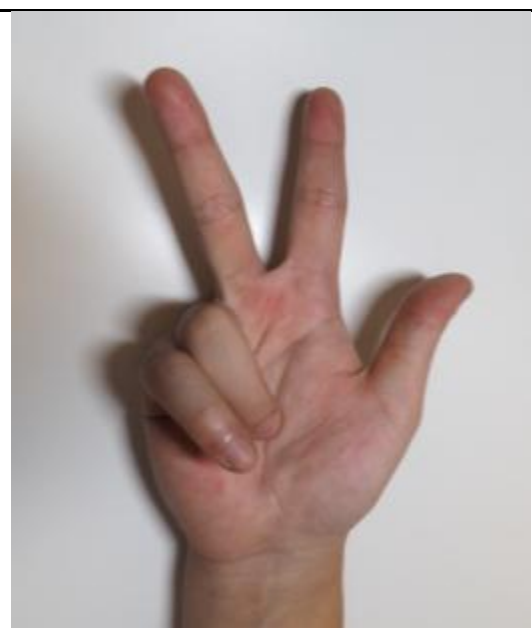
3	CNG 3;<br>ASL “9”	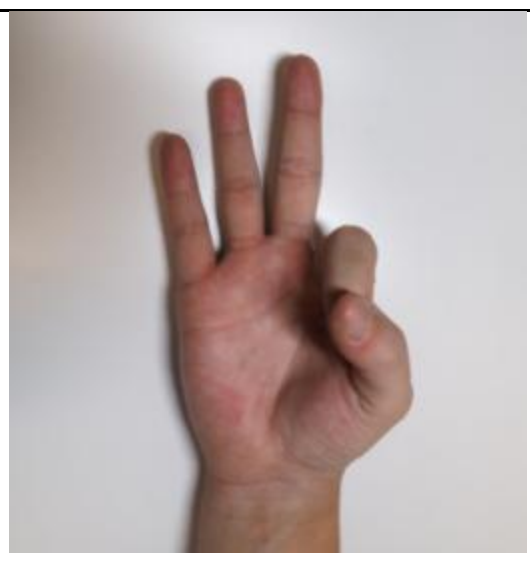	16	ASL “6”	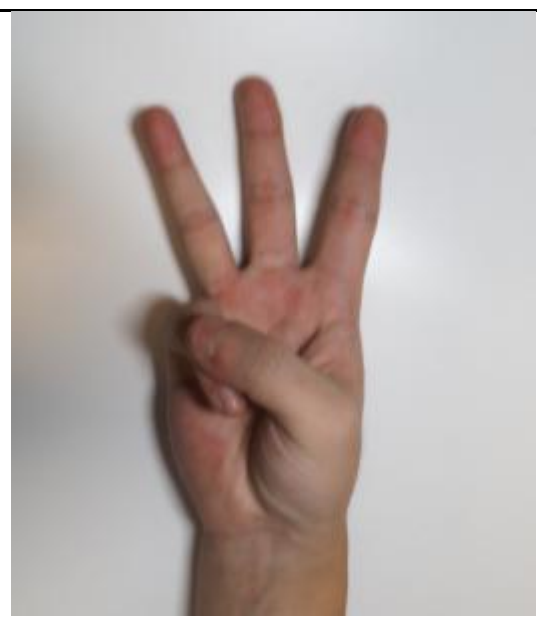
4	CNG 4<br>ASL “4”	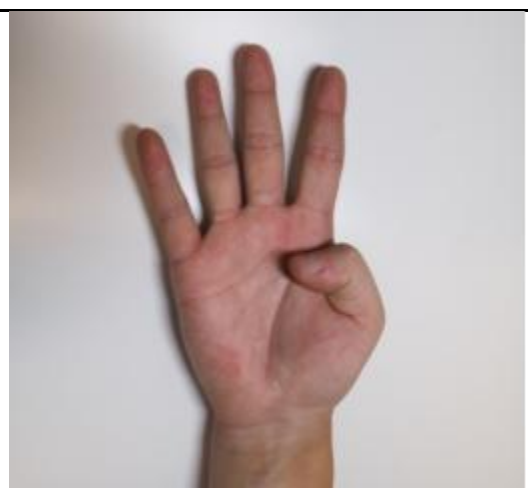	17	ASL “7”	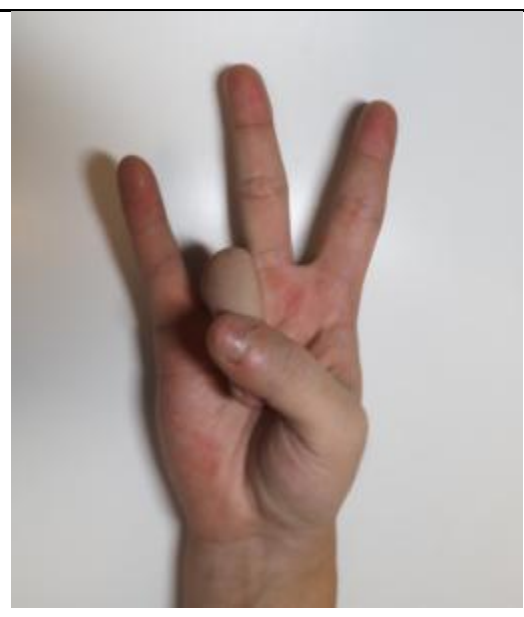

5	CNG 5<br><br>ASL “5”	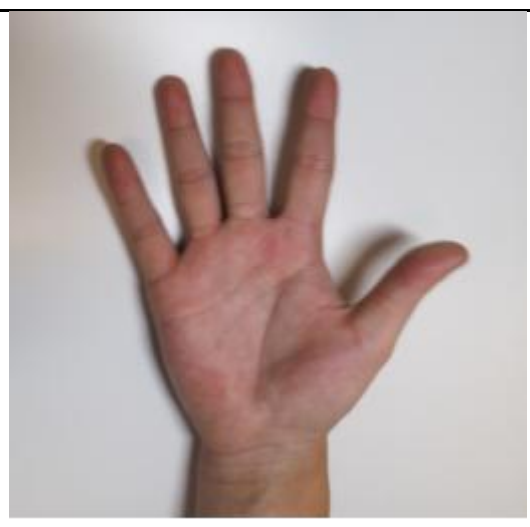	18	ASL “8”	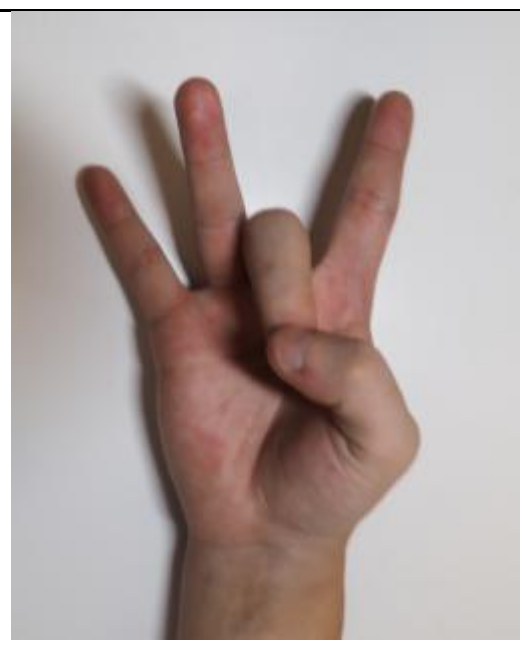
6	CNG 6	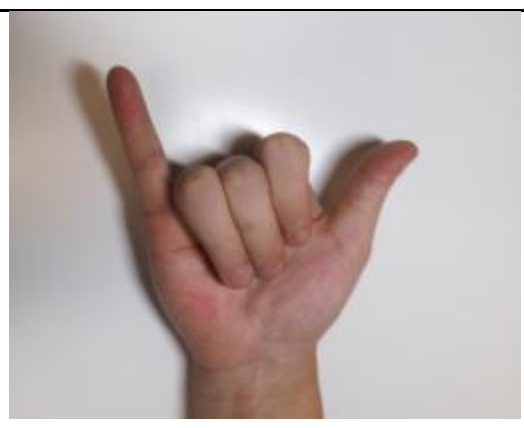	19	ASL “what”	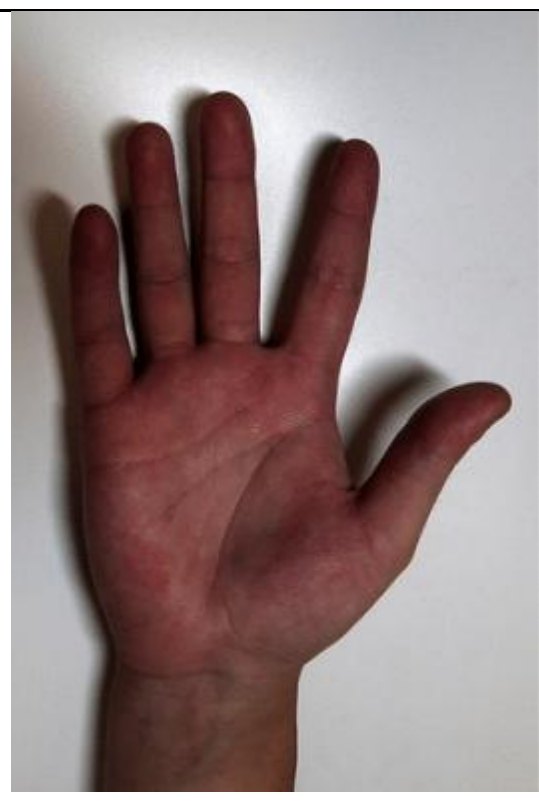
7	CNG 7	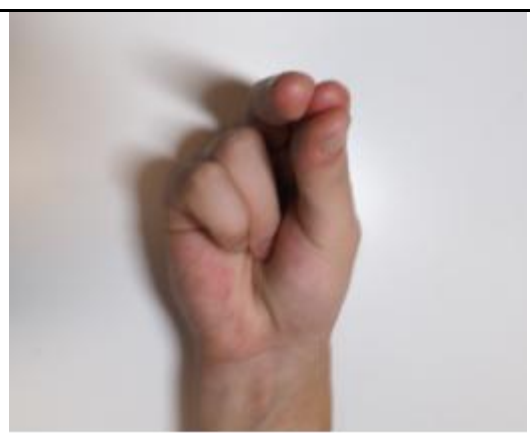	20	ASL “you”	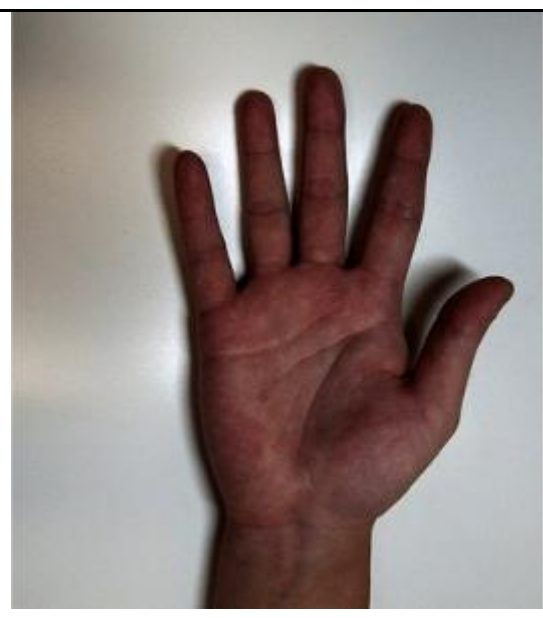
8	CNG 8	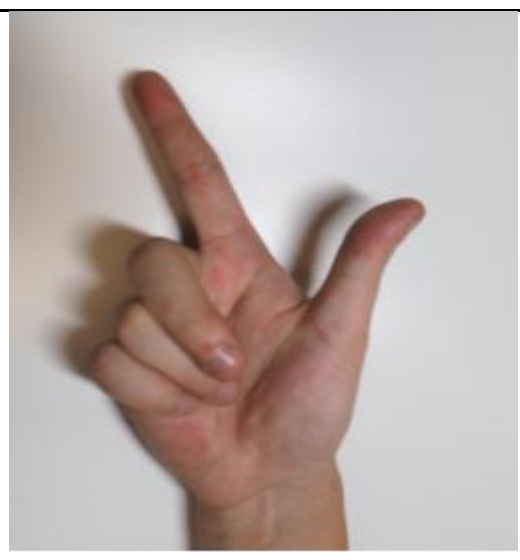	21	ASL “yes”	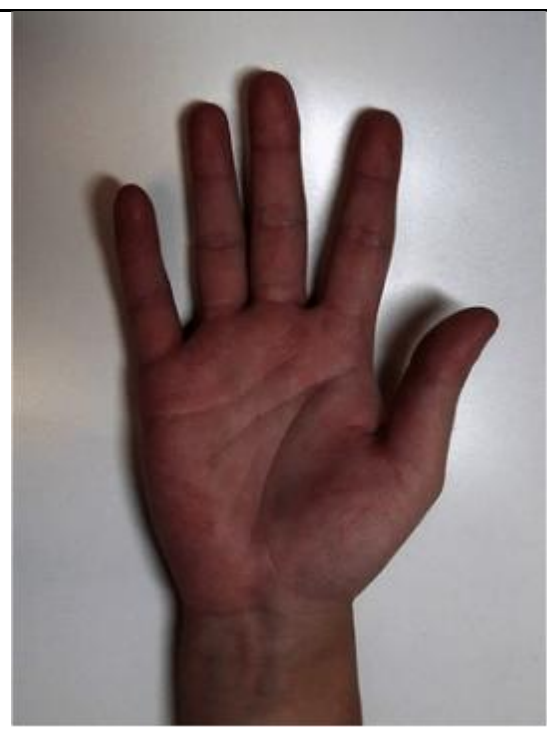

9	CNG 9	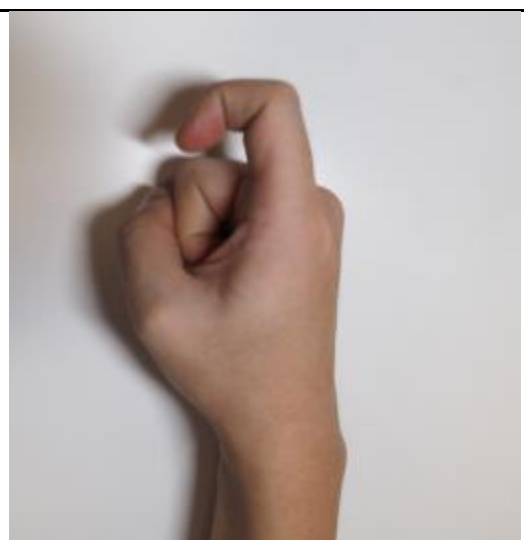	22	ASL “no”	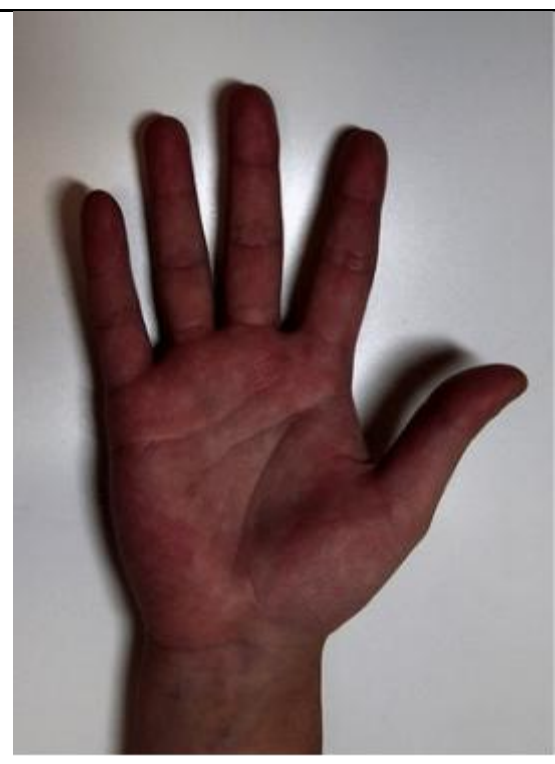
10	CNG 10;<br>ASL “0”	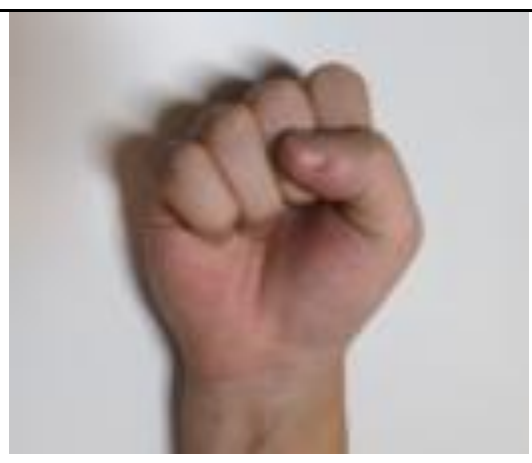	23	ASL<br>“understand”	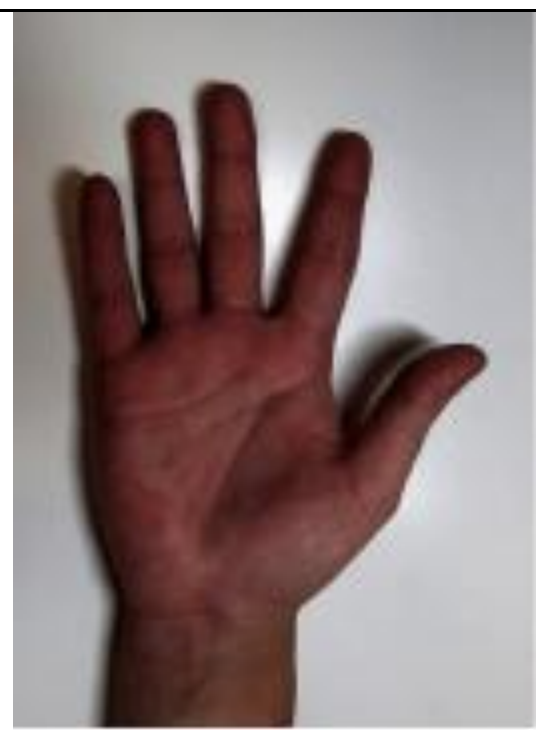
11	Gesture<br>“agree”	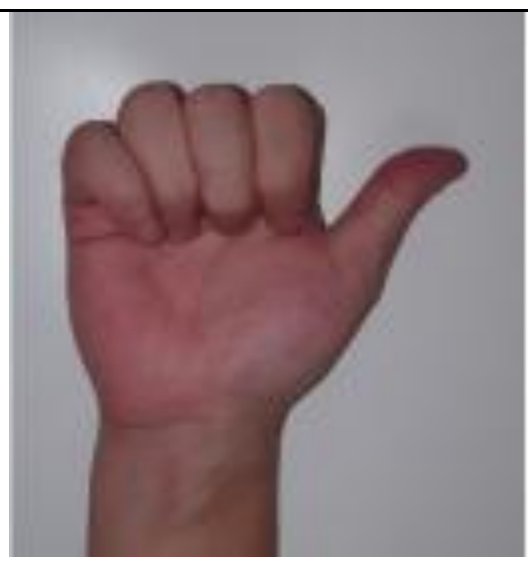	24	ASL “like”	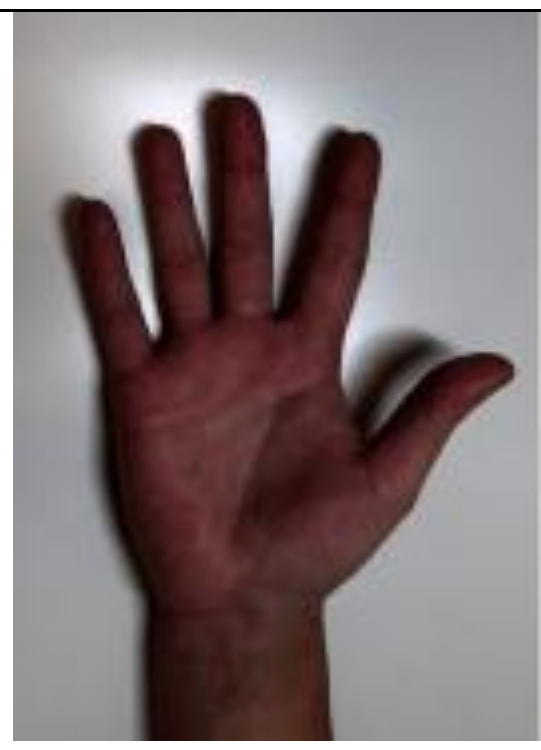
12	Gesture<br>“grasp”	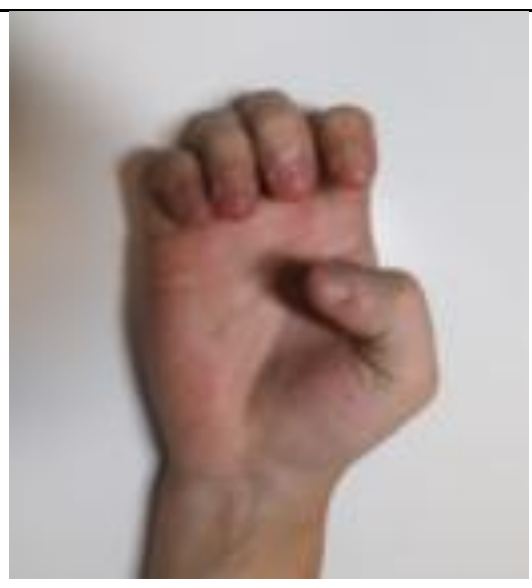	25	ASL “thank<br>you”	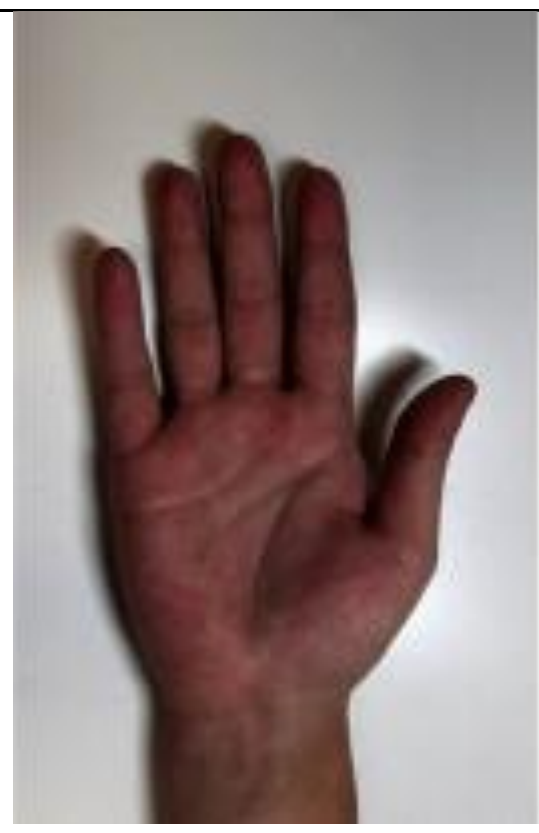

13	Gesture "hand extension"	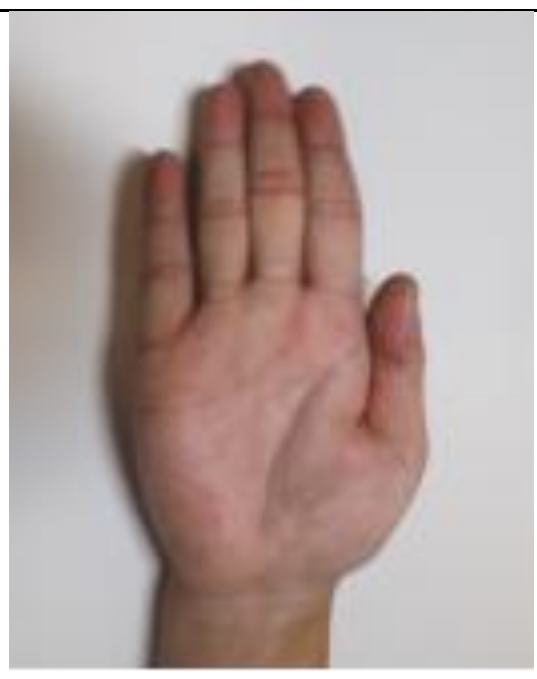	26	ASL "have"	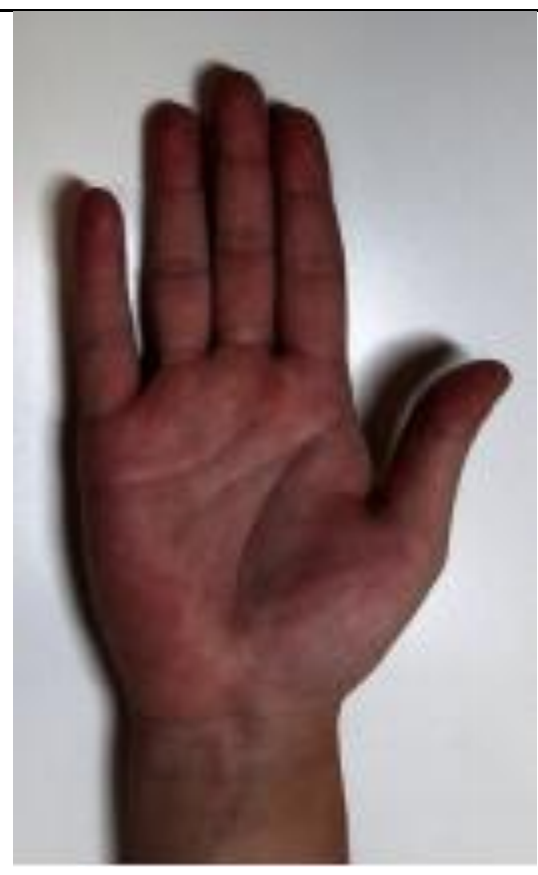

Supplementary Table 5. Prediction accuracy comparison between 10-channel electrode array and 4-channel electrode array.

Group	Prediction Accuracy (%)					
	SVC	SGD	LR	NN	KNN	GPC
Full (10-channel)	86.6 ± 5.06	89.6 ± 4.94	97.4 ± 1.36	93.1 ± 2.02	89.9 ± 3.11	92.5 ± 3.5
Prothesis Control (10-channel)	88.5 ± 4.08	91.5 ± 4.01	95.1 ± 3.65	93.5 ± 4.39	87.7 ± 3.95	92.6 ± 3.32
ASL (10-channel)	91.5 ± 3.72	91.3 ± 7.03	97.8 ± 1.89	96.5 ± 4.36	90.3 ± 5.04	95.8 ± 3.03
Full (4-channel)	70 ± 4.49	67 ± 3.46	83.7 ± 3.69	79.6 ± 4.92	78 ± 2.72	80 ± 3.92
Prothesis Control (4-channel)	72.1 ± 5.09	74.5 ± 6.92	83.7 ± 5.75	77.1 ± 6.09	75.7 ± 5.18	79.2 ± 4.21
ASL (4-channel)	80.1 ± 6.7	78.8 ± 7.16	93.5 ± 4.13	87.8 ± 3.57	84.4 ± 6.95	87.3 ± 5.68

REFERENCES

1. Y. Luo, M. R. Abidian, J.-H. Ahn, D. Akinwande, A. M. Andrews, M. Antonietti, Z. Bao, M. Berggren, C. A. Berkey, C. J. Bettinger, J. Chen, P. Chen, W. Cheng, X. Cheng, S.-J. Choi, A. Chortos, C. Dagdeviren, R. H. Dauskardt, C. Di, M. D. Dickey, X. Duan, A. Facchetti, Z. Fan, Y. Fang, J. Feng, X. Feng, H. Gao, W. Gao, X. Gong, C. F. Guo, X. Guo, M. C. Hartel, Z. He, J. S. Ho, Y. Hu, Q. Huang, Y. Huang, F. Huo, M. M. Hussain, A. Javey, U. Jeong, C. Jiang, X. Jiang, J. Kang, D. Karnaushenko, A. Khademhosseini, D.-H. Kim, I.-D. Kim, D. Kireev, L. Kong, C. Lee, N.-E. Lee, P. S. Lee, T.-W. Lee, F. Li, J. Li, C. Liang, C. T. Lim, Y. Lin, D. J. Lipomi, J. Liu, K. Liu, N. Liu, R. Liu, Y. Liu, Y. Liu, Z. Liu, Z. Liu, X. J. Loh, N. Lu, Z. Lv, S. Magdassi, G. G. Malliaras, N. Matsuhisa, A. Nathan, S. Niu, J. Pan, C. Pang, Q. Pei, H. Peng, D. Qi, H. Ren, J. A. Rogers, A. Rowe, O. G. Schmidt, T. Sekitani, D.-G. Seo, G. Shen, X. Sheng, Q. Shi, T. Someya, Y. Song, E. Stavrinidou, M. Su, X. Sun, K. Takei, X.-M. Tao, B. C. K. Tee, A. V.-Y. Thean, T. Q. Trung, C. Wan, H. Wang, J. Wang, M. Wang, S. Wang, T. Wang, Z. L. Wang, P. S. Weiss, H. Wen, S. Xu, T. Xu, H. Yan, X. Yan, H. Yang, L. Yang, S. Yang, L. Yin, C. Yu, G. Yu, J. Yu, S.-H. Yu, X. Yu, E. Zamburg, H. Zhang, X. Zhang, X. Zhang, X. Zhang, Y. Zhang, Y. Zhang, S. Zhao, X. Zhao, Y. Zheng, Y.-Q. Zheng, Z. Zheng, T. Zhou, B. Zhu, M. Zhu, R. Zhu, Y. Zhu, Y. Zhu, G. Zou, X. Chen, Technology Roadmap for Flexible Sensors. *ACS Nano* **17**, 5211–5295 (2023).

2. C. Xu, Y. Yang, W. Gao, Skin-interfaced sensors in digital medicine: From materials to applications. *Matter* **2**, 1414–1445 (2020).

3. D.-H. Kim, N. Lu, R. Ma, Y.-S. Kim, R.-H. Kim, S. Wang, J. Wu, S. M. Won, H. Tao, A. Islam, K. J. Yu, T. Kim, R. Chowdhury, M. Ying, L. Xu, M. Li, H.-J. Chung, H. Keum, M. McCormick, P. Liu, Y.-W. Zhang, F. G. Omenetto, Y. Huang, T. Coleman, J. A. Rogers, Epidermal electronics. *Science* **333**, 838–843 (2011).

4. S. Xu, J. Kim, J. R. Walter, R. Ghaffari, J. A. Rogers, Translational gaps and opportunities for medical wearables in digital health. *Sci. Transl. Med.* **14**, eabn6036 (2022).

5. J. Heikenfeld, A. Jajack, B. Feldman, S. W. Granger, S. Gaitonde, G. Begtrup, B. A. Katchman, Accessing analytes in biofluids for peripheral biochemical monitoring. *Nat. Biotechnol.* **37**, 407–419 (2019).

6. J. Kim, A. S. Campbell, B. E.-F. De Ávila, J. Wang, Wearable biosensors for healthcare monitoring. *Nat. Biotechnol.* **37**, 389–406 (2019).

7. Y. Wang, H. Haick, S. Guo, C. Wang, S. Lee, T. Yokota, T. Someya, Skin bioelectronics towards long-term, continuous health monitoring. *Chem. Soc. Rev.* **51**, 3759–3793 (2022).

8. J. Min, J. Tu, C. Xu, H. Lukas, S. Shin, Y. Yang, S. A. Solomon, D. Mukasa, W. Gao, Skin-interfaced wearable sweat sensors for precision medicine. *Chem. Rev.* **123**, 5049–5138 (2023).

9. N. Brasier, J. Wang, W. Gao, J. R. Sempionatto, C. Dincer, H. C. Ates, F. Güder, S. Olenik, I. Schauwecker, D. Schaffarczyk, E. Vayena, N. Ritz, M. Weisser, S. Mtenga, R. Ghaffari, J. A. Rogers, J. Goldhahn, Applied body-fluid analysis by wearable devices. *Nature* **636**, 57–68 (2024).

10. G. J. Rodriguez-Rivera, A. Post, M. John, S. Buchan, D. Bernard, M. Razavi, E. Cosgriff-Hernandez, Injectable hydrogel electrodes as conduction highways to restore native pacing. *Nat. Commun.* **15**, 64 (2024).

11. F. Ershad, A. Thukral, J. Yue, P. Comeaux, Y. Lu, H. Shim, K. Sim, N.-I. Kim, Z. Rao, R. Guevara, L. Contreras, F. Pan, Y. Zhang, Y.-S. Guan, P. Yang, X. Wang, P. Wang, X. Wu, C. Yu, Ultra-conformal drawn-on-skin electronics for multifunctional motion artifact-free sensing and point-of-care treatment. *Nat. Commun.* **11**, 3823 (2020).

12. Y. Wang, S. Lee, H. Wang, Z. Jiang, Y. Jimbo, C. Wang, B. Wang, J. J. Kim, M. Koizumi, T. Yokota, T. Someya, Robust, self-adhesive, reinforced polymeric nanofilms enabling gas-permeable dry electrodes for long-term application. *Proc. Natl. Acad. Sci. U.S.A.* **118**, e2111904118 (2021).

13. Z. Zhang, J. Yang, H. Wang, C. Wang, Y. Gu, Y. Xu, S. Lee, T. Yokota, H. Haick, T. Someya, Y. Wang, A 10-micrometer-thick nanomesh-reinforced gas-permeable hydrogel skin sensor for long-term electrophysiological monitoring. *Sci. Adv.* **10**, eadj5389 (2024).

14. J.-C. Hsieh, H. Alawieh, Y. Li, F. Iwane, L. Zhao, R. Anderson, S. I. Abdullah, K. W. Kevin Tang, W. Wang, I. Pyatnitskiy, Y. Jia, J. D. R. Millán, H. Wang, A highly stable electrode with

low electrode-skin impedance for wearable brain-computer interface. *Biosens. Bioelectron.* **218**, 114756 (2022).

15. T. Lan, H. Tian, X. Chen, X. Li, C. Wang, D. Wang, S. Li, G. Liu, X. Zhu, J. Shao, Treefrog-inspired flexible electrode with high permeability, stable adhesion, and robust durability. *Adv. Mater.* **36**, e2404761 (2024).

16. D. Jain, G. Amarpuri, J. Fitch, T. A. Blackledge, A. Dhinojwala, Role of hygroscopic low molecular mass compounds in humidity responsive adhesion of spider's capture silk. *Biomacromolecules* **19**, 3048–3057 (2018).

17. S. Singla, G. Amarpuri, N. Dhopatkar, T. A. Blackledge, A. Dhinojwala, Hygroscopic compounds in spider aggregate glue remove interfacial water to maintain adhesion in humid conditions. *Nat. Commun.* **9**, 1890 (2018).

18. G. Amarpuri, C. Zhang, C. Diaz, B. D. Opell, T. A. Blackledge, A. Dhinojwala, Spiders tune glue viscosity to maximize adhesion. *ACS Nano* **9**, 11472–11478 (2015).

19. T. Ono, T. Sugimoto, S. Shinkai, K. Sada, Lipophilic polyelectrolyte gels as super-absorbent polymers for nonpolar organic solvents. *Nat. Mater.* **6**, 429–433 (2007).

20. J. Chen, S. Wang, J. Peng, J. Li, M. Zhai, New lipophilic polyelectrolyte gels containing quaternary ammonium salt with superabsorbent capacity for organic solvents. *ACS Appl. Mater. Interfaces* **6**, 14894–14902 (2014).

21. K. Manikandan, A. Yelilarasi, S. Saravanakumar, R. H. Althomali, A. Khan, K. M. Abualnaja, D. Alhashmialameer, M. Hussein, The effect of plasticizers on the polypyrrole-poly(vinyl alcohol)-based conducting polymer electrolyte and its application in semi-transparent dye-sensitized solar cells. *Membranes* **11**, 791 (2021).

22. M. Klaassen, D. J. Schipper, M. A. Masen, Influence of the relative humidity and the temperature on the in-vivo friction behaviour of human skin. *Biotribology* **6**, 21–28 (2016).

23. G. P. Nicholson, J. T. Scales, R. P. Clark, M. L. De Calcina-Goff, A method for determining the heat transfer and water vapour permeability of patient support systems. *Med. Eng. Phys.* **21**, 701–712 (1999).

24. T. Huang, J. Chou, T. Sun, S. Hsiung, A device for skin moisture and environment humidity detection. *Sens. Actuators B Chem.* **134**, 206–212 (2008).

25. G. Hanreich, J. Nicolics, M. Mündlein, H. Hauser, R. Chabicovsky, A new bonding technique for human skin humidity sensors. *Sens. Actuators Phys.* **92**, 364–369 (2001).

26. K. Zeng, X. Shi, C. Tang, T. Liu, H. Peng, Design, fabrication and assembly considerations for electronic systems made of fibre devices. *Nat. Rev. Mater.* **8**, 552–561 (2023).

27. Y. Ding, J. Jiang, Y. Wu, Y. Zhang, J. Zhou, Y. Zhang, Q. Huang, Z. Zheng, Porous conductive textiles for wearable electronics. *Chem. Rev.* **124**, 1535–1648 (2024).

28. J. Dong, J. Hou, Y. Peng, Y. Zhang, H. Liu, J. Long, S. Park, T. Liu, Y. Huang, Breathable and stretchable epidermal electronics for health management: Recent advances and challenges. *Adv. Mater.* **36**, e2409071 (2024).

29. B. Zhang, J. Li, J. Zhou, L. Chow, G. Zhao, Y. Huang, Z. Ma, Q. Zhang, Y. Yang, C. K. Yiu, J. Li, F. Chun, X. Huang, Y. Gao, P. Wu, S. Jia, H. Li, D. Li, Y. Liu, K. Yao, R. Shi, Z. Chen, B. L. Khoo, W. Yang, F. Wang, Z. Zheng, Z. Wang, X. Yu, A three-dimensional liquid diode for soft, integrated permeable electronics. *Nature* **628**, 84–92 (2024).

30. G. Chen, X. Xiao, X. Zhao, T. Tat, M. Bick, J. Chen, Electronic textiles for wearable point-of-care systems. *Chem. Rev.* **122**, 3259–3291 (2022).

31. X. Yang, J. Yi, T. Wang, Y. Feng, J. Wang, J. Yu, F. Zhang, Z. Jiang, Z. Lv, H. Li, T. Huang, D. Si, X. Wang, R. Cao, X. Chen, Wet-adhesive on-skin sensors based on metal–organic frameworks for wireless monitoring of metabolites in sweat. *Adv. Mater.* **34**, e2201768 (2022).

32. L. Gao, Z. Wang, Y. Zen, Y. Wang, Q. Ren, Z. Ding, K. Ni, J. Tang, C. Zhang, Y. Wang, Y. Zhou, J. Hui, Q. Lu, R. Liu, X. Zhang, A flexible, bioadhesive, and breathable on-skin battery based on silk fibroin hydrogel for wearable electronics. *Adv. Funct. Mater.* **34**, 2410140 (2024).

33. B. B. Murphy, P. J. Mulcahey, N. Driscoll, A. G. Richardson, G. T. Robbins, N. V. Apollo, K. Maleski, T. H. Lucas, Y. Gogotsi, T. Dillingham, F. Vitale, A gel-free $Ti_3C_2T_x$-based electrode array for high-density, high-resolution surface electromyography, electromyography. *Adv. Mater. Technol.* **5**, 2000325 (2020).

34. H. Yang, S. Ji, I. Chaturvedi, H. Xia, T. Wang, G. Chen, L. Pan, C. Wan, D. Qi, Y.-S. Ong, X. Chen, Adhesive biocomposite electrodes on sweaty skin for long-term continuous electrophysiological monitoring. *ACS Mater. Lett.* **2**, 478–484 (2020).

35. D. Song, G. Ye, Y. Zhao, Y. Zhang, X. Hou, N. Liu, An all-in-one, bioderived, air-permeable, and sweat-stable MXene epidermal electrode for muscle theranostics. *ACS Nano* **16**, 17168–17178 (2022).

36. N. Casado, S. Zendegi, L. C. Tomé, S. Velasco-Bosom, A. Aguzin, M. Picchio, M. Criado-Gonzalez, G. G. Malliaras, M. Forsyth, D. Mecerreyes, Injectable PEDOT:PSS/cholinium ionic liquid mixed conducting materials for electrocardiogram recordings. *J. Mater. Chem. C* **10**, 15186–15193 (2022).

37. G. Li, Y. Liu, Y. Chen, Y. Xia, X. Qi, X. Wan, Y. Jin, J. Liu, Q. He, K. Li, J. Tang, Robust, self-adhesive, and low-contact impedance polyvinyl alcohol/polyacrylamide dual-network hydrogel semidry electrode for biopotential signal acquisition. *SmartMat* **5**, e1173 (2024).

38. J. Lao, Y. Jiao, Y. Zhang, H. Xu, Y. Wang, Y. Ma, X. Feng, J. Yu, Intrinsically adhesive and conductive hydrogel bridging the bioelectronic–tissue interface for biopotentials recording. *ACS Nano* **19**, 7755–7766 (2025).

39. W. Wang, H. Zhou, Z. Xu, Z. Li, L. Zhang, P. Wan, Flexible conformally bioadhesive MXene hydrogel electronics for machine learning-facilitated human-interactive sensing. *Adv. Mater.* **36**, e2401035 (2024).

40. L.-W. Lo, J. Zhao, K. Aono, W. Li, Z. Wen, S. Pizzella, Y. Wang, S. Chakrabartty, C. Wang, Stretchable sponge electrodes for long-term and motion-artifact-tolerant recording of high-quality electrophysiologic signals. *ACS Nano* **16**, 11792–11801 (2022).

41. M. Reis Carneiro, C. Majidi, M. Tavakoli, Multi-electrode printed bioelectronic patches for long-term electrophysiological monitoring. *Adv. Funct. Mater.* **32**, 2205956 (2022).

42. K. Zhang, N. Kang, B. Zhang, R. Xie, J. Zhu, B. Zou, Y. Liu, Y. Chen, W. Shi, W. Zhang, W. Huang, J. Wu, F. Huo, Skin conformal and antibacterial PPy-leather electrode for ECG monitoring. *Adv. Electron. Mater.* **6**, 2000259 (2020).

43. K. He, Z. Liu, C. Wan, Y. Jiang, T. Wang, M. Wang, F. Zhang, Y. Liu, L. Pan, M. Xiao, H. Yang, X. Chen, An on-skin electrode with anti-epidermal-surface-lipid function based on a zwitterionic polymer brush. *Adv. Mater.* **32**, e2001130 (2020).

44. H.-C. Jung, J.-H. Moon, D.-H. Baek, J.-H. Lee, Y.-Y. Choi, J.-S. Hong, S.-H. Lee, CNT/PDMS composite flexible dry electrodesfor long-term ECG monitoring. *IEEE Trans. Biomed. Eng.* **59**, 1472–1479 (2012).

45. L. Eskandarian, A. Toossi, F. Nassif, S. Golmohammadi Rostami, S. Ni, A. Mahnam, M. Alizadeh Meghrazi, W. Takarada, T. Kikutani, H. E. Naguib, 3D-knit dry electrodes using conductive elastomeric fibers for long-term continuous electrophysiological monitoring. *Adv. Mater. Technol.* **7**, 2101572 (2022).

46. Q. Li, G. Chen, Y. Cui, S. Ji, Z. Liu, C. Wan, Y. Liu, Y. Lu, C. Wang, N. Zhang, Y. Cheng, K.-Q. Zhang, X. Chen, Highly thermal-wet comfortable and conformal silk-based electrodes for on-skin sensors with sweat tolerance. *ACS Nano* **15**, 9955–9966 (2021).

47. X. Yan, S. Chen, G. Zhang, W. Shi, Z. Peng, Z. Liu, Y. Chen, Y. Huang, L. Liu, Highly breathable, surface-hydrophobic and wet-adhesive silk based epidermal electrode for long-term electrophysiological monitoring. *Compos. Sci. Technol.* **230**, 109751 (2022).

48. B. M. Li, B. L. Reese, K. Ingram, M. E. Huddleston, M. Jenkins, A. Zaets, M. Reuter, M. W. Grogg, M. T. Nelson, Y. Zhou, B. Ju, B. Sennik, Z. J. Farrell, J. S. Jur, C. E. Tabor, Textile-

integrated liquid metal electrodes for electrophysiological monitoring. *Adv. Healthc. Mater.* **11**, e2200745 (2022).

49. J. Cao, X. Yang, J. Rao, A. Mitriashkin, X. Fan, R. Chen, H. Cheng, X. Wang, J. Goh, H. L. Leo, J. Ouyang, Stretchable and self-adhesive PEDOT:PSS blend with high sweat tolerance as conformal biopotential dry electrodes. *ACS Appl. Mater. Interfaces* **14**, 39159–39171 (2022).

50. S. Hou, D. Lv, Y. Li, Z. Li, M. Liu, X. Yu, Y. Han, Highly stretchable self-adhesive PEDOT:PSS dry electrodes for biopotential monitoring. *ACS Appl. Polym. Mater.* **6**, 7781–7793 (2024).

51. H.-L. Peng, J.-Q. Liu, H.-C. Tian, B. Xu, Y.-Z. Dong, B. Yang, X. Chen, C.-S. Yang, Flexible dry electrode based on carbon nanotube/polymer hybrid micropillars for biopotential recording. *Sens. Actuators Phys.* **235**, 48–56 (2015).

52. X. Zhou, F. Zhao, Y. Guo, B. Rosenberger, G. Yu, Architecting highly hydratable polymer networks to tune the water state for solar water purification. *Sci. Adv.* **5**, eaaw5484 (2019).

53. P. Talik, U. Hubicka, The DSC approach to study non-freezing water contents of hydrated hydroxypropylcellulose (HPC). *J. Therm. Anal. Calorim.* **132**, 445–451 (2018).

54. D. F. Smith, Lithium chloride toxicity and pharmacodynamics in inbred mice. *Acta Pharmacol. Toxicol. (Copenh.)* **43**, 51–54 (1978).

55. G. Hatzis, *O. Rossi*, I. Testiler, G. Dobbins, E. Homan, The effects of lithium chloride exposure on the reproduction of *Caenorhabditis elegans*. *MicroPubl. Biol.* **2024**, 10.17912/micropub.biology.000988 (2024).

56. S. Zhuo, A. Tessier, M. Arefi, A. Zhang, C. Williams, S. K. Ameri, Reusable free-standing hydrogel electronic tattoo sensors with superior performance. *Npj Flex. Electron.* **8**, 49 (2024).

57. X. Nan, S. Mei, L. Xu, J. Chai, G. Wu, X. Zhang, X. Wang, Y. Zhao, F. Lv, L. Gao, Wearable ECG signal sensing system based on easily peelable highly conductive hydrogel. *Sens. Actuators Phys.* **387**, 116453 (2025).

58. W. Chen, X. Shen, H. Liu, L. Du, C. Jiao, L. Li, Q. Wang, Q. Zhang, L. Tang, L. Hu, Y. Fan, Soft and adhesive cardiac patch for electrophysiological and contraction measurement. *Adv. Sci.* **13**, e75098 (2026).

59. H. Lee, J. Jang, J. Lee, M. Shin, J. S. Lee, D. Son, Stretchable gold nanomembrane electrode with ionic hydrogel skin-adhesive properties. *Polymers* **15**, 3852 (2023).

60. Y. Wang, H. Shen, Y. Zhang, Z. Yu, J. Wang, Ultrasoft zwitterionic eutectogels for adaptive and durable sensing at dynamic skin interfaces. *ACS Appl. Mater. Interfaces* **17**, 58774–58782 (2025).

61. Y. Shi, Y. Ding, W. Wang, D. Yu, High performance zwitterionic hydrogels for ECG/EMG signals monitoring. *Colloids Surf. Physicochem. Eng. Asp.* **675**, 132081 (2023).

62. Y. Xue, Z. Lian, F. Zhou, L. Yang, S. Long, D. Yu, W. Wang, Rapidly photo-crosslinked self-adhesive, highly stretchable, and conductive dual-network polyelectrolyte hydrogel for electrocardiogram monitoring application. *Colloids Surf. Physicochem. Eng. Asp.* **740**, 140175 (2026).

63. J. Xia, J. Luo, B. Chang, C. Sun, K. Li, Q. Zhang, Y. Li, H. Wang, C. Hou, High-performance zwitterionic organohydrogel fiber in bioelectronics for monitoring bioinformation. *Biosensors* **13**, 115 (2023).

64. Y. Wang, Z. Wang, Y. Zhang, J. Yang, Z. Zhang, P. Zhou, Y. Xu, Q. Sun, M. Zheng, S. Takao, Y. Wang, A 2.7-μm-thick robust, permeable, and antifreezing hydrogel electrode for long-term ambulatory health monitoring. *Sci. Adv.* **11**, eadt2286 (2025).

65. L. Li, X. Ye, Z. Ji, M. Zheng, S. Lin, M. Wang, J. Yang, P. Zhou, Z. Zhang, B. Wang, H. Wang, Y. Wang, Paintable, fast gelation, highly adhesive hydrogels for high-fidelity electrophysiological monitoring wirelessly. *Small* **21**, e2407996 (2025).

66. Y. Zhao, Q. Zhao, S. Peng, H. Zhou, L. Yang, Robust conductive organohydrogel with adhesive and low- hysteresis properties for all-weather human motion and wireless electrocardiogram sensing. *J. Mater. Chem. C* **11**, 16135–16142 (2023)

67. M. Li, J. Cheng, Y. Hu, L. You, C. Xiong, S. Wang, Gelatin-based conductive hydrogel wearable sensor with breathability and adhesion. *ACS Appl. Polym. Mater.* **7**, 15493–15506 (2025).

68. Y. Li, Y. Gu, S. Qian, Y. Pang, A. Yu, S. Zheng, W. Xia, Y. Liao, B. Liu, S. Liu, Q. Zhao, A stretchable, ionic conductive, and adhesive patch electrode with ultra-low on-skin impedance for electrophysiological signal recording. *Sci. China Inf. Sci.* **68**, 129402 (2025).

69. C. Wang, H. Wang, B. Wang, H. Miyata, Y. Wang, M. O. G. Nayeem, J. J. Kim, S. Lee, T. Yokota, H. Onodera, T. Someya, On-skin paintable biogel for long-term high-fidelity electroencephalogram recording. *Sci. Adv.* **8**, eabo1396 (2022).

70. J. Lin, D. Wang, H. Xue, L. Xia, Z. Li, K. Sun, H. Wang, T. Hamalainen, Y. Li, F. Song, A tough semi-dry hydrogel electrode with anti-bacterial properties for long-term repeatable non-invasive EEG acquisition. *Microsyst. Nanoeng.* **11**, 105 (2025).

71. J.-C. Hsieh, W. He, D. Venkatraghavan, V. B. Koptelova, Z. J. Ahmad, I. Pyatnitskiy, W. Wang, J. Jeong, K. K. W. Tang, C. Harmeier, C. Li, M. Rana, S. Iyer, E. Nayak, H. Ding, P. Modur, V. Mysliwiec, D. M. Schnyer, B. Baird, H. Wang, Design of an injectable, self-adhesive, and highly stable hydrogel electrode for sleep recording. *Device* **2**, 100182 (2024).

72. G. Shen, K. Gao, N. Zhao, Z. Yi, C. Jiang, B. Yang, J. Liu, A novel flexible hydrogel electrode with a strong moisturizing ability for long-term EEG recording. *J. Neural Eng.* **18**, 066047 (2021).

73. K. Zheng, C. Zheng, L. Zhu, B. Yang, X. Jin, S. Wang, Z. Song, J. Liu, Y. Xiong, F. Tian, R. Cai, B. Hu, Machine learning enabled reusable adhesion, entangled network-based hydrogel for long-term, high-fidelity EEG recording and attention assessment. *Nano-Micro Lett.* **17**, 281 (2025).

74. Y. Pan, X. Cui, D. Song, W. Hu, X. Lin, N. Liu, A stretchable and sweat-adhesive 3D graphene eutectogel electrode for EMG monitoring. *Acs Appl. Nano Mater.* **7**, 12064–12071 (2024).

75. H. Zhao, S. Wang, T. Li, Y. Liu, Y. Tang, Z. Zhang, H. Li, Y. Li, X. Li, G. Li, X. Liu, Q. Tian, Z. Liu, Stretchable multi-channel ionotronic electrodes for in situ dual-modal monitoring of muscle–vascular activity. *Adv. Funct. Mater.* **34**, 2308686 (2024).

76. S. Takamatsu, T. Lonjaret, D. Crisp, J.-M. Badier, G. G. Malliaras, E. Ismailova, Direct patterning of organic conductors on knitted textiles for long-term electrocardiography. *Sci. Rep.* **5**, 15003 (2015).

77. P. Leleux, C. Johnson, X. Strakosas, J. Rivnay, T. Hervé, R. M. Owens, G. G. Malliaras, Ionic liquid gel-assisted electrodes for long-term cutaneous recordings. *Adv. Healthc. Mater.* **3**, 1377–1380 (2014).

78. M. Isik, T. Lonjaret, H. Sardon, R. Marcilla, T. Herve, G. G. Malliaras, E. Ismailova, D. Mecerreyes, Cholinium-based ion gels as solid electrolytes for long-term cutaneous electrophysiology. *J. Mater. Chem. C* **3**, 8942–8948 (2015).

79. W. Lin, L. Ai, Y. Wang, X. Yang, J. Liao, Q. Pan, Y. Hong, S. Liu, Z. Long, B. L. Khoo, X. Yao, Z. Yang, Imperceptible liquid metal based tattoo for human-machine interface on hairy skin. *Chem. Eng. J.* **490**, 151595 (2024).

80. B. Wei, Z. Wang, H. Guo, F. Xie, S. Cheng, Z. Lou, C. Zhou, H. Ji, M. Zhang, X. Wang, X. Jiao, *S. Ma*, H.-M. Cheng, X. Xu, Ultraflexible tattoo electrodes for epidermal and in vivo electrophysiological recording. *Cell Rep. Phys. Sci.* **4**, 101335 (2023).

81. Y. Chen, X. Yuan, C. Li, R. Ruan, H. You, Self-healing and self-adhesive substrate-free tattoo electrode. *Materials* **16**, 3499 (2023).

82. Y. Zhang, J. Duan, P. Hong, B. Chen, H. Huang, X. Yin, B. He, P. Zhu, Highly breathable MWCNTs/TPU e-tattoo for physiological signal and motion tracking. *J. Sci. Adv. Mater. Devices* **10**, 101002 (2025).

83. H. J. Yoon, S. R. Shin, J. M. Cha, S.-H. Lee, J.-H. Kim, J. T. Do, H. Song, H. Bae, Cold water fish gelatin methacryloyl hydrogel for tissue engineering application. *PLOS ONE* **11**, e0163902 (2016).

84. Y. Wang, K. Jia, C. Xiang, J. Yang, X. Yao, Z. Suo, Instant, tough, noncovalent adhesion. *ACS Appl. Mater. Interfaces* **11**, 40749–40757 (2019).

85. M. Peleg, An empirical model for the description of moisture sorption curves. *J. Food Sci.* **53**, 1216–1217 (1988).

86. M. D. F. Machado, F. A. R. Oliveira, V. Gekas, R. P. Singh, Kinetics of moisture uptake and soluble-solids loss by puffed breakfast cereals immersed in water. *Int. J. Food Sci. Technol.* **33**, 225–237 (1998).

87. M. Tanaka, S. Kobayashi, D. Murakami, F. Aratsu, A. Kashiwazaki, T. Hoshiba, K. Fukushima, Design of polymeric biomaterials: The “intermediate water concept”. *Bull. Chem. Soc. Jpn.* **92**, 2043–2057 (2019).

88. M. F. Wahid, R. Tafreshi, M. Al-Sowaidi, R. Langari, Subject-independent hand gesture recognition using normalization and machine learning algorithms. *J. Comput. Sci.* **27**, 69–76 (2018).

89. A. F. S. A. Habeeb, Determination of free amino groups in proteins by trinitrobenzenesulfonic acid. *Anal. Biochem.* **14**, 328–336 (1966).

90. J.-H. Lee, T.-H. Lee, K.-S. Shim, J.-W. Park, H.-J. Kim, Y. Kim, S. Jung, Effect of crosslinking density on adhesion performance and flexibility properties of acrylic pressure sensitive adhesives for flexible display applications. *Int. J. Adhes. Adhes.* **74**, 137–143 (2017).
